# Reversible Electrochemical Charging of n-Type Conjugated Polymer Electrodes in Aqueous Electrolytes

**DOI:** 10.1021/jacs.1c06713

**Published:** 2021-09-01

**Authors:** Anna A. Szumska, Iuliana P. Maria, Lucas Q. Flagg, Achilleas Savva, Jokubas Surgailis, Bryan D. Paulsen, Davide Moia, Xingxing Chen, Sophie Griggs, J. Tyler Mefford, Reem B. Rashid, Adam Marks, Sahika Inal, David S. Ginger, Alexander Giovannitti, Jenny Nelson

**Affiliations:** † Department of Physics, Imperial College London, London, SW7 2AZ, United Kingdom; ‡ Department of Chemistry, Imperial College London, London, W12 0BZ, United Kingdom; § Department of Chemistry, Chemistry Research Laboratory, University of Oxford, Oxford, OX1 3TA, United Kingdom; ∥ Department of Chemistry, University of Washington, Seattle, Washington 98195, United States; ⊥ Biological and Environmental Science and Engineering, King Abdullah University of Science and Technology (KAUST), Thuwal, 23955-6900, Saudi Arabia; # Department of Biomedical Engineering, Northwestern University, Evanston, Illinois 60208, United States; ∇ Physical Sciences and Engineering Division, KAUST Solar Center (KSC), King Abdullah University of Science and Technology (KAUST), Thuwal, 23955-6900, Saudi Arabia; ○ Department of Materials Science and Engineering, Stanford University, Stanford, California 94305, United States

## Abstract

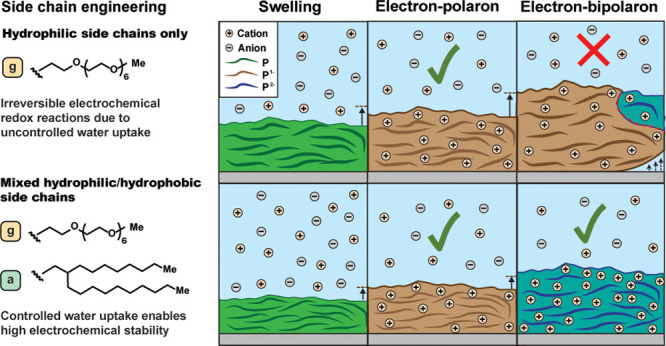

Conjugated polymers achieve redox activity in electrochemical devices by combining redox-active, electronically conducting backbones with ion-transporting side chains that can be tuned for different electrolytes. In aqueous electrolytes, redox activity can be accomplished by attaching hydrophilic side chains to the polymer backbone, which enables ionic transport and allows volumetric charging of polymer electrodes. While this approach has been beneficial for achieving fast electrochemical charging in aqueous solutions, little is known about the relationship between water uptake by the polymers during electrochemical charging and the stability and redox potentials of the electrodes, particularly for electron-transporting conjugated polymers. We find that excessive water uptake during the electrochemical charging of polymer electrodes harms the reversibility of electrochemical processes and results in irreversible swelling of the polymer. We show that small changes of the side chain composition can significantly increase the reversibility of the redox behavior of the materials in aqueous electrolytes, improving the capacity of the polymer by more than one order of magnitude. Finally, we show that tuning the local environment of the redox-active polymer by attaching hydrophilic side chains can help to reach high fractions of the theoretical capacity for single-phase electrodes in aqueous electrolytes. Our work shows the importance of chemical design strategies for achieving high electrochemical stability for conjugated polymers in aqueous electrolytes.

## Introduction

Conjugated polymers bearing hydrophilic side chains are an interesting class of solution-processable, redox-active polymers for applications in biosensors, electrochromics, and electrochemical energy storage,^1−5^ where mixed electronic and ionic conduction is required. The π-conjugated backbone provides electronic conductivity that enables fast electrochemical charging and discharging,^6^ while hydrophilic side chains allow ions to be transported and stored within the polymer film.^7,8^ The chemical structures of the backbone and side chain can be tuned independently to achieve efficient and selective ionic and electronic transport while maintaining high chemical and electrochemical stability.^3,9^

There is a particular need for high-performance electron- and cation-transporting (“n-type”) polymers for use in electrochemical logic circuits, sensors based on electron transfer,^10^ and cathodes for lithium or sodium ion batteries.^4,6,11^ n-Type polymers based on naphthalenetetracarboxylic diimide and bithiophene units (NDI-T2) are promising candidates that have shown good specific capacities and reversible redox reactions in both organic^4,6^ and aqueous electrolytes.^8^ For example, NDI-T2 polymers with branched alkyl side chains can be reduced to their doubly charged, electron bipolaronic state in organic electrolytes to achieve gravimetric capacities of ∼50 mAh/g.^6^ When hydrophilic side chains such as ethylene glycol^8^ or glycol esters^12,13^ are added, NDI-T2-based polymers show promising stability under continuous electrochemical cycling in aqueous electrolytes. The use of hydrophilic side chains also improves the ionic transport properties of conjugated polymer electrodes, leading to rate capabilities sufficient for NDI-T2-based polymers to be used as single-phase electrodes.^3^

Any electrochemical application of redox-active polymers will require a high degree of operational stability, whereby the electrode shows identical electrochemical response to successive charging and discharging cycles. We refer to this concept as electrochemical stability.^14^ In general, capacity decline during cycling may result from several mechanisms, namely, irreversible degradation of the electrode through chemical changes,^15^ parasitic side reactions that reduce the faradaic efficiency of the charge/discharge process,^16^ and mechanical degradation that reduces the ability of the electrode to incorporate electronic and ionic charges through changes in its microstructure or adhesion properties.^17^ Identifying which of the above-mentioned mechanisms dominate the degradation behavior is important, in particular for identifying strategies to improve utilization and cycle life.

Following solution processing, van der Waals and dipole–dipole interactions between the polymer backbones and side chains define the film’s microstructure. To maintain stable ionic and electronic transport properties during electrochemical cycling, any changes in the polymer microstructure that occur during ion intercalation to charge compensate the injected electronic charge carriers must be reversible.^18^ Disruption of polymer packing during insertion and ejection of ions, particularly when carrying large solvation shells, can result in enhanced swelling of the polymer.^19,20^ This is particularly problematic in aqueous electrolytes, where ionic hydration shells can contain up to six water molecules.^21^ As a result, intermolecular interactions and connectivity of polymer chains are weakened, which can negatively impact the charge transport properties and prevent the full utilization of all theoretically available redox-active sites in the bulk. The large uptake of solvent molecules can also accelerate device degradation due to detachment of the electrode material from the current collector or, in extreme cases, the dissolution of the charged polymer.

In addition, glycol side chains show a strong tendency to interact with water molecules via hydrogen bond formation. Thus, while the addition of such hydrophilic side chains to conjugated polymers is expected to increase their ionic conductivity in aqueous electrolytes, it can result in excessive uptake of water molecules that disrupt the physical interactions between the polymer chains. Irreversible changes of the microstructure have been observed, for example, for a polythiophene with hydrophilic side chains after continuous cycling in aqueous electrolytes.^3,22,23^ In order to achieve stability during continuous cycling, both passive swelling (without charging) and active swelling (during the charging) should be controlled. While the use of additives or cross-linkers can be beneficial for preventing film disintegration, it can negatively impact the electronic charge transport properties of the polymer.^24,25^

Previous work has shown that partial substitution of hydrophilic glycol side chains with hydrophobic alkyl side chains can limit the passive swelling of the material, without negatively affecting either the transport properties or the redox potential.^8^ Additional flexibility in the design of partially glycolated polymers that combine mechanical stability with useful electrochemical properties is offered by strategies such as the use of alkyl spacers^13,26^ and varying side chain length.^23^ Since redox potentials are influenced by local dielectric properties, the influence of hydrophilic side chains and associated water uptake on the degree of charge screening of the polymer is also important to understand. Despite the clear potential for the design of operationally stable polymers with attractive redox properties via side chain engineering, limited work to date has addressed the relationship between swelling and redox properties of conjugated polymers for electrochemical devices.

In this work, we study the electrochemical redox activity of conjugated polymers based on NDI-T2 donor–acceptor polymers with hydrophilic side chains and demonstrate the importance of side chain engineering in improving their redox activity and stability when operated in aqueous electrolytes with low oxygen concentrations. For polymers containing only hydrophilic side chains, we observe rapid capacity loss during the continuous cycling of polymer electrodes in aqueous electrolytes. To address this electrochemical instability, we tune the properties of the polymer by partial substitution of hydrophilic (glycol) side chains with hydrophobic (alkyl) side chains to control water uptake during electrochemical charging. When charging the polymers to the doubly reduced, electron bipolaronic state, this substitution also greatly improves their gravimetric capacity. To explore the degradation mechanism of polymers during charging and discharging, we study the relationship between the polarity of the side chains and water uptake by quantifying the passive and active swelling. The results demonstrate that swelling of the polymers can be controlled by chemical design and greatly enhance the redox performance and stability of electron-transporting polymers.

## Results

### Interaction of the Polymers with Water-Based Electrolytes

**Figure 1:**
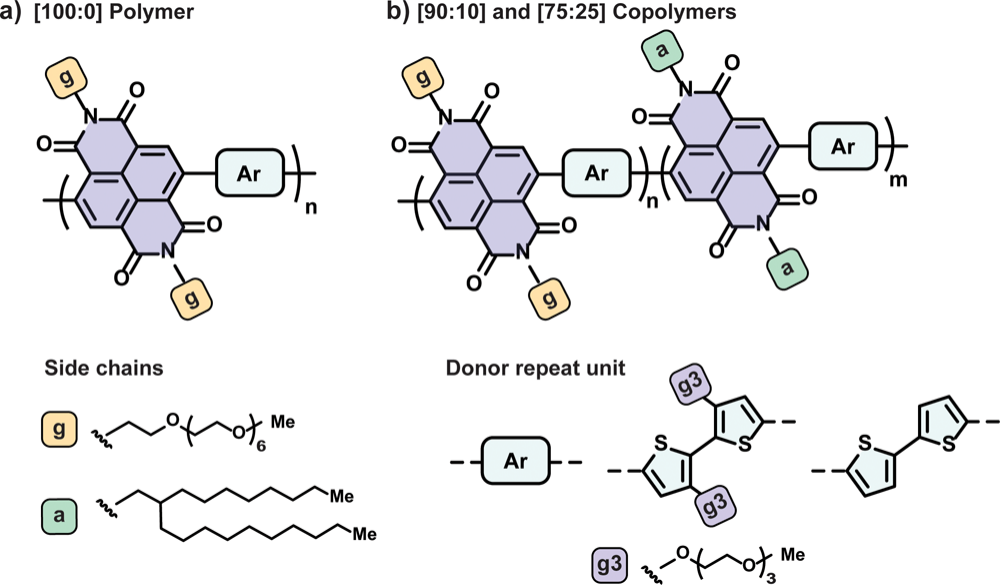
Chemical structure of the polymers (a) p([100:0]NDI-T2) and p([100:0]NDI-g3T2) and (b) p([90:10]NDI-g3T2/T2), p([90:10]NDI-g3T2/T2), and p([75:25]NDI-g3T2). The side chains attached to the NDI units are hydrophilic side chains based on a heptakis ethylene glycol side chain (glycol, g) or hydrophobic side chains based on a branched C_8_,C_10_-alkyl side chain (alkyl, a).

To evaluate the impact of side chain substitution on the electrochemical stability of the polymer in aqueous electrolytes, we synthesized a series of three polymers based on 3,3′-dialkoxy(triethylene glycol)bithiophene (g3T2) and naphthalenetetracarboxylic diimide (NDI) units with varying ratios of hydrophilic to hydrophobic side chains. The donor unit of the synthesized polymers is functionalized with triethylene glycol side chains (g3T2), whereas the NDI unit is functionalized with either linear heptakis ethylene glycol side chains (referred to as “g7”) or a mixture of g7 and branched C_8_,C_10_-alkyl side chains (referred to as “a”). The [g7:a] side chain ratio is controlled during the synthesis, where random polymers with ratios of [90:10] and [75:25] were synthesized following a previously reported protocol for other NDI-T2 polymers.^8^ We limit our attention to polymers with no more than 25% alkyl-substituted side chains, as previous studies showed that thin films of polymers with a higher density of hydrophobic side chains have reduction potentials cathodic to the hydrogen evolution reaction (HER).^8^ We investigate the properties of the polymers relative to the [100:0] analog containing exclusively g7 side chains on the NDI unit, which was previously studied as an electrode material for water-based electrochemical storage devices.^3^ Additionally, we compare the properties of the p([g7:a]NDI-g3T2) polymer series to previously reported NDI-T2 polymer series p([g7:a]NDI-T2).^8^ From this series, we selected two polymers: p([100:0]NDI-T2) with 100% glycol chain density, previously referred to as P-100,^8^ and p([90:10]NDI-T2) with 90% glycol chain density, previously referred to as P-90.^8^ The chemical structures of all polymers are illustrated in Figure 1. All polymers have good solubility in chloroform, allowing fabrication of electrochemical devices from solution, and are insoluble in water-based electrolytes, allowing characterization of their redox performance in aqueous electrolytes. The p([g7:a]NDI-g3T2) polymers have similar ionization potentials (IPs) of 4.9–5.0 eV and electron affinities (EA) of 4.1 eV (Table 1). The number-average molecular weights (*M*_n_) and dispersities [*Đ*] of the p([g7:a]NDI-g3T2) series were found to be 12.5 kDa [2.05] for p([75:25]NDI-g3T2), 13.6 kDa [2.01] for p([90:10]NDI-g3T2), and 6.6 kDa [1.57] for p([100:0]NDI-g3T2), as determined by gel permeation chromatography. The comparable EA, IP, and *M*_n_ across the series suggest that any differences in electrochemical stability and utilization are due to differences in the side chain structure rather than energetic or molecular weight effects. We emphasize that the conjugated polymers reported here are air-sensitive when charged to the electron polaronic and bipolaronic states, resulting in parasitic side reactions during the operation in ambient conditions [Figure S28, Supporting Information (SI)]. We thus must exclude oxygen from the electrolyte to avoid electron-transfer reactions between the reduced polymer and molecular oxygen (oxygen reduction reaction (ORR)).^16^ We study the performance of the materials in dilute (0.1 M) aqueous NaCl solution, since it is less corrosive than electrolytes with high salt concentration, while we acknowledge that variations of the electrolyte concentration can have an impact on the swelling of the polymer electrode.^20^

**Table 1:** Properties of the Polymer Series

				swelling (%)^d^			
polymer	*M*_n_ (kDa) [*Đ*]^a^	IP^b^ (eV)	EA^c^ (eV)	AFM	QCM-D	*E*_red,aq_^e^ vs Ag/AgCl (V)	capacity (mAh/g) [utilization of theoretical capacity (%)]^f^
p([100:0]NDI-g3T2)	6.6 [1.57]	4.9	4.1	106	110	–0.21 (−0.25)	2.2 [5.7]^f^
p([90:10]NDI-g3T2)	13.6 [2.01]	5.0	4.1	89	95	–0.21 (−0.22)	21.7 [56.3]
p([75:25]NDI-g3T2)	12.5 [2.05]	5.0	4.1	27	16	–0.23 (−0.20)	27.7 [71.3]
p([100:0]NDI-T2)	7.2 [1.25]^8^	5.5^8^	4.2^8^	157	105	–0.08 (−0.16)	8.1 [16.2]
p([90:10]NDI-T2)	7.8 [1.59]^8^	5.6^8^	4.2^8^	59	37	–0.09 (−0.18)	34.5 [68.6]

a Number-average molecular weight (*M*_n_) and dispersity (*Đ*) (see section 3, SI)

b IP was measured by photoelectron spectroscopy in air (PESA).

c EA was measured by cyclic voltammetry of polymer thin films on ITO substrates in 0.1 M tetrabutylammonium hexafluorophosphate (TBAPF_6_) in acetonitrile (Figure S17, SI).

d Additional data on swelling is reported in the Supporting Information (section 7).

e Potential for the reduction onset during the first cycle determined by CV measurements of the polymer thin films in 0.1 M NaCl aqueous solution (50 mV/s) at low oxygen concentration (values in parentheses represent the potential for the second cycle).

f Theoretical capacity was calculated using Faraday’s law (Table S1, SI).

The ability of redox-active polymers to swell in aqueous electrolytes was previously correlated with enhanced ion transport properties of the polymer electrodes.^27^ The water uptake of pristine polymer thin film was studied by quartz crystal microbalance with dissipation monitoring (QCM-D) and atomic force microscopy (AFM) measurements in 0.1 M NaCl aqueous electrolytes. QCM-D quantifies the passive swelling by monitoring changes in the polymer mass using the vibrational frequency of a quartz crystal that acts as the substrate for the polymer thin film, while AFM measurements reveal changes in the thickness of pristine and swollen films after exposure to the aqueous electrolyte. As expected, we observe the largest fractional increase in mass or thickness upon exposure to a 0.1 M NaCl aqueous solution for polymers that contain glycol side chains only {110% mass uptake by QCM-D and 106% thickness increase by AFM for p([100:0]NDI-g3T2)} and more limited film expansion for polymers containing 10% {95% by QCM and 89% by AFM for p([90:10]NDI-g3T2)} or 25% {16% by QCM-D and 27% by AFM for p([75:25]NDI-g3T2)} alkyl side chains. A similar trend is observed for p([g7:a]NDI-T2)) polymers {105% by QCM-D and 157% by AFM for p([100:0]NDI-T2) and 37% by QCM-D and 59% by AFM for p([90:10]NDI-T2)}. Encouragingly, the trends shown by AFM and QCM-D measurements are in strong agreement. The results are summarized in Table 1 and experimental details are provided in the Supporting Information (sections 5 and 7).

### Electrochemical Charging in Water-Based Electrolytes

**Figure 2:**
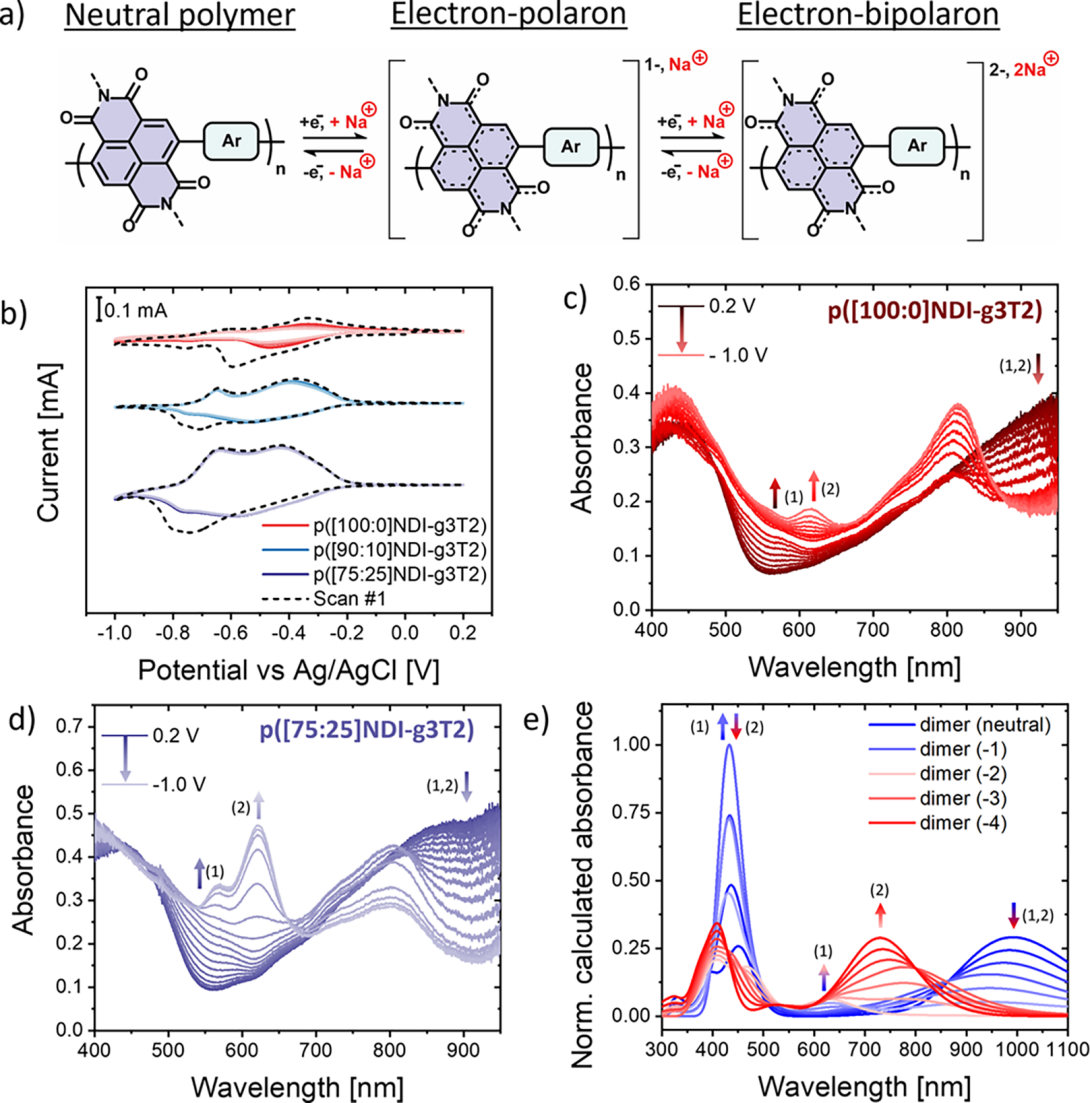
Electrochemical characterization of the polymers. (a) Electrochemical charging/discharging half-reactions of NDI-Ar polymers (Ar = T2 or g3T2) to the electron-polaron and electron-bipolaron. (b) CV measurements of the polymers {(p([100:0]NDI-g3T2) (red), p([90:10]NDI-g3T2) (blue), and p([75:25]NDI-g3T2) (purple)} in 0.1 M NaCl aqueous electrolytes with a scan rate of 50 mV/s with low O_2_ concentration, showing five cycles (the first cycle is shown with dashed lines and the shaded area highlights the loss in current after one charging cycle). Spectroelectrochemical measurements of (c) p([100:0]NDI-g3T2) and (d) p([75:25]NDI-g3T2), monitoring the evolution of the absorption spectrum during the first charging cycle of polymer thin films on ITO substrates between 0.2 and −1.0 V vs Ag/AgCl with a scan rate of 50 mV/s {results for p([90:10]NDI-g3T2) are presented in Figure S23, SI}. The notations (1) and (2) refer to the spectral changes associated with the polaron (0 to −0.5 V vs Ag/AgCl) and bipolaron states (−0.5 to −1.0 V vs Ag/AgCl), respectively. (e) Normalized calculated absorbance spectra using TD-DFT for the dimer (gNDI-gT2)_2_ in the neutral and successively reduced states (up to four electrons per dimer).

To investigate the relationship between the side chain distribution of the polymers, the degree of swelling, and the electrochemical redox behavior, we conducted cyclic voltammetry (CV) experiments of both thin polymer films [Figures 2b and S18 (SI), scan rate of 50 mV/s] and thick electrodes fabricated by drop-casting on conductive paper (Figure S19, SI, scan rate of 5 mV/s) in a 0.1 M NaCl aqueous solution between 0.2 and −1.0 V vs Ag/AgCl. Within this voltage range, the neutral NDI polymers are reduced to form the electron-polaron and electron-bipolaron (Figure 2a). As shown in Figure 2b and summarized in Table 1, the charging (reduction) of all p([g7:a]NDI-g3T2) polymers occurred at potentials <−0.2 V vs Ag/AgCl (Table 1). As is typical for films of conjugated polymers with a relatively disordered microstructure that undergo volumetric charging, the cyclic voltammogram of the polymers is relatively broad and featureless. Upon continuous cycling of the thin polymer electrodes, p([100:0]NDI-g3T2) shows the lowest electrochemical stability with a decrease of 61% (second scan) and 73% (fifth scan) of the initial capacity [Figures 2b and S18 (SI)]. In comparison, the electrochemical stability of the polymers containing alkyl side chains, p([90:10]NDI-g3T2), and p([75:25]NDI-g3T2), are significantly improved, losing only 23% (second scan) and 29% (fifth scan) of the initial capacity for p([90:10]NDI-g3T2) and 15% (second scan) and 18% (fifth scan) for p([75:25]NDI-g3T2) (Figure 2b). Their relative reversibility is retained even when subject to continuous charging and discharging for 100 scans (Figure S18, SI). The low electrochemical stability of polymers with glycol side chains may be related to a loss of structural integrity of the polymer film when reduced, possibly involving excessive water penetration that likely weakens the intermolecular interaction and limits the accessibility of redox-active sites in the bulk. Interestingly, for p([100:0]NDI-g3T2)), the polymer with the largest fraction of glycol side chains, we also observed dissolution of the film upon charging to the electron bipolaronic state (Figure S20, SI).

The polymers with high electrochemical stability show two reduction peaks that can be assigned to the singly (electron-polaron) and doubly (electron-bipolaron) reduced state.^3,6^ For these polymers, a shift in the reduction onset to more positive potentials is observed between the first and second charging cycle, as shown in Figure 2b (dashed lines) and Table 1, as well as for thick electrodes shown in Figure S19 (SI). Such changes can be attributed to the rearrangement of polymer chains as well as to the bias-dependent swelling of the redox-active polymer, in accordance with previous studies.^17^ Interestingly, this effect is reproducibly observed, where a precycled electrode shows similar changes in the cyclic voltammograms during the first and second scans after allowing the swollen films to dry in ambient conditions (Figure S27, SI). The finding supports the idea that the changes for the first and second CV scans are related to reversible microstructural changes and water uptake, rather than permanent chemical degradation of the polymers.

As summarized in Table 1, the QCM-D and AFM measurements show that the polymer with only glycol side chains swells more than the polymers with mixed glycol and alkyl side chains. As the apparent redox potentials of the polymers appear to be correlated with the degree of swelling, it is possible that the redox potential is directly influenced by the amount of water the side chains take up through its effect on the dielectric properties of the polymer’s environment. Previous studies have shown that hydrophilic side chains tend to increase the amount of water around the polymer and that the effect of a more hydrophilic environment is to shift the reduction potential of conjugated polymers to less-negative values.^3,28^

This difference can be rationalized via the effect of water addition in increasing the dielectric constant of the polymer environment, which helps to screen additional charges on the polymer and shifts the reduction potential positively.^29^ These results, as well as the experimental findings, highlight the importance of water uptake by the polymer thin films in lowering the energy to reduce the polymer. This swelling effect helps to explain the observation of a less negative reduction potential of the mixed alkyl/glycol polymers after the first scan is completed, as shown for thin and thick electrodes [Figures 2b and S19 (SI)] and summarized in Table 1, while the effect is not detectable for the all-glycol polymers due to their low electrochemical stability.

To further investigate the charging processes of the polymers, we conducted spectroelectrochemical measurements of the polymer thin films and monitored the changes of the absorption spectrum during the first charging/discharging cycle. We observed a significant difference within the p([g7:a]NDI-g3T2) polymer series. For all polymers, the evolution of a broad absorption between 550 and 700 nm and loss in the intensity of the absorption band at >800 nm were both observed at higher potentials (0 to −0.5 V vs Ag/AgCl), indicating the formation of the polaron (one electron per repeat unit), as shown in Figures 2 and S24 (SI). For polymers containing mixed glycol/alkyl side chains, p([90:10]NDI-g3T2/T2) (Figures S23 and 24, SI) and p([75:25]NDI-g3T2) (Figure 2d), charging to potentials <−0.5 V vs Ag/AgCl reveals formation of a well-defined absorption peak with λ_max_ = 620 nm. We assign these changes in the absorption spectrum to bipolaron formation on the basis of previous findings for NDI polymers with zwitterionic side chains.^3^

To interpret these findings, we use time-dependent density functional theory (TD-DFT) calculations. We calculate spectra for a dimer (gNDI-gT2)_2_ in its neutral and successively reduced states up to the bipolaron and compare them with the spectra obtained when applying the indicated potentials. Computational details are included in the Supporting Information (section 9). The calculated absorption spectra are presented in Figure 2e, showing strong agreement with the experimental absorption spectra. The defined peak for p([90:10]NDI-g3T2) and p([75:25]NDI-g3T2) at potential <−0.6 V vs Ag/AgCl (λ_max_ = 620 nm) can be attributed to the bipolaronic state (charge of −4 spread across the dimer), as it correlates with the electron-bipolaron feature at 700 nm in the calculated spectrum. We can also assign the peak at 800 nm as the polaron state because, in the spectroelectrochemical measurements for p([75:25]NDI-g3T2), it initially rises and then decays upon charging. As demonstrated by the calculated spectra for an alkylated analogous dimer (Figures S38–S40, SI), replacing glycol side chains on the NDI unit with alkyl side chains only marginally affects the computed absorption spectra. This insensitivity of the absorption band to the nature of the side chain is expected since both side chains are attached via saturated ethyl groups. Therefore, the variations in spectra during the charging are not a result of the polymer chemical structure alone. Rather, the differences in observed spectra reflect the different degrees of charging achieved for the different polymers under equivalent levels of electrochemical bias.

### Electrochemical Stability in Water-Based Electrolytes

**Figure 3:**
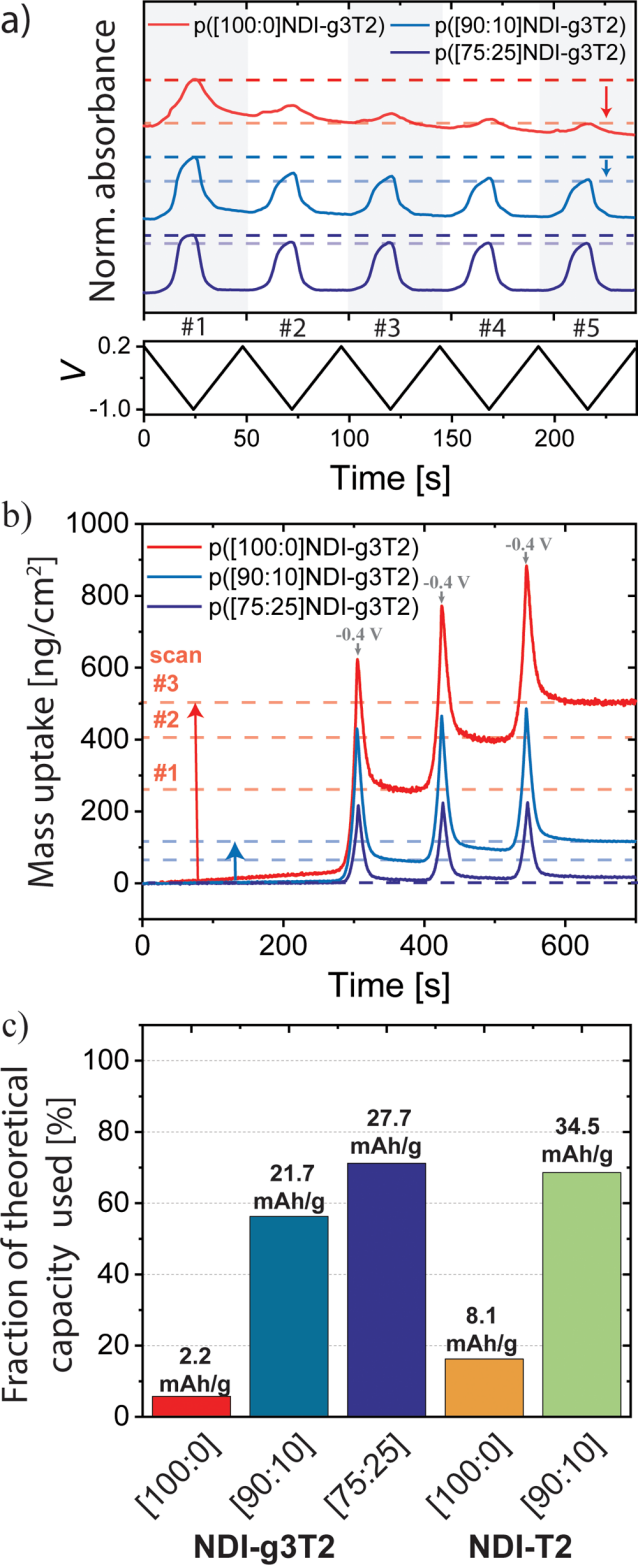
Assessing the electrochemical stability of polymer electrodes during continuous cycling. (a) Monitoring the changes in the intensity of the bipolaron absorption peak at 620 nm during continuous charging and discharging between 0.2 and −1.0 V vs Ag/AgCl in 0.1 M NaCl for five cycles of p([100:0]NDI-g3T2) (red), p([90:10]NDI-g3T2) (blue), and p([75:25]NDI-g3T2) (purple). Note: The intensity of the bipolaron absorption peak of p([100:0]NDI-g3T2) (Figure 2c) is lower in intensity, to begin with, compared to that of p([90:10]NDI-g3T2) or p([75:25]NDI-g3T2). The absorbance was normalized to the maximum of the absorption peak at 620 nm. (b) eQCM-D measurements of p([100:0]NDI-g3T2) (red), p([90:10]NDI-g3T2) (blue), and p([75:25]NDI-g3T2) (purple) in inert conditions when scanning the potential from 0.2 to −0.4 V vs Ag/AgCl (10 mV/s) for three scans at low oxygen concentration (the corresponding cyclic voltammograms are reported in the Figure S29, SI). Note: A lower degree of charging is achieved for the polymer p([75:25]NDI-g3T2) due to the increase of the reduction potential. (c) Summary of the gravimetric capacities of thick polymer electrodes on conductive paper at a C-rate of 30, presenting the individual capacities of the electrodes (mAh/g) and the fraction of the theoretical capacity used (%) (additional data and CV measurements are presented in Figure S19, SI). Note: The polymer electrodes were pre-cycled before conducting the CP measurents (100 CV scans with a scan rate of 5 mV/s, Figure S19, SI).

The findings from the CV and spectroelectrochemical measurements [Figures 2 and S23 and S24 (SI)] suggest that the greater reversibility of CV behavior under electrochemical cycling can be correlated with the intensity of the bipolaronic absorption feature in the electrochemically reduced polymer film. Accordingly, we attempt to evaluate the electrochemical stability of the polymers by monitoring changes in the absorption features assigned to the bipolaron during continuous charging and discharging between 0.2 and −1.0 V vs Ag/AgCl in 0.1 M NaCl aqueous solution. To visualize the changes of the bipolaronic absorption feature, we plot the intensity of the bipolaron absorption peak at λ_max,bipolaron_ = 620 nm during the cycling of polymer thin films for five consecutive cycles {Figures 3a {p([g7:a]NDI-g3T2)} and S24 {p([g7:a]NDI-T2)} (SI)}.

As shown in Figure 3a, p([75:25]NDI-g3T2) achieves the highest retention of the bipolaronic absorption feature, where a drop of only 12% (second scan) and 14% (fifth scan) is observed relative to the first scan. For p([90:10]NDI-g3T2), a larger drop in absorbance of 25% (second scan) and 36% (fifth scan) is observed, followed by p([100:0]NDI-g3T2) that achieves the lowest retention of the bipolaronic peak, losing 54% (second scan) and 93% (fifth scan). A similar trend is observed for p([90:10]NDI-T2) and p([100:0]NDI-T2) (Figure S24, SI), where reversible bipolaron formation is only observed for p([90:10]NDI-T2), however not for p([100:0]NDI-T2). The findings are in agreement with the CV measurements, where p([100:0]NDI-g3T2) and p([100:0]NDI-T2) achieve the lowest retention of the capacity during continuous cycling (Figure S19, SI). All polymers experience changes in the absorption spectra after the first charging cycle (Figures S25 and S26, SI) that result in a drop of the polymer absorbance, with fewer changes for polymers with mixed alkyl/glycol side chains compared to their all-glycol analogs. The findings indicate that polymers with a higher fraction of hydrophilic side chains experience larger physical changes where uncontrolled swelling inhibits the formation of the doubly reduced state in the polymer.

To rationalize these findings, we further investigated the electrochemical stability of the polymers by electrochemical QCM (eQCM-D) measurements. We begin the measurements after the initial passive swelling phase has reached an equilibrium state and monitor the mass changes of polymer thin films during the cycling to quantify the amount of water taken up during the charging and discharging process. We perform continuous charging and discharging between 0.2 and −0.4 V vs Ag/AgCl for three cycles with a scan rate of 10 mV/s for the p([g7:a]NDI-g3T2) polymers (Figure 3b) and p([g7:a]NDI-T2) polymers (Figure S30, SI).

As expected, a mass increase is detected during the charging (assigned to the uptake of cations) followed by a mass decrease during the discharging cycle (release of cations). Interestingly, in addition to the mass uptake during the charging, we observe a net increase in mass upon consecutive charge and discharge cycles, the magnitude of which is strongly related to the polarity of the polymer side chains (Figure 3b). For p([100:0]NDI-g3T2), a large drift of the baseline is observed after each charging/discharging cycle, corresponding to a net mass uptake of 343% after the first cycle, which increases to 604% after only three cycles (Figure S29, SI). In comparison, p([90:10]NDI-g3T2) and p([75:25]NDI-g3T2) show a smaller drift of the baseline with a net mass increase of 87% (after the first cycle) to 162% (after three cycles) and of 11% to 32%, respectively. We assign the irreversible fraction of mass uptake to the uptake of water molecules that once have entered the bulk of the polymer can interact with the glycol side chains via hydrogen bond formation, as previously described.^19,27^ Additional mass uptake can also stem from the trapping of cations, which was not investigated in the present study. When combined with the CV and spectroelectrochemistry measurements, the findings from eQCM support the claims that the substitution of alkyl side chains lowers the swelling and mass uptake during continuous cycling, enhancing the electrochemical stability of the polymers and allowing reversible charging to the electron bipolaronic states in aqueous electrolytes. Interestingly, when applying potentials <−0.4 V vs Ag/AgCl, the eQCM-D results show significant changes for all polymers (Figure S31, SI). We hypothesize that applying potentials <−0.4 V vs Ag/AgCl may result in the transition of the polymers to a hydrogel-like state, as previously observed for polythiophenes with hydrophilic side chains,^22^ which changes the mechanical properties of the polymers significantly. As we are uncertain about the interpretation of the results at very negative charging potentials (Figure S31, SI), we only evaluate the results at potentials greater than −0.4 V vs Ag/AgCl.

To further highlight the importance of achieving reversible water uptake during continuous cycling of the polymers in aqueous electrolytes, we studied the gravimetric capacities of the two NDI polymer series. We conducted chronopotentiometry (CP) measurements on conductive paper electrodes with mass loadings of the polymer >1.0 mg/cm^2^ and employed the polymers as single-phase electrodes (no binders or additives) in a 0.1 M NaCl aqueous solution with low O_2_ concentrations. We chose to precycle the polymer electrodes for 100 cycles between 0.2 and −1.0 V vs Ag/AgCl to evaluate the performance of the polymers in their swollen state (Figure S19, SI). Figure 3c summarizes the gravimetric capacities of the polymers in mAh/g measured during the discharging cycle at a C-rate of 30. We also report the utilization of the theoretical gravimetric capacity based on a maximum of two electrons per repeat unit in the dry film. For both polymer series, we observed that polymers with mixed alkyl/glycol side chains can utilize a significantly higher fraction of their theoretical capacity compared to the polymers exclusively containing glycol side chains (Table 1). The all-glycol polymers achieve a gravimetric capacity of 2.2 mAh/g [5.7%] {p([100:0]NDI-g3T2)} and 8.1 mAh/g [16.2%] {p([100:0]NDI-T2)}, while partly alkylated polymers reached 21.7 mAh/g [56.3%] {p([90:10]NDI-g3T2)}, 34.5 mAh/g [68.6%] {p([90:10]NDI-T2)}, and 27.7 mAh/g [71.3%] {p([75:25]NDI-g3T2)}. While all polymers achieve higher gravimetric capacities during the first charging cycle compared to the following cycles (Figures S19, SI), we observe a rapid degradation for polymers with high fractions of hydrophilic side chains, in particular for thick electrodes on paper electrodes. The reason for achieving only small fractions of the theoretical capacity for p([100:0]NDI-g3T2) and p([100:0]NDI-T2) is likely linked to the uptake of large amounts of water with signs of dissolution of the charged polymer. The findings show the importance of side chain engineering for utilizing the theoretical capacities of the polymers during the continuous cycling of the electrodes in aqueous electrolytes.

**Figure 4:**
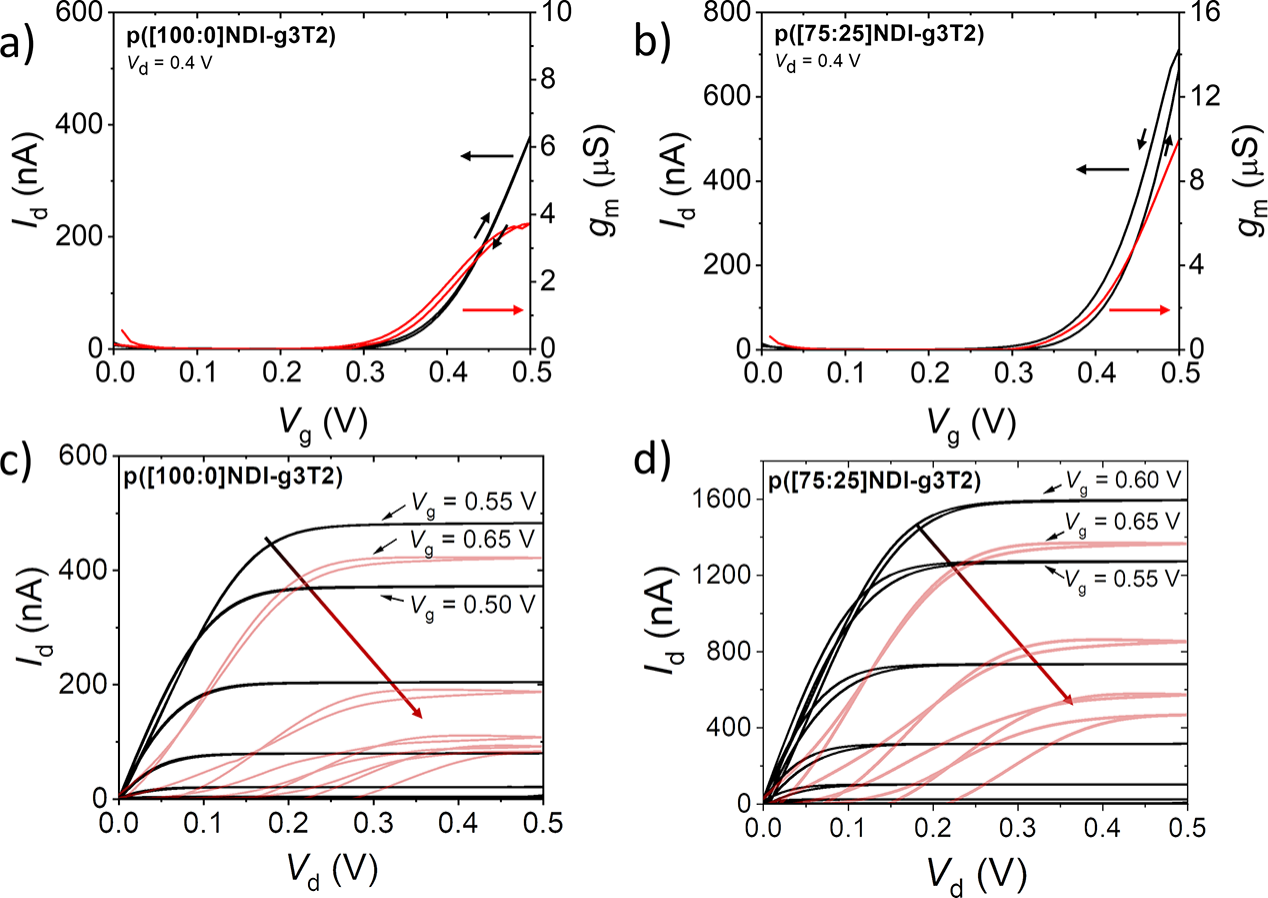
OECT operation of p([g7:a]NDI-g3T2) polymers in 0.1 M NaCl with low O_2_ concentration with *W* × *L* of 100 μm × 10 μm, showing the transfer curves in the stable regime for (a) p([100:0]NDI-g3T2) and (b) p([75:25]NDI-g3T2) with a scan rate of 200 mV/s and *V*_d_ = 0.4 V. The corresponding output curves in the stable (black lines) and unstable (red lines) regimes are shown for (c) p([100:0]NDI-g3T2) and (d) p([75:25]NDI-g3T2). Additional information and device data for p([90:10]NDI-g3T2), p([90:10]NDI-T2), and p([100:0]NDI-T2) are presented in Figure S32 (SI).

Finally, we studied the impact of the swelling on the coupled electronic/ionic charge carrier transport properties by fabricating organic electrochemical transistors (OECTs) with the polymers as channel materials. All devices showed reproducible transistor behavior up to gate voltages (*V*_g_) of 0.5 V (equivalent to −0.5 V vs Ag/AgCl), with nearly ideal saturation behavior, as evident from the transfer and output curves [Figures 4 and S32 (SI)]. We analyzed the threshold voltages of the devices and find that p([90:10]NDI-g3T2) shows the lowest threshold voltage (*V*_th_), of 321 ± 5 mV, followed by p([100:0]NDI-g3T2) (327 ± 5 mV) and p([75:25]NDI-g3T2) (357 ± 3 mV) (Table S2 and Figure S34, SI). While the increased alkyl side chain content of p([75:25]NDI-g3T2) improved the redox stability due to decreased swelling, it also diminished ion transport, as evidenced by the transfer curves revealing sweep rate-dependent hysteresis that was significantly larger than that of p([90:10]NDI-g3T2) and p([100:00]NDI-g3T2). The mobility–capacitance products (μC*)^30^ (Table S2) were comparable to previous reports of NDI-T2 copolymers.^8,12^

While, as noted above, the electrochemical stability of electrodes at more-negative potentials (vs Ag/AgCl) is improved for polymers containing alkyl side chains, the OECT characteristics did not reflect this trend. Device degradation is observed at potentials greater than *V*_g_ = 0.5 V (equivalent to <−0.5 V vs Ag/AgCl) for all polymers manifested when charging the polymer beyond the polaronic state [Figures 4 and S32 and S33 (SI)]. The degradation of the device begins at *V*_g_ of 0.50 and 0.55 V for p([100:0]NDI-g3T2) and p([90:10]NDI-g3T2), respectively, while p([75:25]NDI-g3T2) shows slightly improved stability, with degradation starting at *V*_g_ > 0.55 V. In all cases, the degradation is cumulative with cycling, leading to an irreversible loss of channel conductivity.

Interestingly, transfer curves collected at (or near) the saturation condition (*V*_d_ ≥ *V*_g_ – *V*_th_) extended cycling stability up to *V*_g_ = 0.8 V (beyond the ∼0.5 V limit observed in output curves and linear regime transfer curves). Also, all the materials in the series displayed a channel conductance peak at *V*_g_ ∼ 0.65 V (Figure S33, SI). While this conductance peak phenomenon is common in polymeric electrochemical transistors,^31^ the cause is contested. From the spectroelectrochemistry, the conductance peak here coincides with the conversion of electron-polarons to electron-bipolarons, suggesting that the electron bipolaronic states of the polymer are less mobile compared to the polaronic states.^17^ Further analyses of the OECT characteristics are reported in section 8 of the Supporting Information. The findings show that ion intercalation and associated water uptake are greatly affecting the electronic charge transport properties.

## Discussion

We have used two series of NDI-T2 polymers bearing different fractions of hydrophilic and hydrophobic side chains as a case study to identify the mechanisms that limit the stability of n-type polymers under repeated electrochemical cycling (i.e., their electrochemical stability). Using QCM measurements, AFM measurements, electrochemical and spectroelectrochemical measurements, and density functional theory calculations we could show that electrode swelling is beneficial for achieving low charging potentials, however, only to the extent of avoiding mechanical disintegration of the polymer due to irreversible swelling during the charging in aqueous electrolytes. Our findings show that a small fraction of hydrophobic side chains can significantly limit the swelling of the polymers in aqueous electrolytes and can greatly improve the electrochemical stability of polymer electrodes during continuous cycling. Encouragingly, our findings show that side chain engineering of the polymers enables reversible access to the doubly reduced, bipolaronic state in pH-neutral aqueous electrolytes.

**Figure 5:**
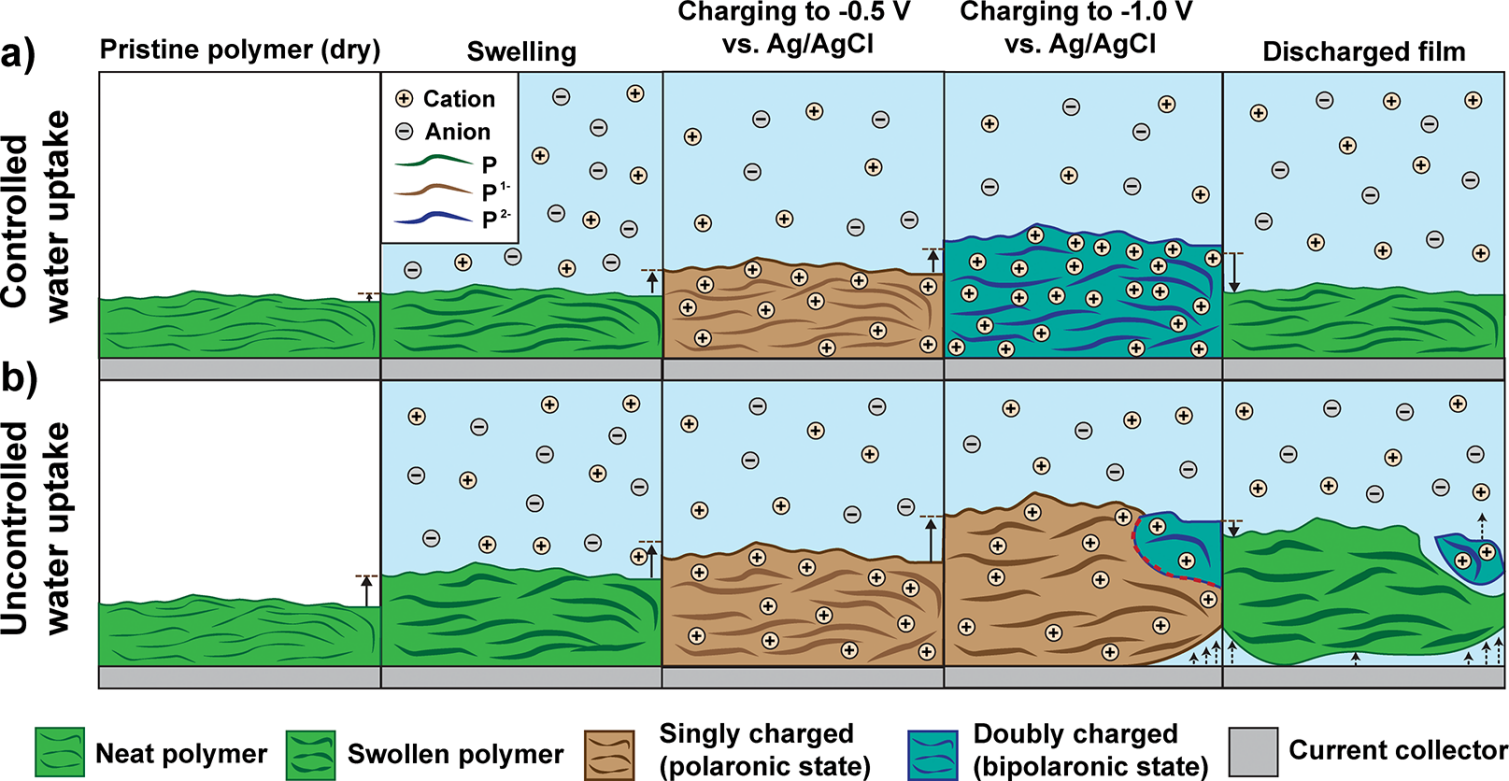
Illustration of the charging mechanism of redox-active polymers with (a) controlled water uptake {e.g., polymers with mixed hydrophilic/hydrophobic side chains such as p([90:10]NDI-g3T2) and p([75:25]NDI-g3T2)} and (b) uncontrolled water uptake {e.g., polymers with hydrophilic side chains only such as p([100:0]NDI-g3T2)} to the electron-polaron (equivalent to applying −0.5 V vs Ag/AgCl) and electron bipolaronic state (equivalent to applying −1.0 V vs Ag/AgCl). For simplicity reasons, the uptake of ions during the swelling is not illustrated and any anion migration between electrolyte and the bulk of the polymer is omitted.^34^ The swelling of the polymer film is illustrated by the thickness of the polymer chain, where the color change represents the charging of the neutral polymer (green) to the electron polaronic (brown) and electron bipolaronic (blue) states. The solid-lined arrows represent the swelling of the polymer electrode, and the dashed-line arrows indicate detachment of the swollen or charged polymer from the current collector. The red dashed line illustrates mechanical stress that results in the detachment of the polymer.

Previously, electrochemical reduction to the bipolaron state of NDI polymers in aqueous electrolytes has only been achieved when employing side chains with permanent charges (zwitterions),^3^ which negatively impact the electronic charge transport properties.^3^ For NDI-T2 polymers containing hydrophilic side chains based on ethylene glycol, the bipolaron has not yet been reported to form reversibly.^3,12,13^ We find for both polymer series that adding hydrophobic side chains allows the bipolaron state to be reached reversibly, despite differences in the structure of the donor unit and overall side chain density. To characterize the changes that occur during continuous electrochemical cycling, we monitor changes in the absorption spectrum of the reduced states and mass taken up, as well as the charge injected. We find a strong correlation between the reversible appearance of the bipolaronic state during charging and the reversibility of the film swelling during cycling. These findings are illustrated in Figure 5 for a polymer that undergoes controlled (Figure 5a) and uncontrolled swelling (Figure 5b) during the cycling. We suggest that uncontrolled swelling, as observed for polymers with hydrophilic side chains only, disrupts the physical interaction of the polymer chains and prevents the transformation between the singly to doubly charged states. The uncontrolled uptake of large amounts of water molecules explains the observed detachment of the polymer from the current collector or the dissolution of the doubly reduced polymer. We thus hypothesize that commonly observed irreversible redox reactions of electron-transporting polymers^3,13^ or small molecules containing hydrophilic side chains^32^ may be linked to irreversible uptake of water during charging, rather than chemical degradation due to irreversible changes of the chemical structure.

In summary, our results show that polymers that incorporate water molecules to a limited extent (Figure 5a) achieve high retention of the capacity during continuous cycling and allow >70% of the theoretical capacity to be utilized in aqueous electrolytes at a C-rate of 30. Previous work showed that polymers with a large fraction of glycol side chains {e.g., p([100:0]NDI-g3T2)} can achieve high electrochemical stability, however, only during the charging to the polaronic state.^3^ This shows the strong correlation between the degree of charging of the repeat units and the volume of electrolyte admitted into the bulk of the polymer, with a larger uptake of electrolyte resulting in larger changes of the microstructure (due, for example, to additional water uptake by shedding water molecules from the hydration shell) and leading to lower electrochemical stability during cycling.^3^

We demonstrate that the developed concept is applicable beyond NDI polymers, as we also observe improved electrochemical stability for other electron-transporting conjugated polymers based on conformationally locked lactam units when hydrophilic side chains are partly replaced with hydrophobic side chains (Figure S21, SI). Further progress in the specific capacity of polymer electrodes will be achieved by optimizing the ratio of charge to mass of the repeat unit. To test this hypothesis, we investigated the role of the side chains on the fast and reversible charging of single-phase electrodes by studying poly(benzimidazobenzophenanthroline) (BBL),^33^ a conjugated polymer without side chains. In comparison to the NDI-T2/g3T2 polymers, we observe a significantly larger dependency of the gravimetric capacity with C-rate, showing a drop of the capacity from 51 to 6.5 mAh/g when increasing the C-rate from 1 to 100 C (Figure S22, SI). A single-phase electrode with BBL achieves only 16.5% of its theoretical capacity at a C-rate of 30 (Figure S22, SI), demonstrating the importance of hydrophilic side chains for achieving fast ion transport into the bulk of the polymer to access a large fraction of redox-active sites in single-phase electrodes. Thus, while decreasing the length of the redox-inactive side chain is an obvious pathway for improving the gravimetric capacity, our work shows that the tuning of the local environment is highly important for unlocking high gravimetric capacities, especially for single-phase electrodes.

## Conclusion

In summary, we show that side chain engineering of redox-active materials is an effective route to control and limit swelling of electrode materials when charging the electrode to highly charged states with more than one electronic charge carrier per repeat unit. By adding hydrophobic alkyl side chains, we show that the uptake of water molecules can be controlled and limited, which improves the electrochemical stability. We highlight that controlled swelling paves the way to achieve full capacity utilization of the polymer in aqueous electrolytes, namely, the reversible formation of the doubly reduced, bipolaronic state without mechanical disintegration of the polymer. Furthermore, our findings show the advantage of tuning the local environment of conjugated polymers by attaching hydrophilic (ion-transporting) side chains to enable rapid charging of single-phase electrodes to achieve high fractions of their theoretical gravimetric capacity. Our study shows the importance of chemical design strategies to tune the local environment of redox-active polymers to reveal their full potential in electrochemical devices in aqueous electrolytes.

## Supporting Information Available

Details on the monomer and polymer syntheses and characterizations, UV–vis spectra, GPC traces, AFM and QCM-D measurements, electrochemical characterization of the polymers and fabricated electrodes, spectroelectrochemical measurements, eQCM-D measurements, OECT measurements, and DFT and TDDFT calculations (PDF)
