# Supplementary material for: Reversible Electrochemical Charging of n-Type Conjugated Polymer Electrodes in Aqueous Electrolytes
Source: J Am Chem Soc. 2021 Sep 1;143(36):14795–805. doi: 10.1021/jacs.1c06713 (PMC8447255; doi:10.1021/jacs.1c06713)
Supplement: Supplementary file 1 — ja1c06713_si_001.pdf [file ja1c06713_si_001.pdf]

SUPPORTING INFORMATION

Reversible electrochemical charging of n-type conjugated polymer electrodes in aqueous electrolytes

Anna A. Szumska [a#], Iuliana P. Maria [b,c#], Lucas Q. Flagg [d], Achilleas Savva [e], Jokubas Surgailis [e], Bryan D. Paulsen [f], Davide Moia [a], Xingxing Chen [g], Sophie Griggs [b,c], J. Tyler Mefford [i], Reem B. Rashid [f], Adam Marks[b,c], Sahika Inal [e], David S. Ginger [d], Alexander Giovannitti [a,i*] and Jenny Nelson [a*]

[a] Department of Physics, Imperial College London, London , SW7 2AZ, United Kingdom

[b] Department of Chemistry, Imperial College London, London , W12 0BZ, United Kingdom

[c] Department of Chemistry, Chemistry Research Laboratory, University of Oxford, Oxford, OX1 3TA, United Kingdom

[d] Department of Chemistry, University of Washington, Seattle, Washington, 98195, United States

[e] Biological and Environmental Science and Engineering, King Abdullah University of Science and Technology (KAUST), Thuwal, 23955-6900, Saudi Arabia

[f] Department of Biomedical Engineering, Northwestern University, Evanston, Illinois, 60208, United States

[g] Physical Sciences and Engineering Division, KAUST Solar Center (KSC), King Abdullah University of Science and Technology (KAUST), Thuwal, 23955-6900, Saudi Arabia

[i] Department of Materials Science and Engineering, Stanford University, Stanford, California, 94305, United States

# A.A.S. and I.M. contributed equally to this paper

*Email: ag19@Stanford.edu, jenny.nelson@imperial.ac.uk

Table of content

1 Experimental Section 4

2 Synthesis and characterization 8

2.1 General synthesis 8

2.2 Monomer and polymer synthesis 8

2.3 Summary of the synthesis of monomer bg_4N and polymers $p(bg_4NC_{16}N)$ and $p(bg_4Ng_3N)$ 12

3 GPC measurements 19

4 Optical measurements 19

5 Swelling analysis 20

5.1 AFM analysis 20

5.1.1 Summary of AFM measurements 20

5.1.2 Redrying of the p([g7:a]NDI-g3T2) series 21

5.2 QCM-D analysis 21

6 Electrochemical and spectroelectrochemical measurements 22

6.1 CV measurements in organic electrolytes 22

6.2 CV measurements in aqueous electrolytes 23

6.2.1 Thin films on ITO coated glass substrates 23

6.2.2 Thick electrodes on conductive paper electrodes 24

6.3 Performance of other conjugated polymers 25

6.4 Evaluating the concept across other polymer backbones 25

6.4.1 Poly(benzimidazobenzophenanthroline) (BBL) 26

6.5 Calculation of the theoretical capacity of the polymers 27

6.5.1 Spectroelectrochemical measurements of p([90:10]NDI-g3T2) 28

6.5.2 Spectroelectrochemical measurements of the p([g7:a]NDI-T2) series 29

6.5.3 Difference of the absorption spectrum of p([100:0]NDI-g3T2), p([90:10]NDI-g3T2) and p([75:25]NDI-g3T2) during continuous charging 30

6.5.4 Difference of the absorption spectrum of p([100:0]NDI-T2) and p([90:10]NDI-T2) during continuous charging 31

6.5.5 Comparison of charging of a neat and pre-cycled polymer thin film 32

6.6 Retention experiments of the reduced polymer with low and high O_2 concentrations 33

7 eQCM-D measurements 34

7.1 eQCM-D measurements of the p([g7:a]NDI-g3T2) series charging to –0.4 V vs. Ag/AgCl 34

7.2 eQCM-D measurements of the p([g7:a]NDI-T2) series charging to –0.4 V vs. Ag/AgCl 35

7.3 eQCM-D measurements of the p([g7:a]NDI-g3T2) series to <–0.4 V vs. Ag/AgCl ... 36

8 Organic electrochemical transistor (OECT) measurements 37

9 DFT and TDDFT simulations 39

9.1 Method 39

9.2 Results 39

9.3 Summary of the calculations 42

9.4 Coordinates of optimized structures 44

9.4.1 (gNDI-gT2)$_2$ Neutral optimized structure in water (PCM) 44

9.4.2 (aNDI-gT2)$_2$ Neutral optimized structure in water (PCM) 46

9.4.3 (gNDI-T2)$_2$ Neutral optimized structure in water (PCM) 48

9.4.4 (aNDI-T2)$_2$ Neutral optimized structure in water (PCM) 50

1 Experimental Section

Synthesis of the polymers: The synthesis of p([75:25]NDI-g3T2), p([90:10]NDI-g3T2), p([100:0]NDI-g3T2) is reported in the section 2 of the supporting information, following a previously reported procedure.[1,2] Polymers p([100:0]NDI-T2) and p([90:10]NDI-T2) were synthesized as previously reported.[1] The synthesis of monomer (bg4N) and polymers p(bg_4Ng_3N) and p($bg_4NC_{16}N$) are also reported in section 2 of the supporting information. Poly(benzimidazobenzophenanthroline) (BBL) was purchased from Sigma Aldrich.

Thickness measurements using AFM:

Atomic force microscopy (AFM) images were taken using an Asylum Research Cypher-ES. The thicknesses of the dry films were measured by imaging across a razor blade scratch in tapping mode using 325 kHz AFM probes (HQ:NSC15/Pt, µMasch). Hydrated thickness was determined by depositing 100 µL of degassed 100 mM NaCl solution onto the sample and imaging over the same scratch in contact mode using ContGB-G tips (BudgetSensors) with a force set point of 6 nN. Line profiles (4 µm wide) were taken perpendicular to the scratch for plotting. All thicknesses were averaged from at least 6 locations on at least 2 different films.

Electrochemical quartz crystal microbalance with dissipation of energy (eQCM-D) measurements:

eQCM-D measurements were performed using a Q-sense analyzer (QE401, Biolin Scientific). Swelling measurements were performed as follows. First, we recorded the QCM-D response of the bare Au sensors in the air, followed by injection of a 0.1 M NaCl aqueous solution into the chamber. This resulted in large shifts in the frequency (*f*) and dissipation of energy (*D*), due to the density differences between air and electrolyte, which must be excluded from the swelling percentage calculation. The measurements were then stopped, the sensors were removed, and the polymer layers were spin-coated directly on the same sensors from 4 mg/mL solutions in chloroform at 1000 rpm. The absolute *f* value for each polymer coated sensor was obtained both in air and in a 0.1 M NaCl aqueous solution, after the *f* signal was perfectly flat (i.e., Δf <0.5 Hz/2 min), assuring that the system is in equilibrium. We then compared the absolute difference in *f* for multiple overtones between the bare sensor and the polymer coated sensors, both in air and in the electrolyte, by using the function "stitched

data" of Q-soft software. This function compares the selected datasets based on the raw frequencies measured and excludes the effect of the different densities between the two media. Thus, the difference of the f values of the stitched data is directly analogous to the mass absorbed by the polymer (m) in both media, which is calculated by using the Sauerbrey equation:

$$\Delta m = \frac{-17.7}{\mathrm{n}} \Delta f_{\mathrm{n}} \quad (1)$$

The thickness of the films in air and the electrolyte is calculated accordingly (Figure S6). For eQCM-D measurements, we coupled the Q-sense electrochemistry module, comprising a three-electrode setup, with an Autolab PGstat128N potentiostat. Since the films become soft and take up a significant amount of water under applied potential, we used the Kelvin-Voigt viscoelastic model to fit the data and to quantify the mass, as described in detail elsewhere.[3] For eQCM-D measurements with low O_2 concentrations, the electrolyte solution was purged with N_2 within a custom-made chamber, equipped with microtubes for N_2 inlet and electrolyte solution outlet. After intense purging with N_2 for more than 1.5 hours, a 0.1 M NaCl aqueous solution was pumped into the eQCM-D chamber, using a peristaltic pump at a constant flow of 50 µl/min and without exposure to ambient air. Throughout the measurements, the solution was kept under light N_2 purging to avoid dilution of ambient O_2 during the measurements.

Electrochemical characterization:

Electrochemical measurements were carried out using a potentiostat (IviumStat or Biologic SP-300 Potentiostat) with a three-electrode setup, using a Ag/AgCl reference electrode (BASi, 3 M NaCl or eDAQ leakless Ag/AgCl reference electrode) and a Pt mesh as the counter electrode. Thin thin films were prepared by spin coating on indium-doped tin oxide (ITO) glass substrates. The films were immersed in the electrolyte that contains 0.1 M NaCl aqueous solution where the concentration of oxygen was lowered by sparging the electrolyte with inert gases (Ar or N_2). NDI-T2/g3T2, p($bg_3NC_{16}N$) and p(bg_4Ng_3N) polymer electrodes on conductive paper were prepared by dissolving the polymers in chloroform and drop-casting on conductive paper substrates (AvCarb P50T). Electrodes with BBL were prepared by dissolving BBL in methanesulfonic acid (10 mg/mL) and drop casting 100 µL of the polymer solution on the paper electrode, followed by the addition of 500 µL of deionized (DI) water.

The polymer gel was first washed with DI water, followed by drying the electrode at 100 °C for 4 h while applying vacuum. A paper electrode was fabricated with pg2T-TT[4] (polymer loading of 5 mg) that acted as the counter electrode for the three-electrode measurements to limit side reactions with the electrolyte and formation of reactive intermediates during continuous charging of the paper electrodes in 0.1 M NaCl aqueous electrolyte. Cyclic voltammetry (CV) measurements were carried out at the indicated scan rates (50 mV/s for polymer thin film electrodes, 5 mV/s for paper electrodes) for 5 or 100 scans. Chronopotentiometry (CP) measurements were carried out at various current densities to measure the gravimetric capacity of the polymers where the gravimetric capacity was calculated by measuring the charge during the discharging of the polymer electrode, accounting for the total mass of the polymer that has been drop casted onto the paper electrode). We note that the reported gravimetric capacities are only accounting for the mass loading of the polymer and do not account for the mass changes of the polymer electrodes due to swelling.

The theoretical capacity was calculated according to $C_{theoretical}[\frac{mAh}{g}] = \frac{nF*1000}{M*3600}$ with M being the moelcualr weight of one repeat unit and n = 2.

Spectroelectrochemical measurements:

The electrochemical cell was a quartz cuvette with transparent windows which enabled simultaneous optical spectra acquisition through a UV-vis spectrometer (OceanOptics USB 2000+) which collected the transmitted light through the sample from a tungsten lamp used as probe light source.[2] The electrochemical measurements were carried out with an IviumStat potentiostat, using a three electrode setup with a Ag/AgCl reference electrode (eDAQ leakless Ag/AgCl reference electrode) and a Pt-mesh as the counter electrode.

OECT measurements

OECT test chips were prepared following previously reported microfabrication techniques.[5] The OECT channels containing the p([g7:a]NDI-g3T2) series were fabricated by drop casting from chloroform (5 mg/mL) at room temperature, while the p([g7:a]NDI-T2) series were spin-coated. This was followed by patterning via peeling a sacrificial parylene layer and a rinse in deionized water. OECTs were gated with aqueous 0.1 M NaCl using an Ag/AgCl pellet as the

gate electrode. Electrical characterization (output, transfer, and pulsed stability) of the OECTs were carried out using NI source-measure units controlled by custom LabView code. μC^* was calculated from the slope of the transfer curves, which is the gate transconductance ($g_m = \partial I_d/\partial V_g$), using the relation $g_m = Wd/L \times \mu C^* \times (V_t - V_g)$.

Density functional theory calculations

All quantum chemical calculations (Density functional theory (DFT) and time-dependent DFT (TDDFT)) in this study were performed using Gaussian16.[6] p([g7:a]NDI-g3T2) and p([g7:a]NDI-T2) polymers were modelled with dimers of the respective polymers to simulate optical spectra of the neutral and negatively charged polymers while monomers were used for the electrostatic potential calculations. To save computational power, we replaced long side chains with short counterparts that preserved the main differences in chemical structure: the glycol side chain was represented as –(C_2H_4-O-CH_3), the alkyl side chain as –(C_4H_9) and alkoxy side chain on g3T2 as –(O-CH_3). Dimers and monomers in their neutral, polaron and bipolaron states were first optimized using DFT with the (B3LYP/6-31g(d,p)) functional / basis set combination. Excited-state TDDFT calculations were then performed on the optimized geometries of the neutral and reduced structures. The effect of aqueous environment was modelled by calculating optical spectra using the polarizable continuum model (PCM). Redox potentials in water were calculated using the solvation model based on density (SMD). Further computational details are included in the Supporting Information, section 9.

2 Synthesis and characterization

2.1 General synthesis

4,9-Dibromo-2,7-di(2,5,8,11,14,17,20-heptaoxadocosan-22-yl)benzo[lmn][3,8]phenanthroline-1,3,6,8(2H,7H)-tetraone (**g7-Br$_2$**)[1], N,N'-bis(2-octyldodecyl)-2,6-dibromonaphthalene -1,4,5,8-bis(dicarboximide) (**a-Br$_2$**)[9] and (3,3'-bis(2-(2-(2-methoxyethoxy)ethoxy)ethoxy)-[2,2'-bithiophene]-5,5'-diyl)bis(trimethylstannane) (**g3T2**)[2] were synthesized according to previously reported protocols. Polymers p([100:0]NDI-T2) and p([90:10]NDI-T2) were synthesized as previously reported.[1] Monomers (**g$_3$N**) and (**C$_{16}$N**) were synthesized as previously reported[10], the synthesis is summarised in Figure S4 ^{1}H-NMR spectra were recorded on 400 MHz Bruker spectrometers at ambient temperature. Chemical shifts are reported in units of parts per million (ppm, *δ*) for solutions in chloroform-*d* and coupling constants (*J*) are given in Hz. The chemical shifts were referenced using the residual chloroform peak as an internal standard (^{1}H NMR: 7.26 ppm). Number-average (M_n) and weight-average (M_w) molecular weights were determined using an Agilent Technologies 1260 infinity GPC at 40 °C in chloroform, using two PLgel 10 micrometer Mixed-B columns in series (300 × 7.5 mm), and calibrated against narrow dispersity ($Đ$ < 1.10) polystyrene standards.

2.2 Monomer and polymer synthesis

p([100:0]NDI-g3T2). A mixture of **g7-Br$_2$** (30.94 mg, 28.95 µmol), **g3T2** (23.63 mg, 28.95 µmol), tris(dibenzylideneacetone)-dipalladium(0) (0.53 mg, 0.58 µmol) and tri(*o*-tolyl)phosphine (0.70 mg, 2.30 µmol) in anhydrous, degassed chlorobenzene (1.5 mL) was heated to 135 °C for 16 h. A solution (0.1 mL) of 2-(tributylstannyl)thiophene (0.1 mL) and $Pd_2(dba)_3$ (1.05 mg, 1.15 µmol) in anhydrous, degassed chlorobenzene (0.5 mL) was added and the resulting mixture was stirred for 1 h at 135 °C. A solution (0.1 mL) of 2-bromothiophene (0.1 mL) in anhydrous, degassed chlorobenzene (0.5 mL) was subsequently added and stirring at 135 °C was maintained for a further 1 h. The reaction mixture was cooled to room temperature and precipitated in ethyl acetate, followed by addition of hexane. The solid was collected in a glass thimble and subjected to Soxhlet extractions with hexane, ethyl acetate, MeOH, acetone, THF and chloroform. After the final extraction with chloroform, the polymer solution was concentrated *in vacuo* and precipitated in ethyl acetate, followed by the addition of hexane. The polymer was collected by filtration and dried under reduced pressure to afford the product as a green solid in a yield of 53% (21.3 mg,

15.24 µmol). ^{1}H NMR (400 MHz, $CDCl_3$) δ 8.83 (br s, 2H), 7.31-7.17 (m, 2H), 4.44- 4.33 (m, 8H), 4.01-3.95 (m, 4H), 3.86-3.51 (m, 64H), 3.51-3.45 (m, 4H), 3.39-3.28 (m, 12H). GPC (chloroform, 40 °C): M_n = 6.6 kDa, M_w = 10.4 kDa.

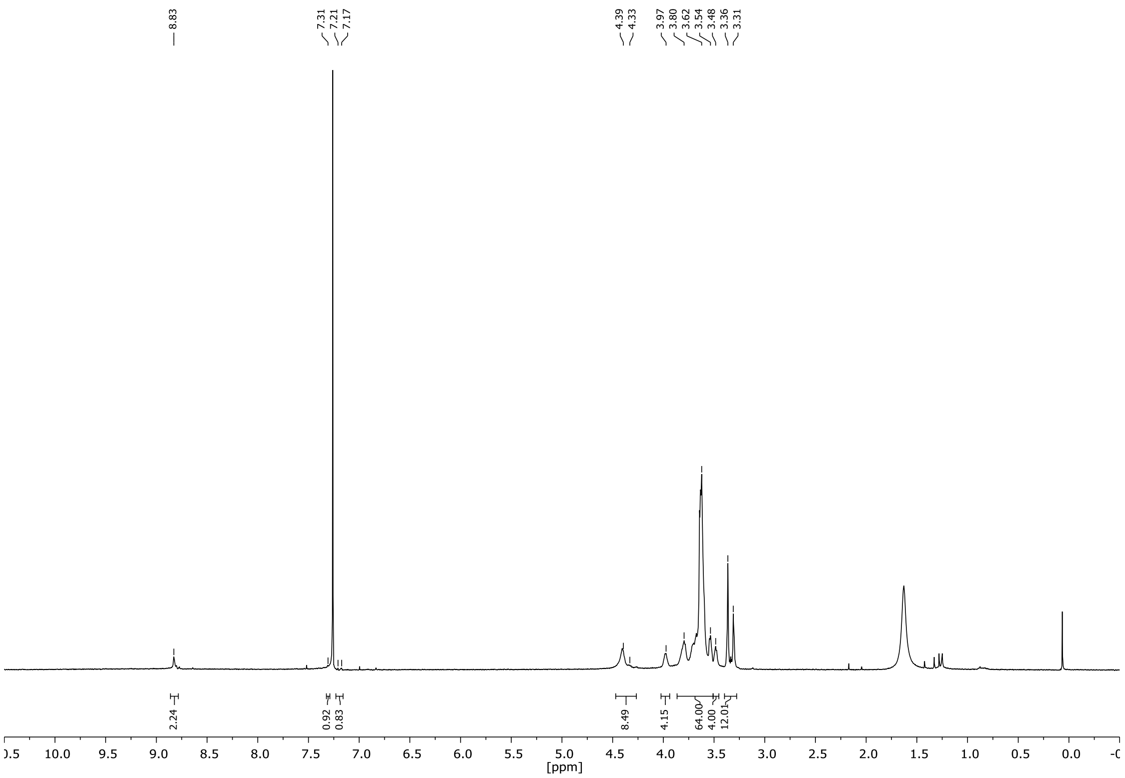

Figure S1. ^{1}H-NMR spectrum of p([100:0]NDI-g3T2) in $CDCl_3$.

p([90:10]NDI-g3T2). A mixture of **g7-Br$_2$** (27.33 mg, 25.57 µmol), **a-Br$_2$** (2.79 mg, 2.83 µmol), **g3T2** (23.19 mg, 28.41 µmol), tris(dibenzylideneacetone)-dipalladium(0) (0.52 mg, 0.57 µmol) and tri(*o*-tolyl)phosphine (0.69 mg, 2.27 µmol) in anhydrous, degassed chlorobenzene (1.5 mL) was heated to 135 °C for 16 h. A solution (0.1 mL) of 2-(tributylstannyl)thiophene (0.1 mL) and $Pd_2(dba)_3$ (1.06 mg, 1.16 µmol) in anhydrous, degassed chlorobenzene (0.5 mL) was added and the resulting mixture was stirred for 1 h at 135 °C. A solution (0.1 mL) of 2-bromothiophene (0.1 mL) in anhydrous, degassed chlorobenzene (0.5 mL) was subsequently added and stirring at 135 °C was maintained for a further 1 h. The reaction mixture was cooled to room temperature and precipitated in ethyl acetate, followed by addition of hexane. The solid was collected in a glass thimble and subjected to Soxhlet extractions with

hexane, ethyl acetate, MeOH, acetone, THF and chloroform. After the final extraction with chloroform, the polymer solution was concentrated *in vacuo* and precipitated in ethyl acetate, followed by the addition of hexane. The polymer was collected by filtration and dried under reduced pressure to afford the product as a green solid in a yield of 47% (18.6 mg, 13.39 µmol).^{1}H NMR (400 MHz, $CDCl_3$) δ 8.83 (br s, 2 H), 7.31-7.16 (m, 2 H), 4.49- 4.24 (m, 7.6 H), 4.11 (m, 0.4 H), 4.01-3.95 (m, 3.6 H), 3.89-3.51 (m, 59.6 H), 3.51-3.43 (m, 3.6 H), 3.40-3.27 (m, 11.4 H), 1.98 (br s, 0.2 H), 1.38-1.18 (m, 6.4 H), 0.84 (m, 1.2 H). GPC (chloroform, 40 °C): M_n = 13.6 kDa, M_w = 27.3 kDa.

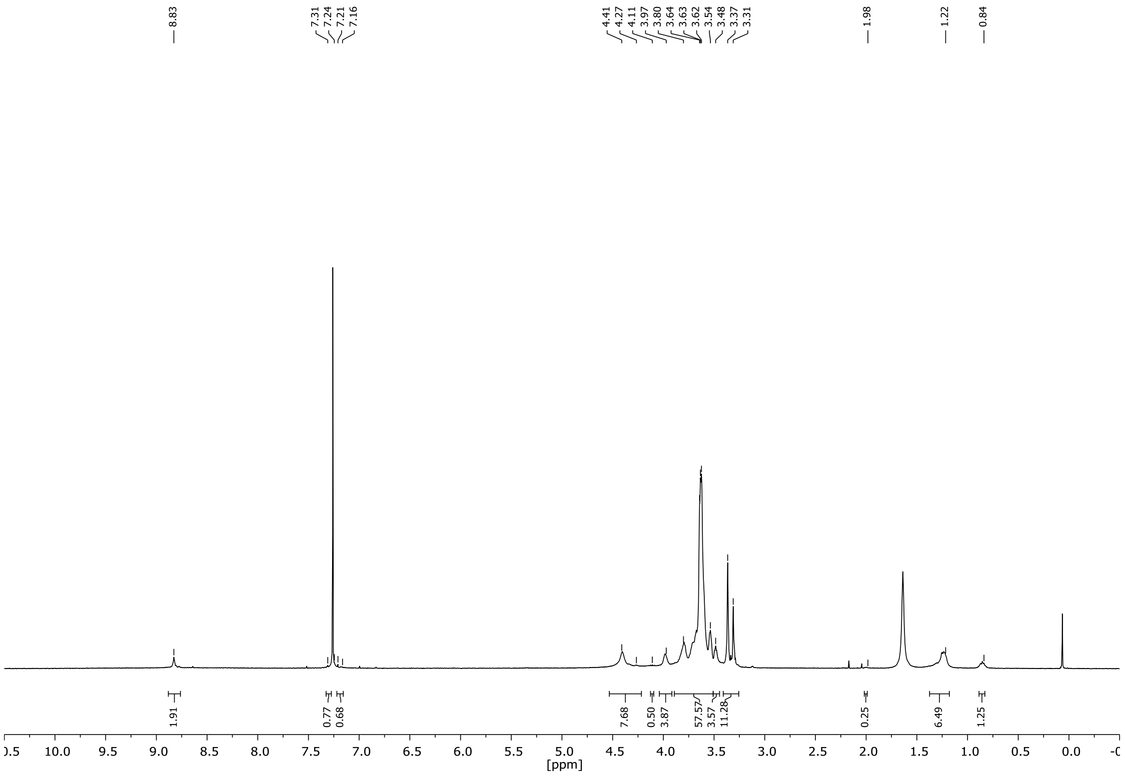

Figure S2. ^{1}H-NMR spectrum of p([90:10]NDI-g3T2) in $CDCl_3$.

p([75:25]NDI-g3T2). A mixture of **g7-Br$_2$** (44.53 mg, 41.66 µmol), **a-Br$_2$** (13.68 mg, 13.89 µmol), **g3T2** (45.35 mg, 55.56 µmol), tris(dibenzylideneacetone)-dipalladium(0) (1.02 mg, 1.11 µmol) and tri(*o*-tolyl)phosphine (1.35 mg, 4.44 µmol) in anhydrous, degassed chlorobenzene (1.5 mL) was heated to 135 °C for 16 h. A solution (0.1 mL) of 2-(tributylstannyl)thiophene (0.1 mL) and $Pd_2(dba)_3$ (1.14 mg, 1.24 µmol) in anhydrous, degassed chlorobenzene (0.5 mL) was added and the resulting mixture was stirred for 1 h at 135 °C. A solution (0.1 mL) of 2-bromothiophene (0.1 mL) in anhydrous, degassed chlorobenzene (0.5 mL) was subsequently added and stirring at 135 °C was maintained for a

further 1 h. The reaction mixture was cooled to room temperature and precipitated in ethyl acetate, followed by addition of hexane. The solid was collected in a glass thimble and subjected to Soxhlet extractions with hexane, ethyl acetate, MeOH, acetone, THF and chloroform. After the final extraction with chloroform, the polymer solution was concentrated *in vacuo* and precipitated in ethyl acetate, followed by the addition of hexane. The polymer was collected by filtration and dried under reduced pressure to afford the product as a green solid in a yield of 46% (35.2 mg, 25.57 µmol).^{1}H NMR (400 MHz, $CDCl_3$) δ 8.83 (br s, 2 H), 7.31-7.21 (m, 2 H), 4.45- 4.36 (m, 7 H), 4.11 (m, 1 H), 4.00-3.95 (m, 3 H), 3.89-3.52 (m, 53 H), 3.51-3.46 (m, 3 H), 3.40-3.29 (m, 8.5 H), 2.00 (br s, 0.5 H), 1.38-1.18 (m, 16 H), 0.86 (m, 3 H). GPC (chloroform, 40 °C): M_n = 12.5 kDa, M_w = 25.6 kDa.

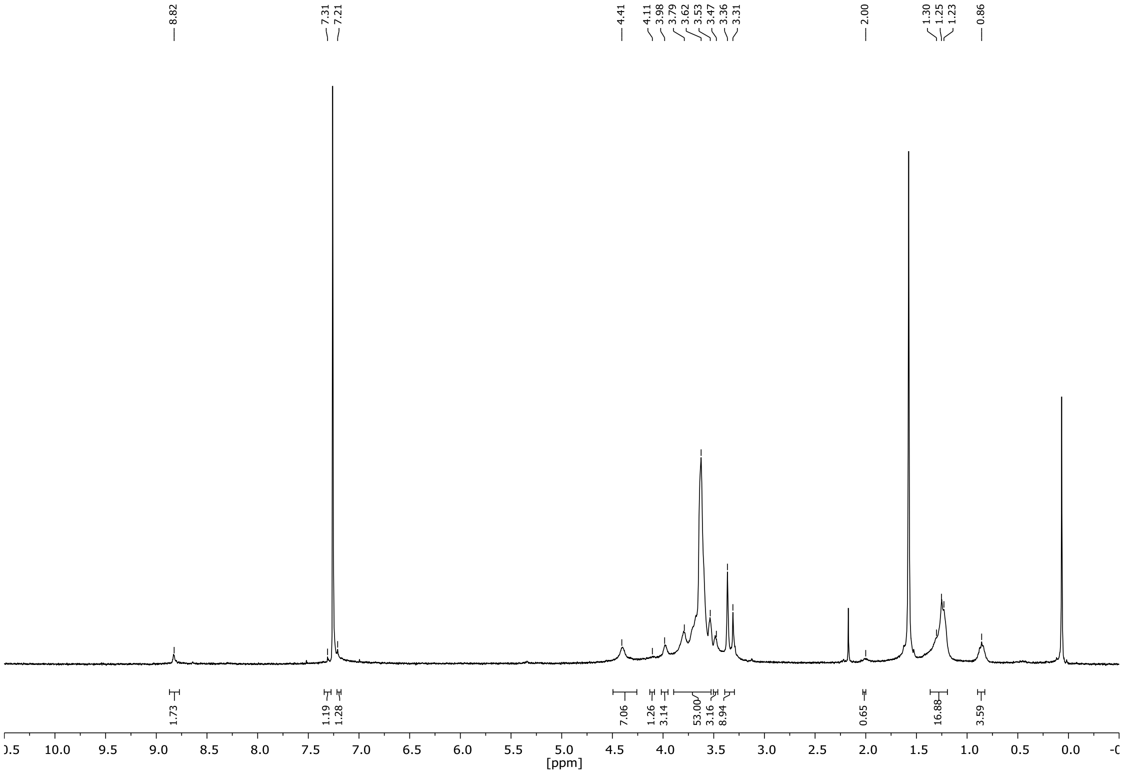

Figure S3. ^{1}H-NMR spectrum of p([75:25]NDI-g3T2) in $CDCl_3$.

2.3 Summary of the synthesis of monomer bg$_4$N and polymers p(bg$_4$NC$_{16}$N) and p(bg$_4$Ng$_3$N)

13-(2,5,8,11-tetraoxadodecyl)-2,5,8,11-tetraoxatetradecan-14-yl-4-methylbenzenesulfonate was synthesised according to previously reported protocols.[11] Biefly, a Finkelstein reaction, with sodium iodide in acetone afforded 13-(iodomethyl)-2,5,8,11,15,18,21,24-octaoxapentacosane. Next, the side chains were attached to 3,8-dihydroindolo[7,6-g]indole-1,2,6,7-tetraone[12], affording the monomer bg$_4$N via nucleophilic substitution in alkaline solution. Both C$_{16}$N and g$_3$N monomers were synthesized according to previously reported protocols[10] and were reacted with bg$_4$N to afford p(bg$_4$NC$_{16}$N) and p(bg$_4$Ng$_3$N), respectively.

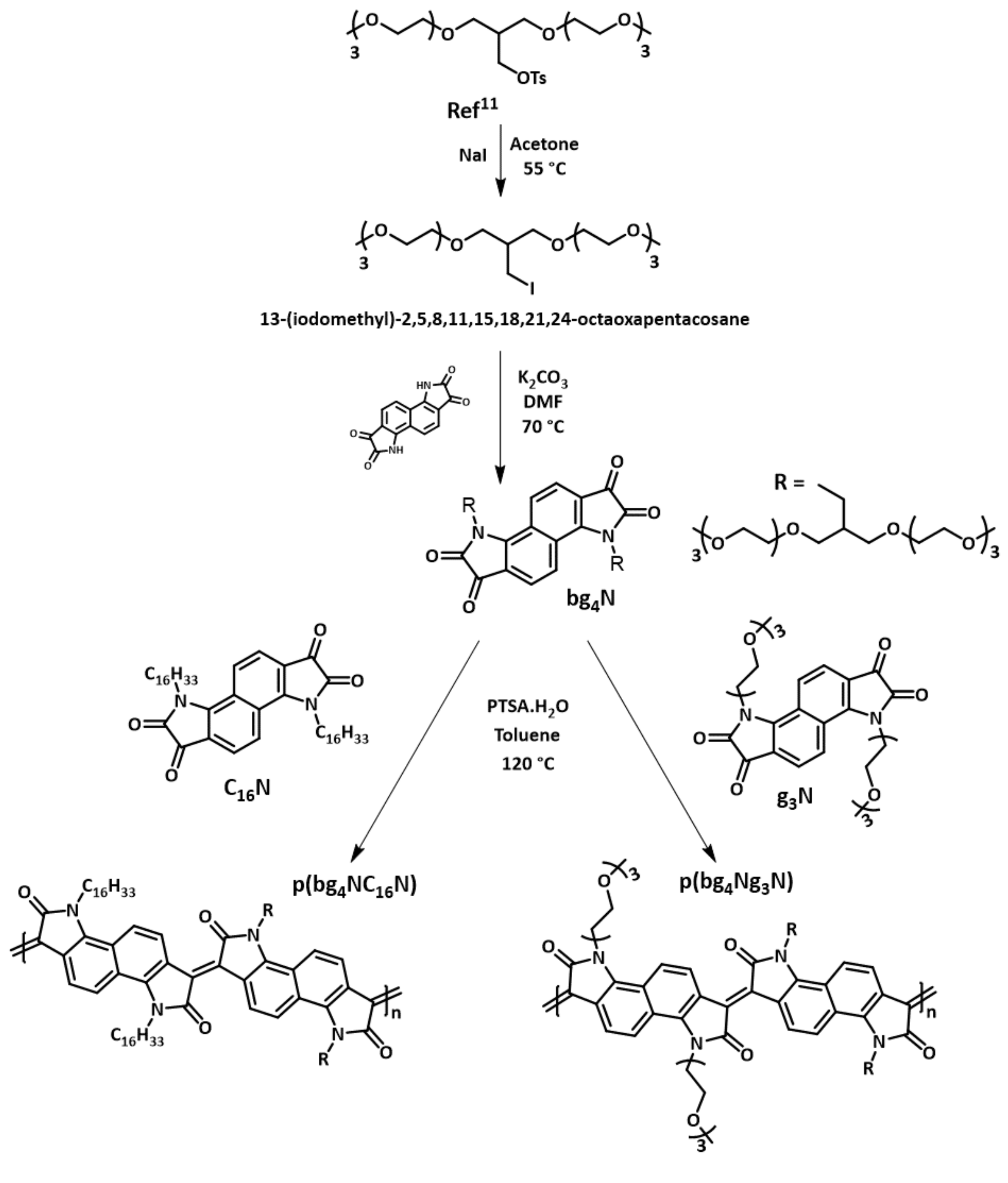

Figure S4. Reaction scheme for the synthesis of polymers p(bg$_4$NC$_{16}$N) and p(bg$_4$Ng$_3$N).

13-(iodomethyl)-2,5,8,11,15,18,21,24-octaoxapentacosane. A 2-neck oven dried 250 mL RBF was charged with NaI (4.88 g, 32.6 mmol, 2.0 eq.) and dispersed in acetone (100 mL) under a nitrogen atmosphere. 13-(2,5,8,11-tetraoxadodecyl)-2,5,8,11-tetraoxatetradecan-14-yl 4-methylbenzenesulfonate (which was synthesized according to previously reported protocols)[11] (9 g, 16.3 mmol, 1.0 eq.) was added dropwise via a syringe. The reaction was heated to 55 °C and stirred overnight. Upon cooling to room temperature, the mixture was poured into water, washed with brine, and extracted with DCM. The organic layer was separated, dried over $MgSO_4$ and the solvent was removed under reduced pressure to yield a crude pale-yellow oil. The crude was purified by column chromatography on silica using a solvent mixture of ethyl acetate:methanol (9:1) as the eluent to afford the title compound as a colorless viscous oil (7.9 g, 95%).

^{1}H NMR (400 MHz, $CDCl_3$) δ 3.68 – 3.57 (m, 20H), 3.61 – 3.45 (m, 8H), 3.38 (s, 6H), 3.36 (d, *J* = 5.6 Hz, 2H), 1.93 (qd, *J* = 6.7, 5.3 Hz, 1H). ^{13}C NMR (101 MHz, CDCl3) δ 72.07, 71.53, 70.78, 70.75, 70.69, 70.60, 59.20, 40.83, 8.48.

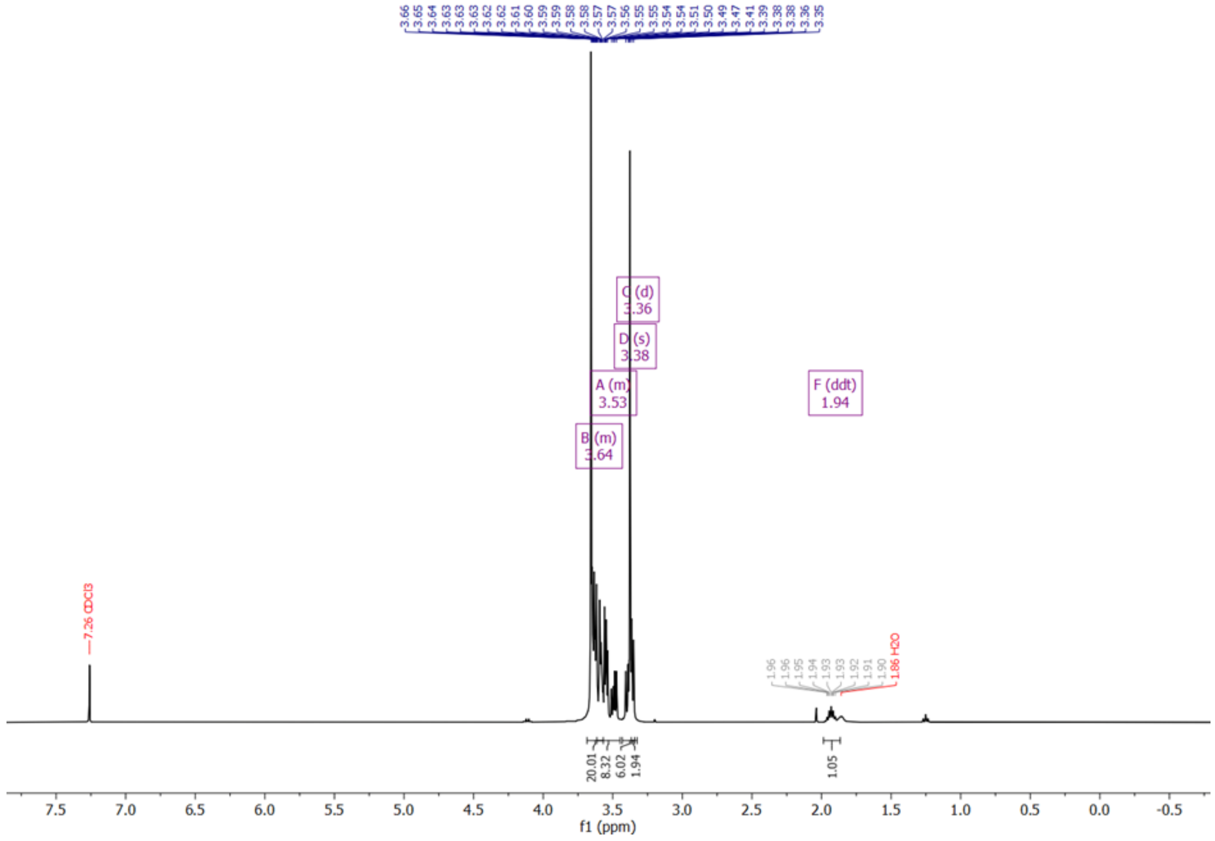

Figure S5. ^{1}H NMR spectrum of 13-(iodomethyl)-2,5,8,11,15,18,21,24-octaoxapentacosane.

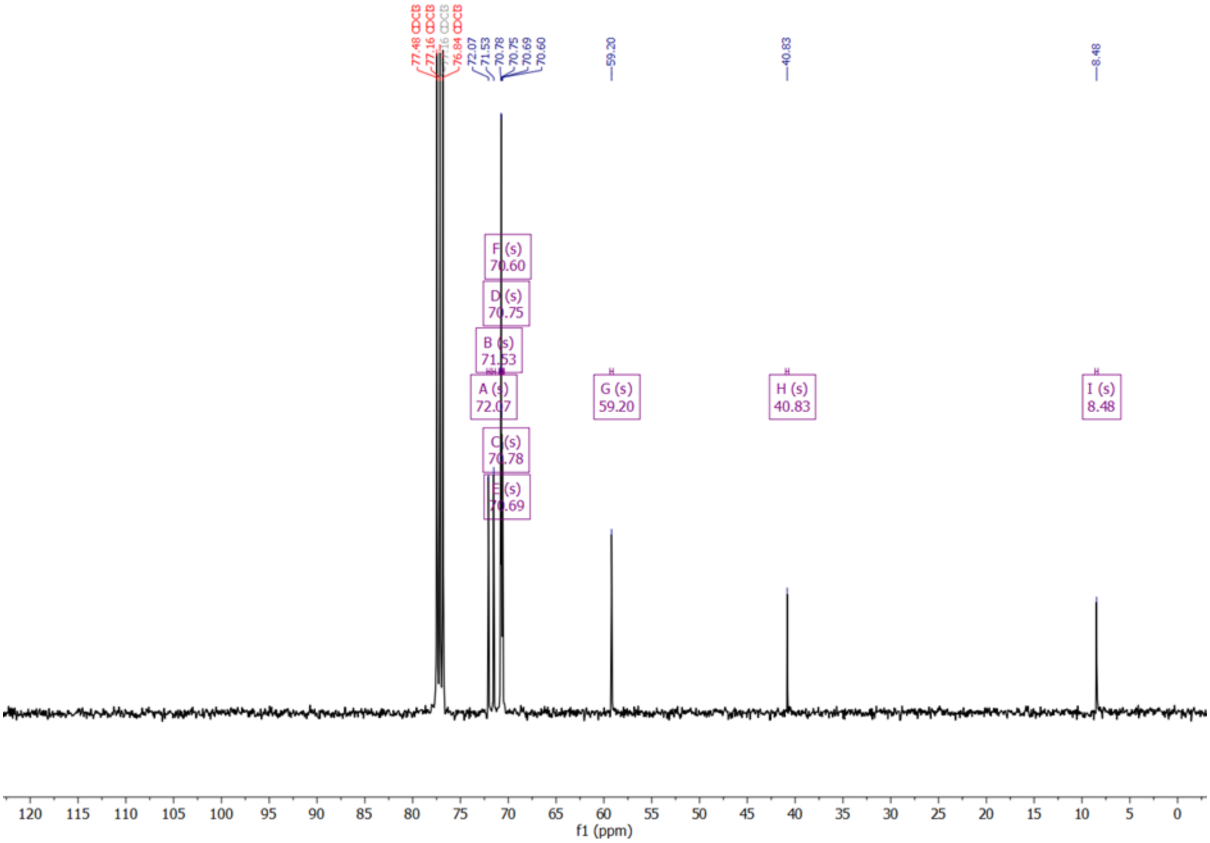

Figure S6. ^{13}C NMR spectrum of 13-(iodomethyl)-2,5,8,11,15,18,21,24-octaoxapentacosane.

(**bg$_4$N).** Oven dried potassium carbonate (1.75 g, 12.66 mmol, 5.0 eq.) and 3,8-dihydroindolo[7,6-g]indole-1,2,6,7-tetraone (674 mg, 2.53 mmol, 1.0 eq.) was dissolved in 10 mL of dry DMF inside a 2-neck 50 mL RBF, under a Nitrogen atmosphere. The mixture was heated to 70 °C for one hour. 13-(iodomethyl)-2,5,8,11,15,18,21,24-octaoxapentacosane (5.6 g, 10.13 mmol, 4.0 eq.) was injected into the reaction in a single portion and the mixture was left to stir for an additional 4 hours. Upon cooling to room temperature, the reaction was poured into water and acidified to pH using aqueous 2M HCl. A saturated ammonium chloride solution was added to help separate the organic layer upon the addition of DCM. Once separated the organic phase was dried over $MgSO_4$ and solvent removed to yield a deep purple sticky solid. The crude product was columned on silica twice with a DCM:Acteone (9:1) eluent system and a third time using DCM:Acetone (8:2) to isolate the desired blue product. The title compound was isolated as a blue solid (347 mg, 13%).

^{1}H NMR (400 MHz, $CDCl_3$) δ 8.43 (d, *J* = 8.8 Hz, 2H), 7.64 (d, *J* = 8.7 Hz, 2H), 4.41 (d, *J* = 6.8 Hz, 4H), 3.67 – 3.54 (m, 38H), 3.58 – 3.49 (m, 14H), 3.47 (dd, *J* = 9.6, 7.4 Hz, 4H), 3.35 (s, 12H), 2.52 (s, 2H). ^{13}C NMR (101 MHz, $CDCl_3$) δ 183.23, 159.89, 152.25, 127.56, 121.52, 120.24,

116.46, 72.07, 70.95, 70.88, 70.76, 70.67, 59.16, 43.36, 40.24. Mass (MALDI-ToF): 531.2 $[M+H]^+$ (calc. 530.3 $C_{50}H_{78}N_2O_{20}$).

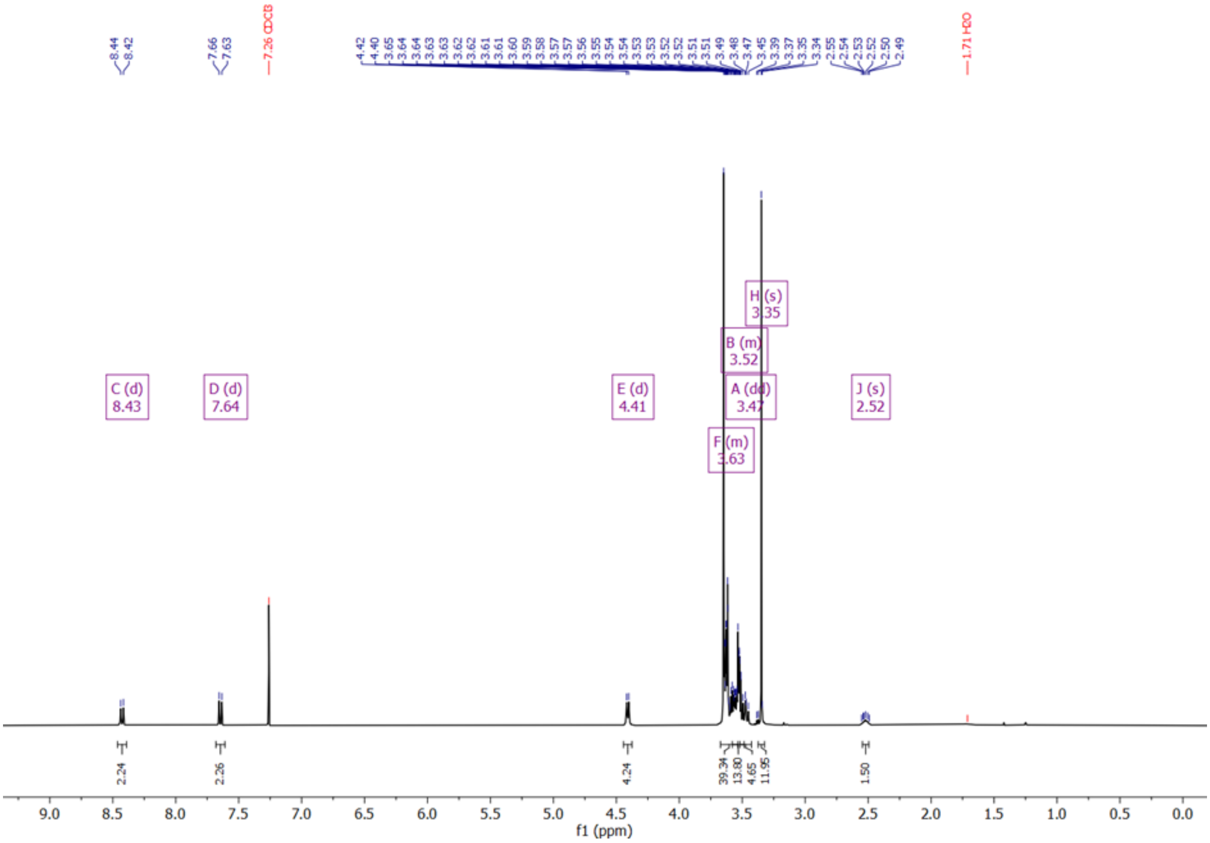

Figure S7. ^{1}H-NMR spectrum of bg_4N in $CDCl_3$.

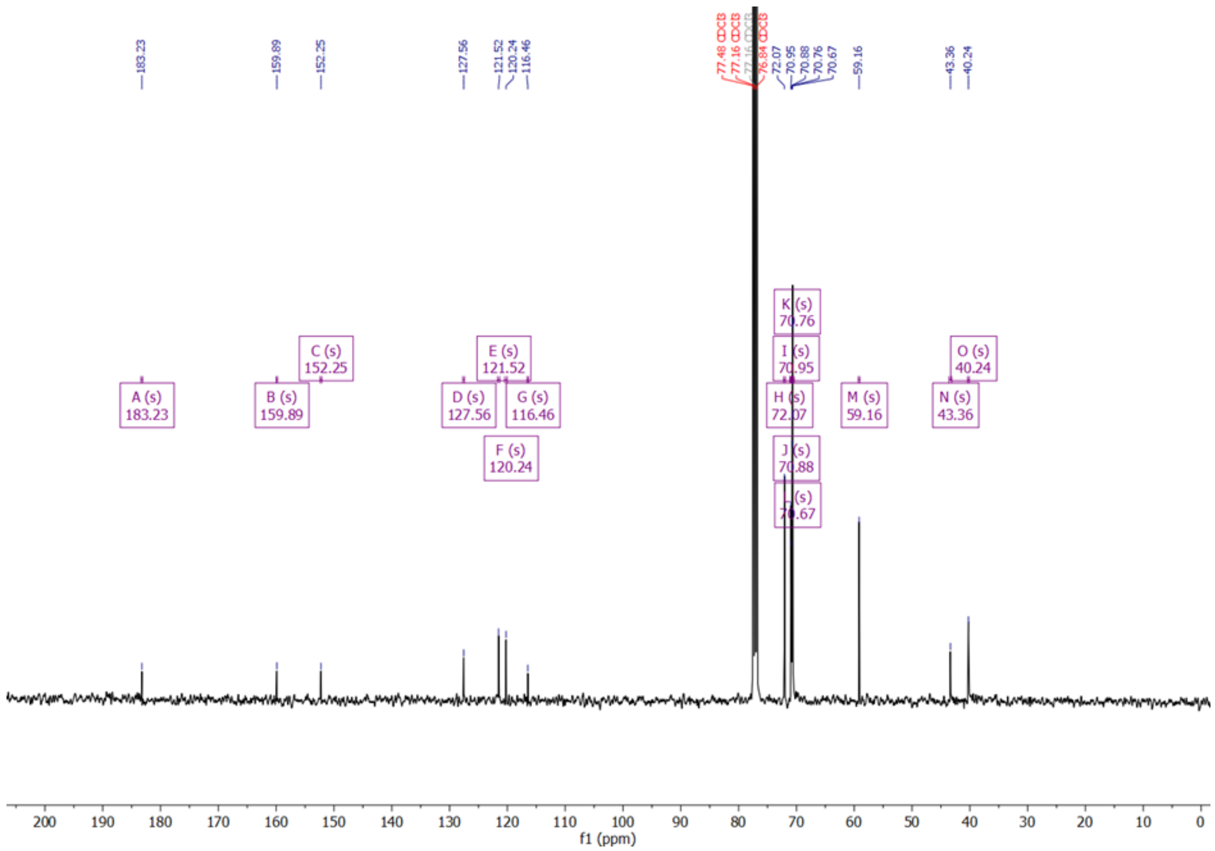

Figure S8. ^{13}C NMR spectrum of bg$_4$N in $CDCl_3$.

p(bg$_4$Ng$_3$N). An oven dried 5 mL microwave vial was charged with **bg$_4$N** (84 mg, 0.08 mmol, 1.0 eq.), **g$_3$N** (43.4 mg, 0.08 mmol, 1.0 eq.) and *p*-toluenesulfonic acid monohydrate (4.7 mg, 0.025 mmol, 0.3 eq.). The cap was sealed and the vial was degassed with nitrogen for 10 minutes prior to the addition of 1 mL anhydrous toluene. The reaction mixture was stirred for 48 hours at 120 °C. A color change to deep purple was observed and the reaction mixture formed a gel upon cooling to room temperature. The crude polymer was precipitated into 100 mL of methanol and subsequently filtered into a Soxhlet thimble. Purification via Soxhlet extraction was carried out with hexane, methanol, toluene, acetone and chloroform (in that order). The main fraction of the polymer was dissolved in chloroform. The solvent was removed under reduced pressure and the product was precipitated into methanol and filtered to yield a purple solid. (84 mg, 66%). GPC (chloroform, 40 °C): M_n = 17.2 kDa, M_w = 64.5 kDa.

^{1}H NMR (500 MHz, $CDCl_3$) δ 9.23 – 8.68 (m, 4H), 8.43 – 7.55 (m, 4H), 4.67 – 4.34 (m, 4H), 4.23 – 2.94 (m, 102H), 2.78 – 2.32 (m, 2H).

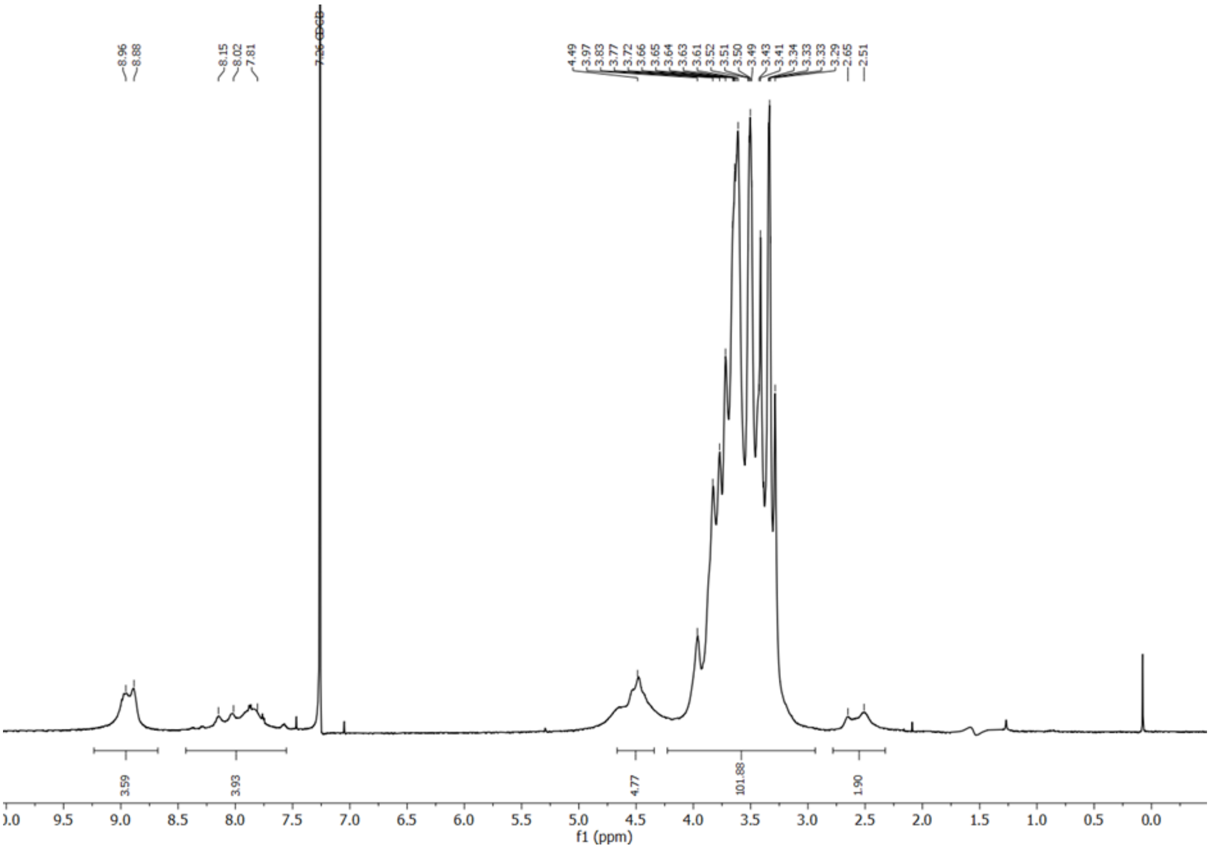

Figure S9. ^{1}H-NMR spectrum of p(bg$_4$Ng$_3$N) in $CDCl_3$, measured at 45 °C.

p(bg$_4$NC$_{16}$N). An oven dried 5 mL microwave vial was charged with **bg$_4$N** (92 mg, 0.09 mmol, 1.0 eq.), **C$_{16}$N** (61.5 mg, 0.09 mmol, 1.0 eq.) and *p*-toluenesulfonic acid monohydrate (5.11 mg, 0.03 mmol, 0.3 eq.). The cap was sealed and the vial was degassed with nitrogen for 10 minutes prior to the addition of 1 mL anhydrous toluene. The reaction mixture was stirred overnight at 120 °C. A color change to deep purple was observed and the reaction mixture formed a gel upon cooling to room temperature. The crude polymer was precipitated into 100 mL of methanol and subsequently filtered into a Soxhlet thimble. Purification via Soxhlet extraction was carried out with hexane, methanol, toluene, acetone and chloroform (in that order). The main fraction of the polymer was dissolved in chloroform. The solvent was removed under reduced pressure and the product was precipitated into methanol and filtered to yield a purple solid. (99 mg, 65%). GPC (chloroform, 40 °C): M_n = 13.5 kDa, M_w = 20.8 kDa.

^{1}H NMR (500 MHz, $CDCl_3$) δ 9.09 – 8.65 (m, 4H), 8.16 – 7.33 (m, 4H), 4.54 – 4.10 (m, 4H), 3.93 – 3.12 (m, 68H), 2.01 – 1.72 (m, 4H), 1.70 – 1.04 (m, 56H), 0.95 – 0.81 (s, 6H).

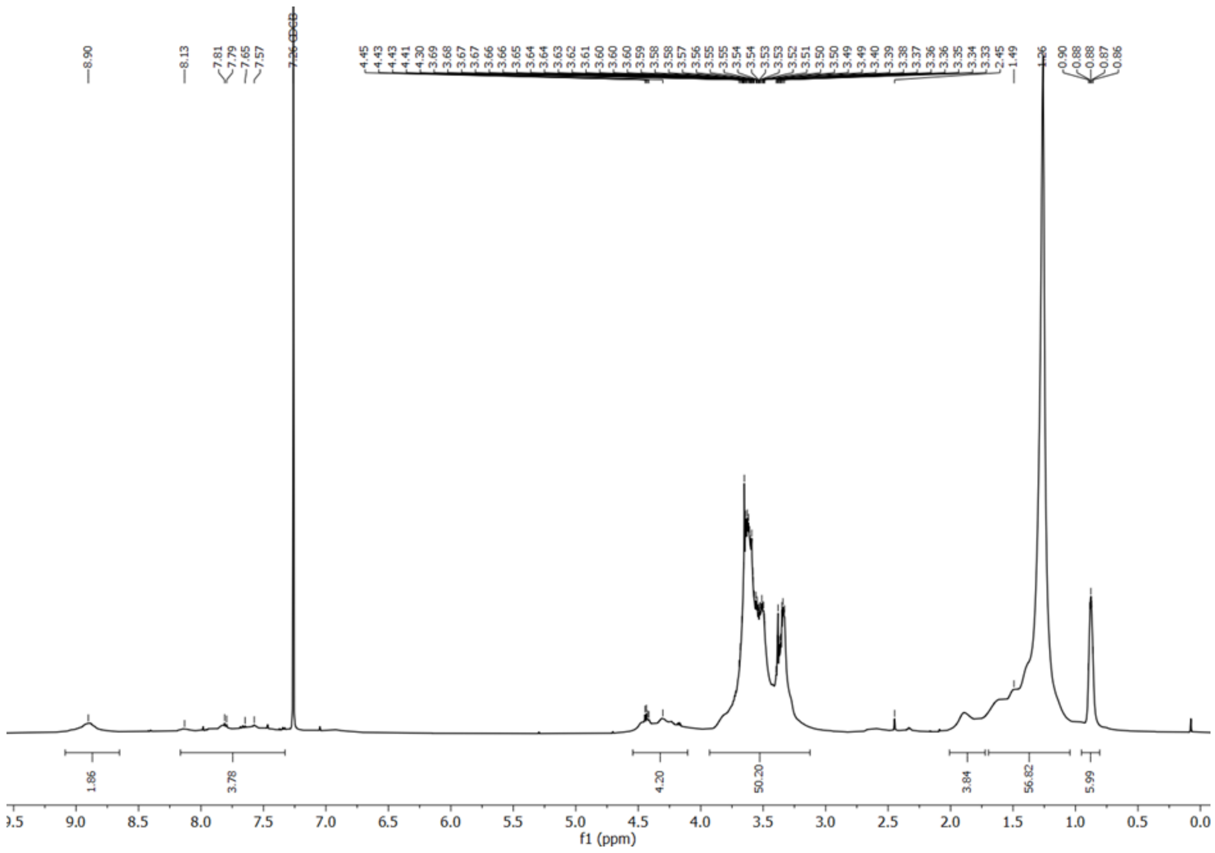

Figure S10. ^{1}H-NMR spectrum of p(bg$_4$NC$_{16}$N) in $CDCl_3$, measured at 45 °C.

3 GPC measurements

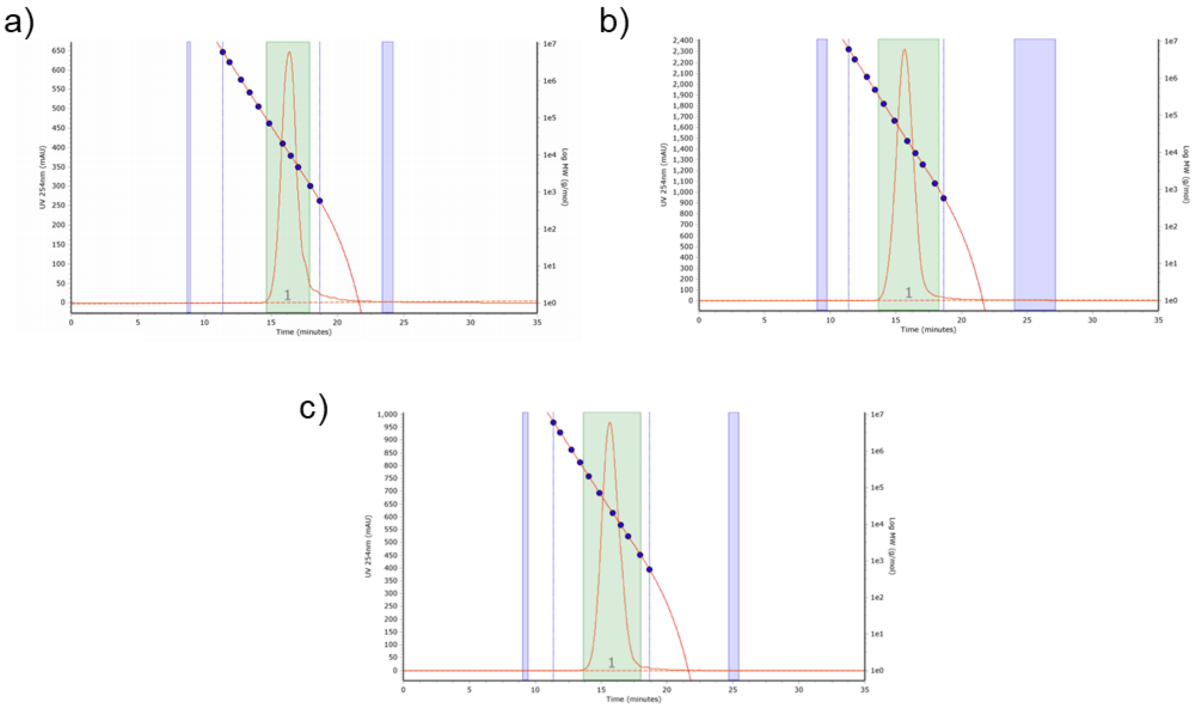

Figure S11. GPC traces of a) p([100:0]NDI-g3T2), b) p([90:10]NDI-g3T2) and c) p([75:25]NDI-g3T2) measured in chloroform at 40 °C.

4 Optical measurements

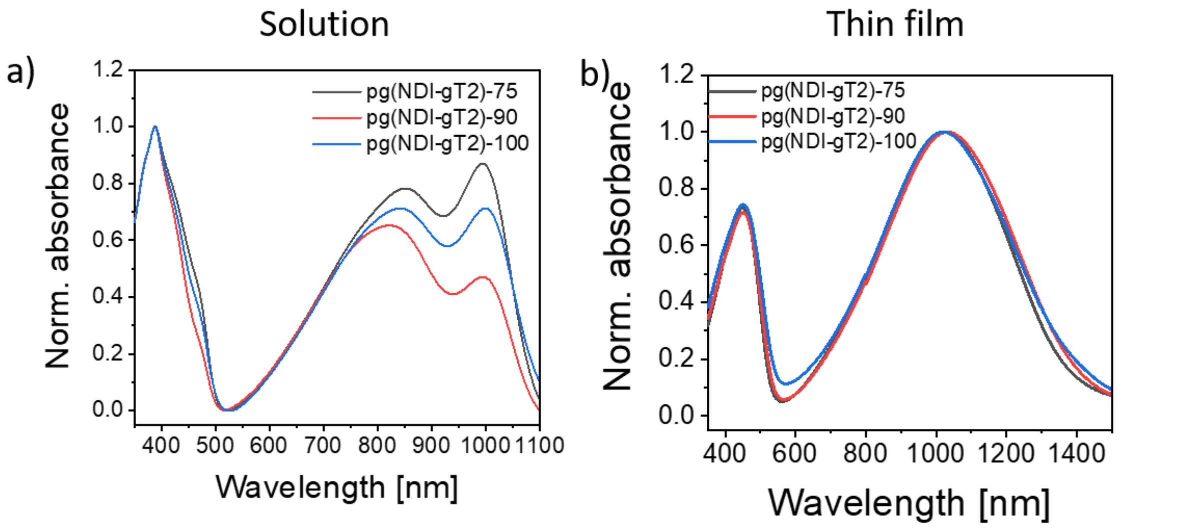

Figure S12. UV Vis-absorption spectrum of the polymer p([75:25]NDI-g3T2), p([90:10]NDI-g3T2), p([100:0]NDI-g3T2) a) in solution (chloroform) and b) as thin films on glass substrates.

5 Swelling analysis

5.1 AFM analysis

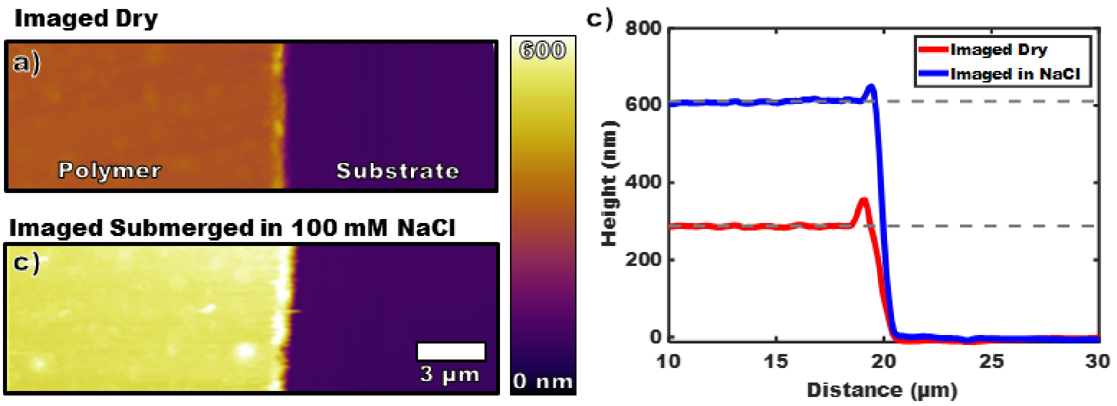

Figure S13. Illustration of the Atomic Force Microscopy (AFM) thickness measurement on dry and hydrated films. a) Image over a scratch measured in N_2 flow. b) Image over a scratch while under degassed 100 mM NaCl aqueous solution. c) Representative line profiles extracted from images a, b showing significant expansion of the polymer in contact with electrolyte.

5.1.1 Summary of AFM measurements

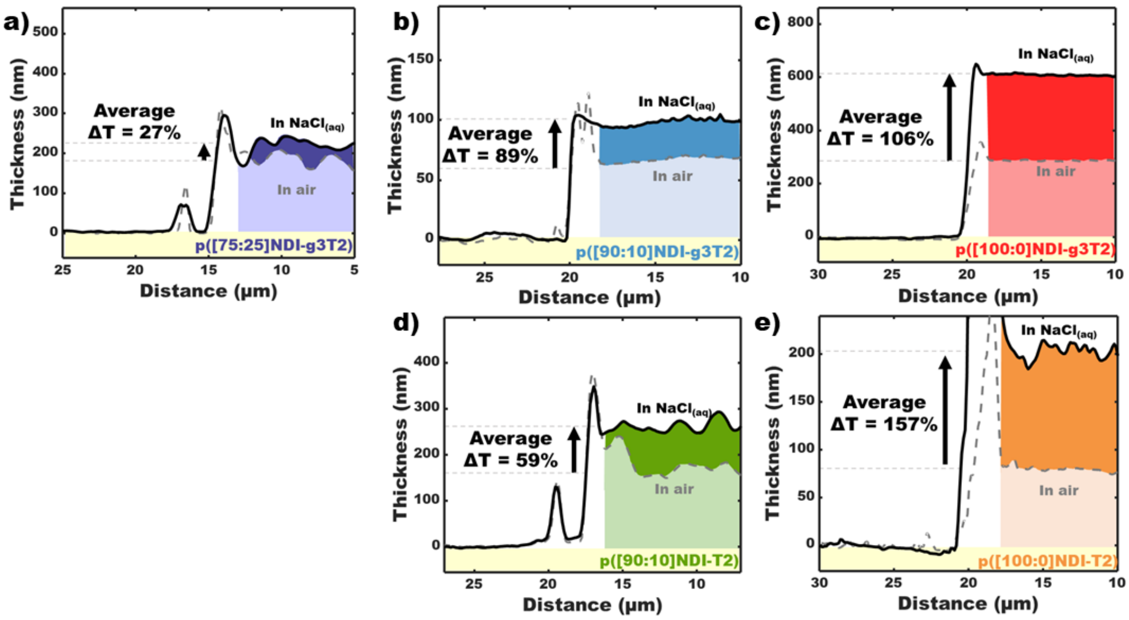

Figure S14. Plots show representative line profiles used to calculate the thickness in both N_2 (dashed gray lines) and in 100 mM $NaCl_{(aq.)}$ (solid black lines). The noted percent increase in thickness when immersed in electrolyte is the averaged from at least 5 separate line profiles and the error is the standard devation of these line profiles. **a)** p([75:25]NDI-g3T2) shows an average swelling of 27 % ± 11 %, **b)** p([90:10]NDI-g3T2) has an average swelling of 89 % ± 31 %, **c)** p([100:0]NDI-g3T2) shows an average swelling of 106 % ± 48%, **d)** p([90:10]NDI-T2) shows an average swelling of 59 % ±7 %, and **e)** p([100:0]NDI-T2) shows an average swelling of 157 % ± 46 %.

5.1.2 Redrying of the p([g7:a]NDI-g3T2) series

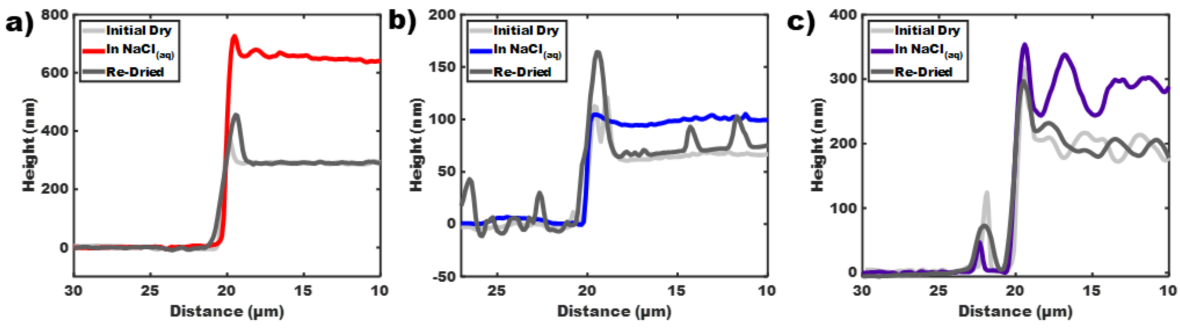

Figure S15. Redrying of the p([g7:a]NDI-g3T2) polymer films. Line profiles taken of the initial dry film (grey), the $NaCl_{(aq)}$ swollen films, and after extended drying in air showing that the films return to their original dry thickness. a) p([100:0]NDI-g3T2), b) p([90:10]NDI-g3T2) and c) p([75:25]NDI-g3T2).

5.2 QCM-D analysis

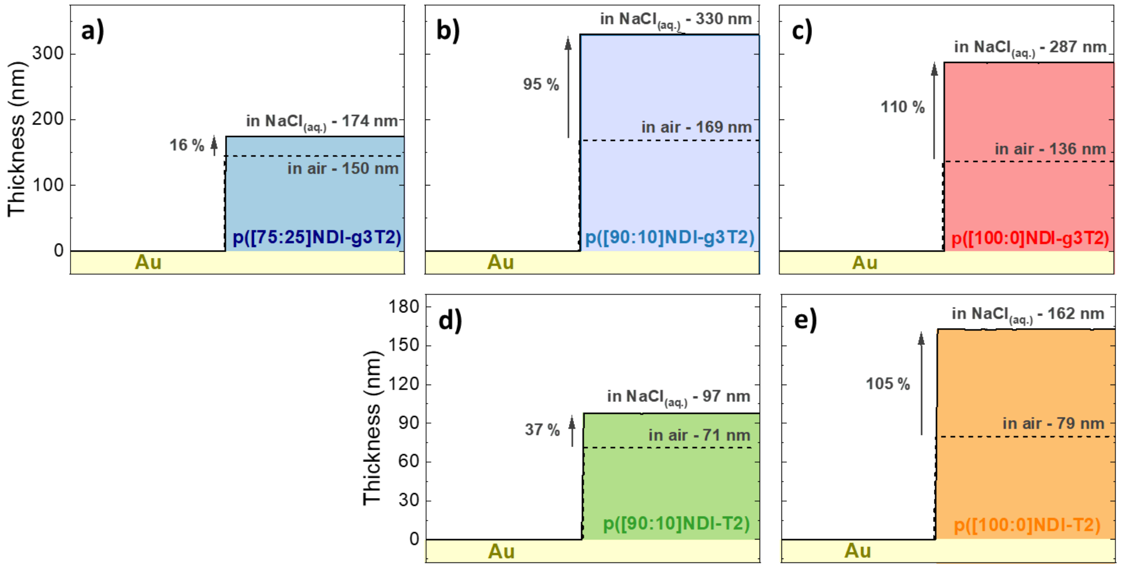

Figure S16. Swelling of the polymers under study calculated with QCM-D measurements. The thickness is calculated based on the 5th overtone of the raw data (not shown), in both air (dashed lines) and in 0.1 M NaCl aqueous solution (solid lines). The percentage of increase in thickness when each polymer is immersed in 0.1 M NaCl aqueous solution (i.e., swelling) is noted on each graph. a) p([75:25]NDI-g3T2), b) p([90:10]NDI-g3T2), c) p([100:0]NDI-g3T2), d) p([90:10]NDI-T2) and e) p([100:0]NDI-T2).

6 Electrochemical and spectroelectrochemical measurements

6.1 CV measurements in organic electrolytes

The electron affinity (EA) was calculating by:

$EA = IP(Fc/Fc^+) - E_{1/2}(Fc/Fc^+ \text{ vs. Ag/AgCl}) + E_{Red,onset}$ (Polymers)

$EA = 4.8 \text{ eV} - 0.38 \text{ V} + E_{Red,onset}$ (Polymers)

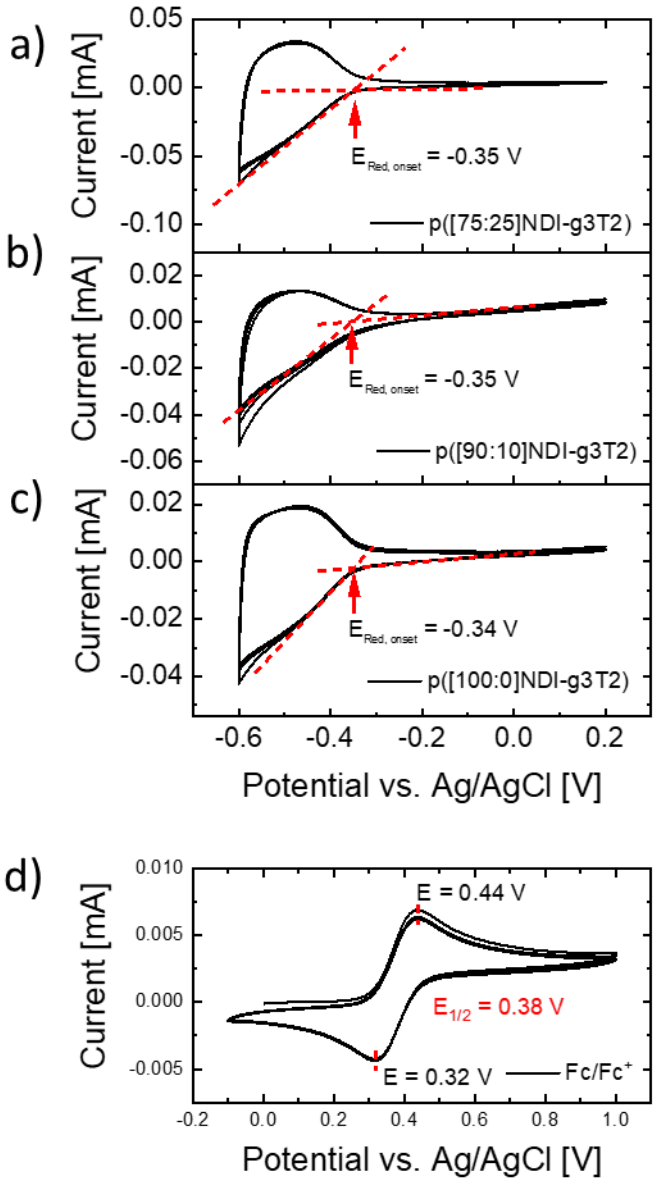

Figure S17. CV measurements in 0.1 M NBu_4PF_6 with a scan rate of 100 mV/s shown three scans of a) p([75:25]NDI-g3T2) [EA = 4.1 eV], b) p([90:10]NDI-g3T2) [EA = 4.1], c) p([90:10]NDI-g3T2) [EA = 4.1] and d) Ferrocene.

6.2 CV measurements in aqueous electrolytes

6.2.1 Thin films on ITO coated glass substrates

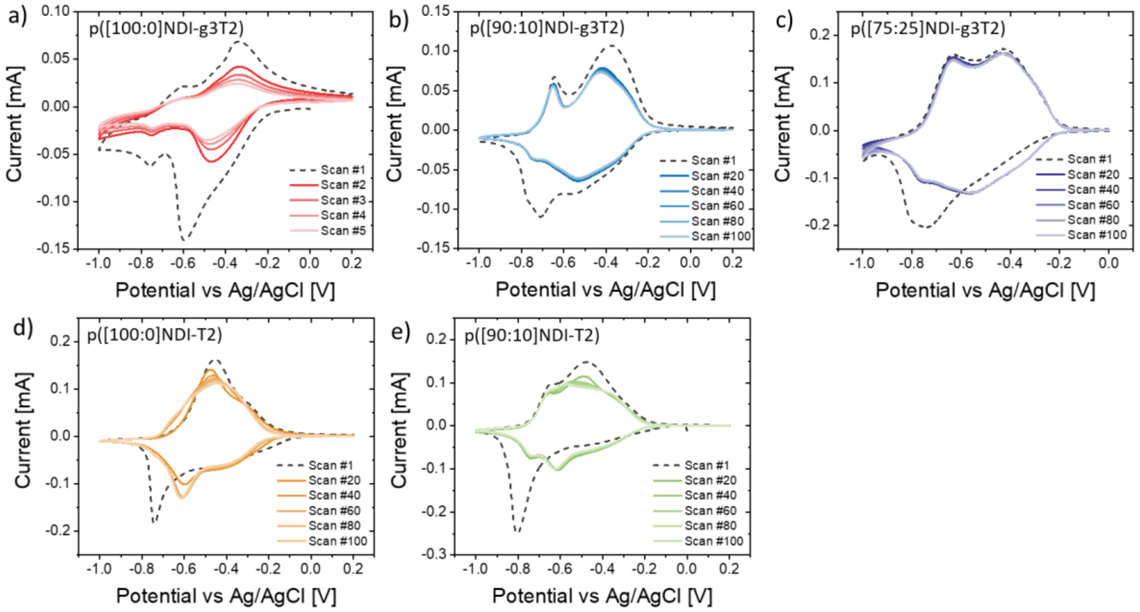

Figure S18. CV measurements of polymer thin films on ITO coated glass substrates in 0.1 M NaCl aqueous electrolytes with a scan rate of 50 mV/s at low O_2 concentration, showing a) p([100:0]NDI-g3T2), b) p([90:10]NDI-g3T2), c) p(75:25]NDI-g3T2), d) p([100:0]NDI-T2) and e) p([90:10]NDI-T2). The first cycle is highlighted (dashed line) and every 20th cycle is shown to compare the redox-stability of the polymers. Note: The polymer p([100:0]NDI-g3T2) shows a poor redox-stability, probably due to delamination of the polymer film after 5 cycles.

6.2.2 Thick electrodes on conductive paper electrodes

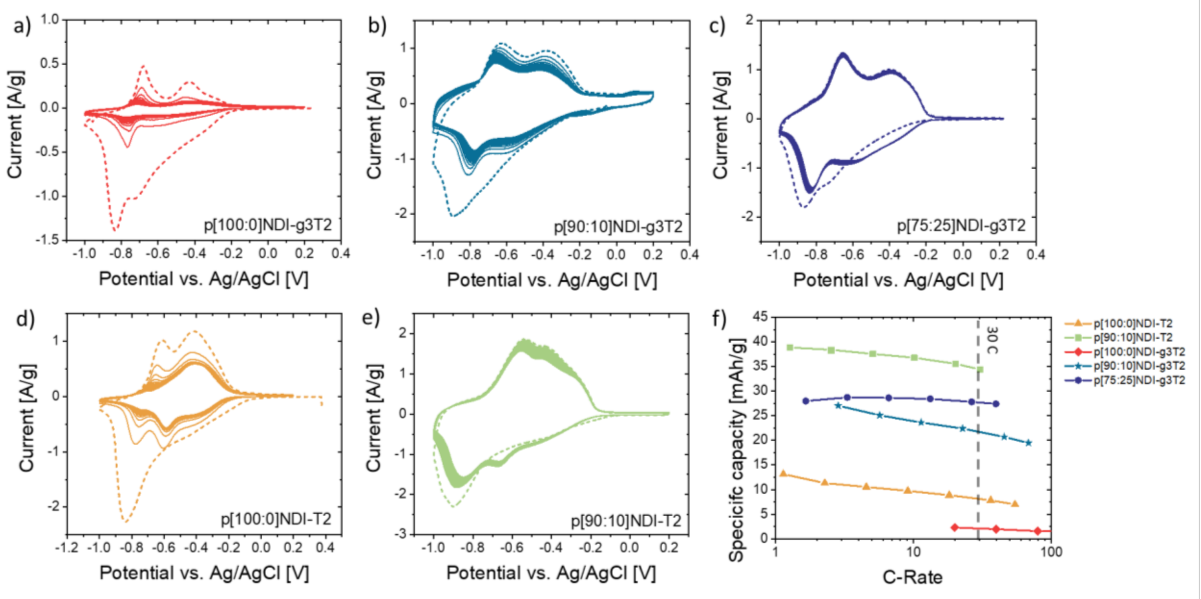

Figure S19. CV measurements of polymer electrodes on conductive paper, charging the electrode between 0.2 V to –1.0 V vs. Ag/AgCl for 100 scans with a scan rate of 5 mV/s s for a) p([100:0]NDI-g3T2) (2.22 mg), b) p([90:10]NDI-g3T2) (1.30 mg), c) p([75:25]NDI-g3T2) (2.16 mg), d) p([100:0]NDI-T2) (1.7 mg) and e) p([90:10]NDI-T2) (2.03 mg) in 0.1 M NaCl solution with low oxygen concentrations. The first cycle of the measurement is highlighted (dashed line). C-rate analysis of the polymer electrodes employing chronopotentiometry (CP) measurements between 0.2 V and –1.0 V vs. Ag/AgCl.

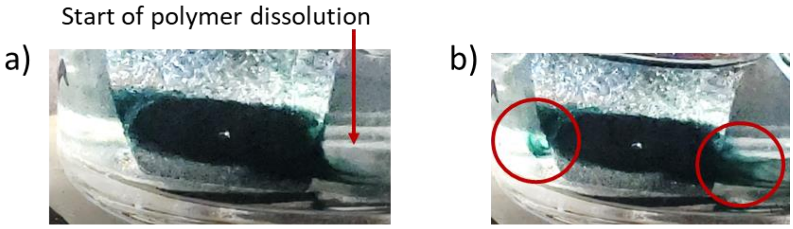

Figure S20. Monitoring of the charging of a polymer electrode with p([100:0]NDI-g3T2) and sign of dissolution of the polymer when charged to the electron bipolaron.

6.3 Performance of other conjugated polymers

6.4 Evaluating the concept across other polymer backbones

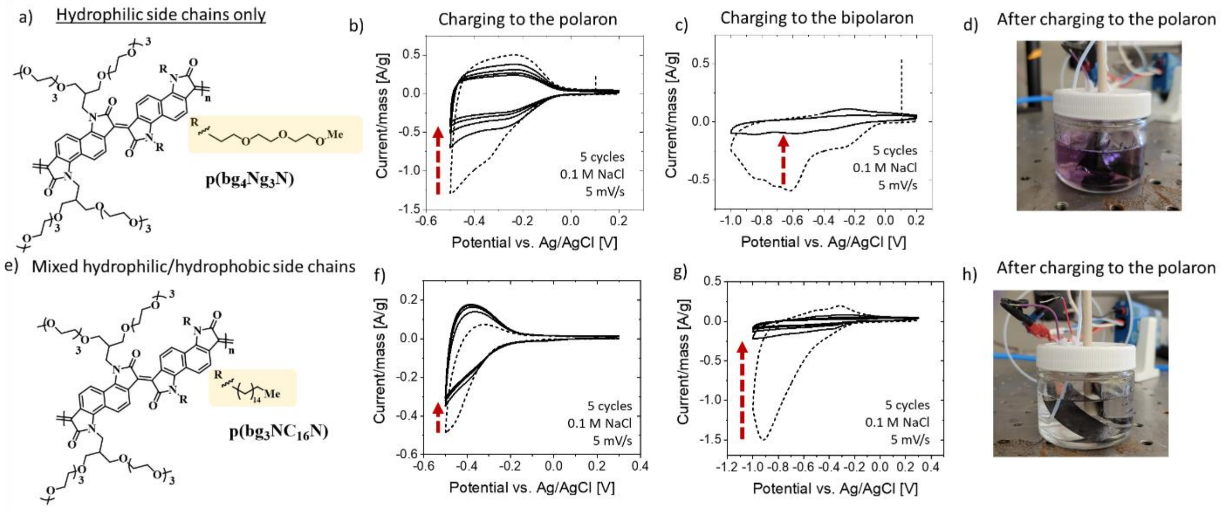

Figure S21: Electrochemical performance of other n-type polymers. a) Chemical structure of p(bg$_4$Ng$_3$N) with hydrophilic side chains only, CV measurements of p(bg$_4$Ng$_3$N) in 0.1 M NaCl at a scan rate of 5 mV/s to b) -0.5 V (electron polaron) vs. Ag/AgCl and c) -1.0 V vs. Ag/AgCl (electron bipolaron), d) picture of the electrolyte after charging to the electron polaron, the coloring of the electrolyte is a result of the polymer dissolution during the charging, e) chemical structure of p(bg$_3$NC$_{16}$N) with mixed hydrophilic and hydrophobic side chains. CV measurements of p(bg$_3$NC$_{16}$N) in 0.1 M NaCl at a scan rate of 5 mV/s to f) -0.5 V vs. Ag/AgCl (electron polaron) and g) -1.0 V vs. Ag/AgCl (electron bipolaron) and h) picture of the electrolyte after charging to the polaron, showing no coloring due to the low-solubility of the charged polymer in the aqueous electrolyte.

6.4.1 Poly(benzimidazobenzophenanthroline) (BBL)

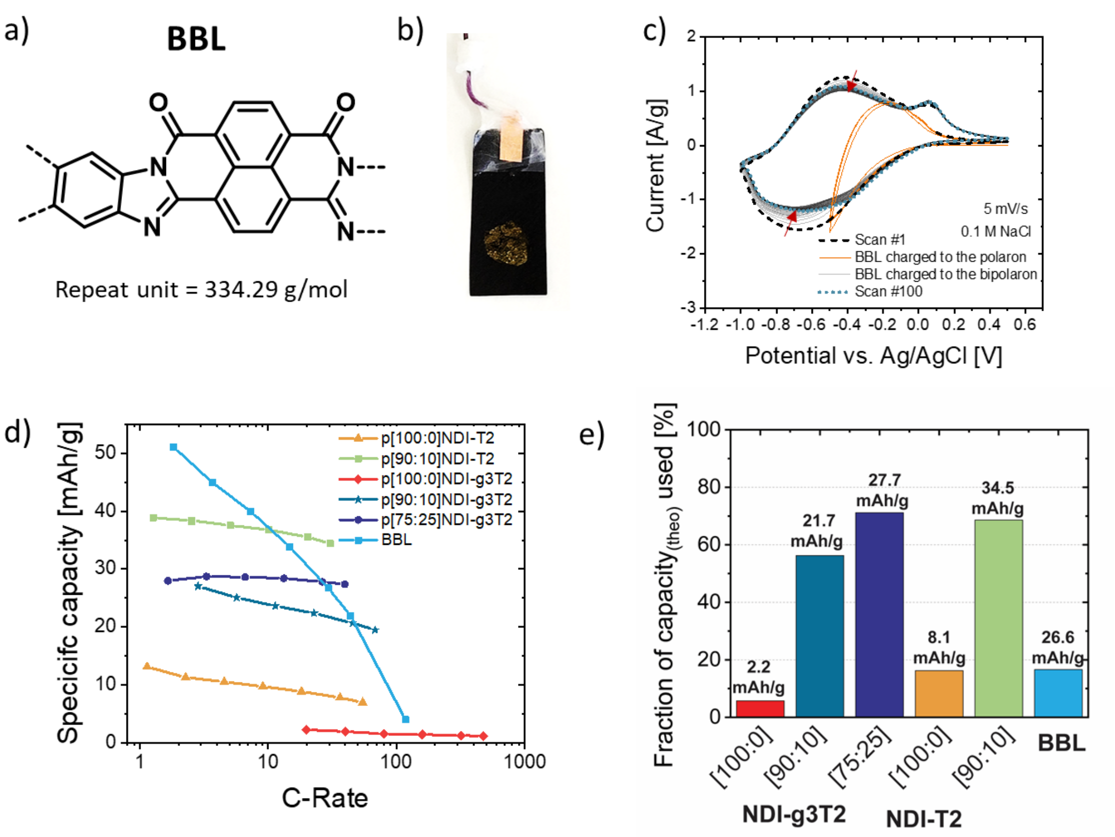

Figure S22. Electrochemical properties of a single-phase electrode with BBL. a) Chemical structure of one repeat unit of BBL, b) Paper electrode with BBL (single-phase electrode, mass loading of 1.04 mg), c) CV measurements of the BBL polymer electrode with a scan rate of 5 mV/s in 0.1 M NaCl with low O_2 concentration when charging the electrode to between – 0.5 V to +0.5 V vs. Ag/AgCl (electron polaron, orange line, three cycles)) and –1.0 V to 0.5 V vs. Ag/AgCl (electron bipolaron, 100 scans, black to gray). Scan #1 (black dashed line) and scan #100 (blue dotted line) are highlighted and red arrows are added to indicate the degradation of the electrode. d) Gravimetric capacity vs. C-Rate plot, comparing the performance of BBL (blue line) to NDI-T2/g3T2 polymers and e) comparison of the utilized theoretical capacity of BBL and NDI-T2/g3T2 polymers.

6.5 Calculation of the theoretical capacity of the polymers

The theoretical capacity was calculated by assuming that two electrons can be added per repeat unit. $C_{theoretical}[\frac{mAh}{g}] = \frac{nF*1000}{M*3600}$ with M being the molecular weight of one repeat unit as shown in Table S1.

Table S1: Summary of the theoretical and measured capacities of the polymers

Polymer	Electrons/ repeat unit	M (repeat unit) [g/mol]	Theoretical capacity [mAh/g]	Measured capacity [mAh/g]	Utilization of theoretical capacity (%)
p([100:0]NDI-g3T2)	2	1398.61	38.3	2.2	5.7
p([90:10]NDI-g3T2)	2	1390.24	38.6	21.7	56.3
p([75:25]NDI-g3T2)	2	1377.68	38.9	27.7	71.2
p([100:0]NDI-T2)	2	1074.24	49.9	8.1	16.2
p([90:10]NDI-T2)	2	1065.87	50.3	34.5	68.6
BBL	1	334.29	80.2	26.6	16.6
BBL	2	334.29	160.3	26.6	33.2

6.5.1 Spectroelectrochemical measurements of p([90:10]NDI-g3T2)

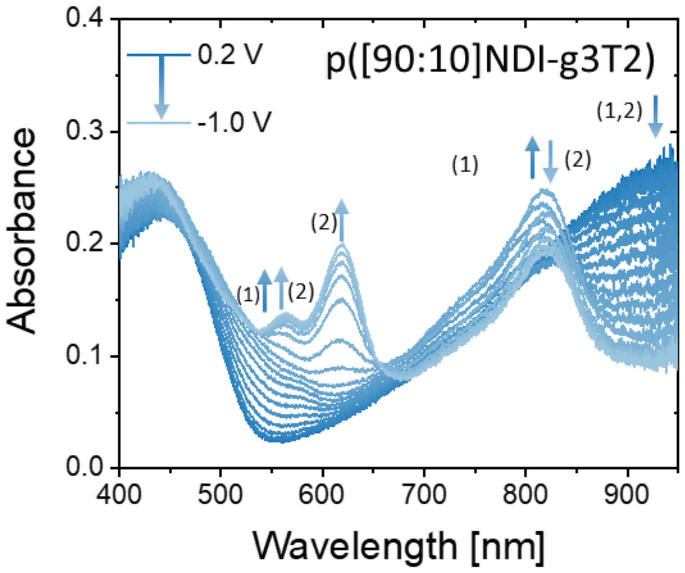

Figure S23. Spectroelectrochemical measurements of p([90:10]NDI-g3T2) monitoring the evolution of the absorption spectrum during the first charging scan of thin film polymers from 0.2 V to – 1.0 V vs. Ag/AgCl with a scan rate of 50 mV/s. (1) and (2) refer to the spectral changes associated with the polaron and bipolaron states, respectively.

6.5.2 Spectroelectrochemical measurements of the p([g7:a]NDI-T2) series

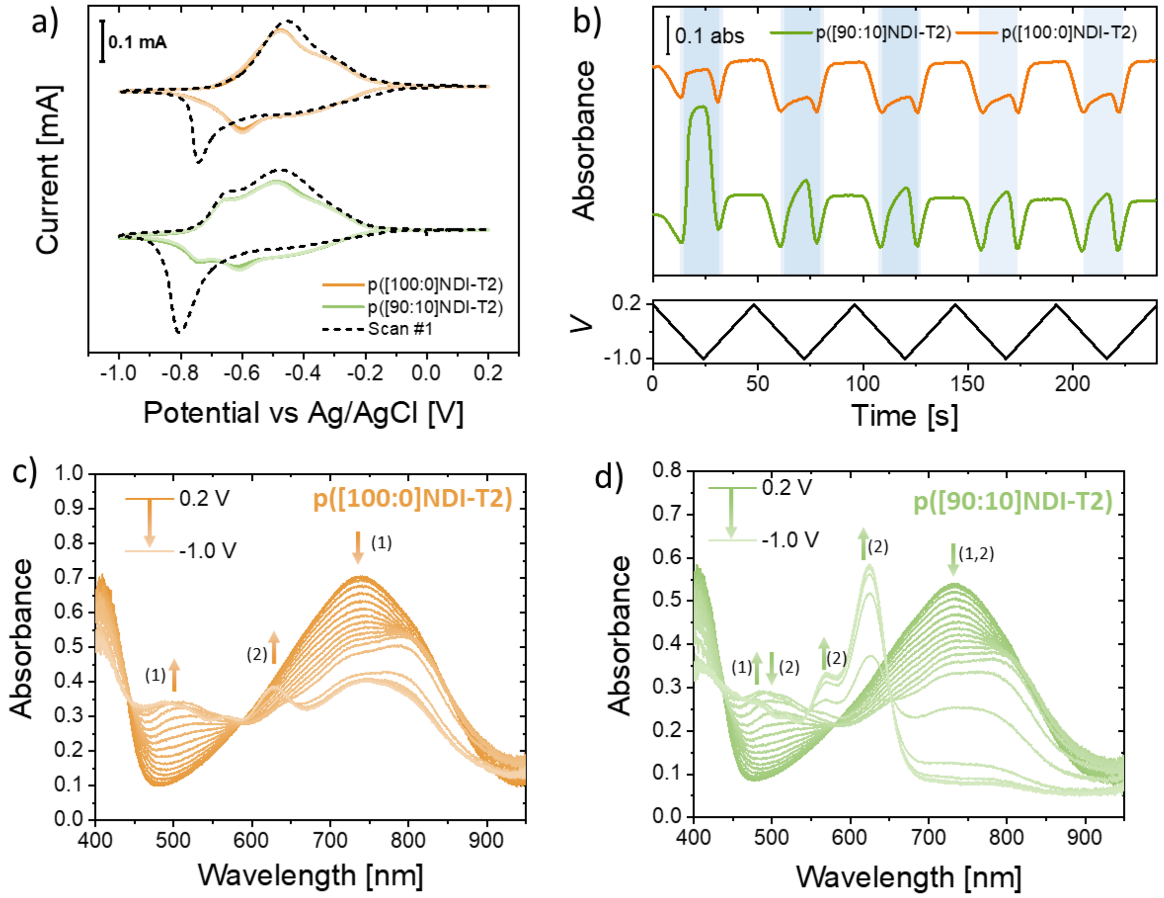

Figure S24. a) CV measurements of the polymers p([100:0]NDI-T2) (orange line) and p([90:10]NDI-T2) (green line) in 0.1 M NaCl aqueous electrolytes with a scan rate of 50 mV/s vs. Ag/AgCl, showing 5 cycles (first cycle is highlighted (dashed line)). The electrolyte was degassed with argon for 15 min prior to the recording of the voltammograms. b) Summary of the spectroelectrochemical measurements, monitoring of the bipolaron absorption peak at $\lambda_{max,\ bipolaron}$ = 624 nm. The time during which the bipolaron is formed is highlighted in blue. Changes of the absorption spectrum of polymer thin films on ITO substrates during the first charging cycle of c) p([100:0]NDI-T2) and d) p([75:25]NDI-T2), between 0.2 V to – 1.0 V vs. Ag/AgCl with a scan rate of 50 mV/s. (1) and (2) refer to the spectral changes associated with the polaron and bipolaron states, respectively.

6.5.3 Difference of the absorption spectrum of p([100:0]NDI-g3T2), p([90:10]NDI-g3T2) and p([75:25]NDI-g3T2) during continuous charging

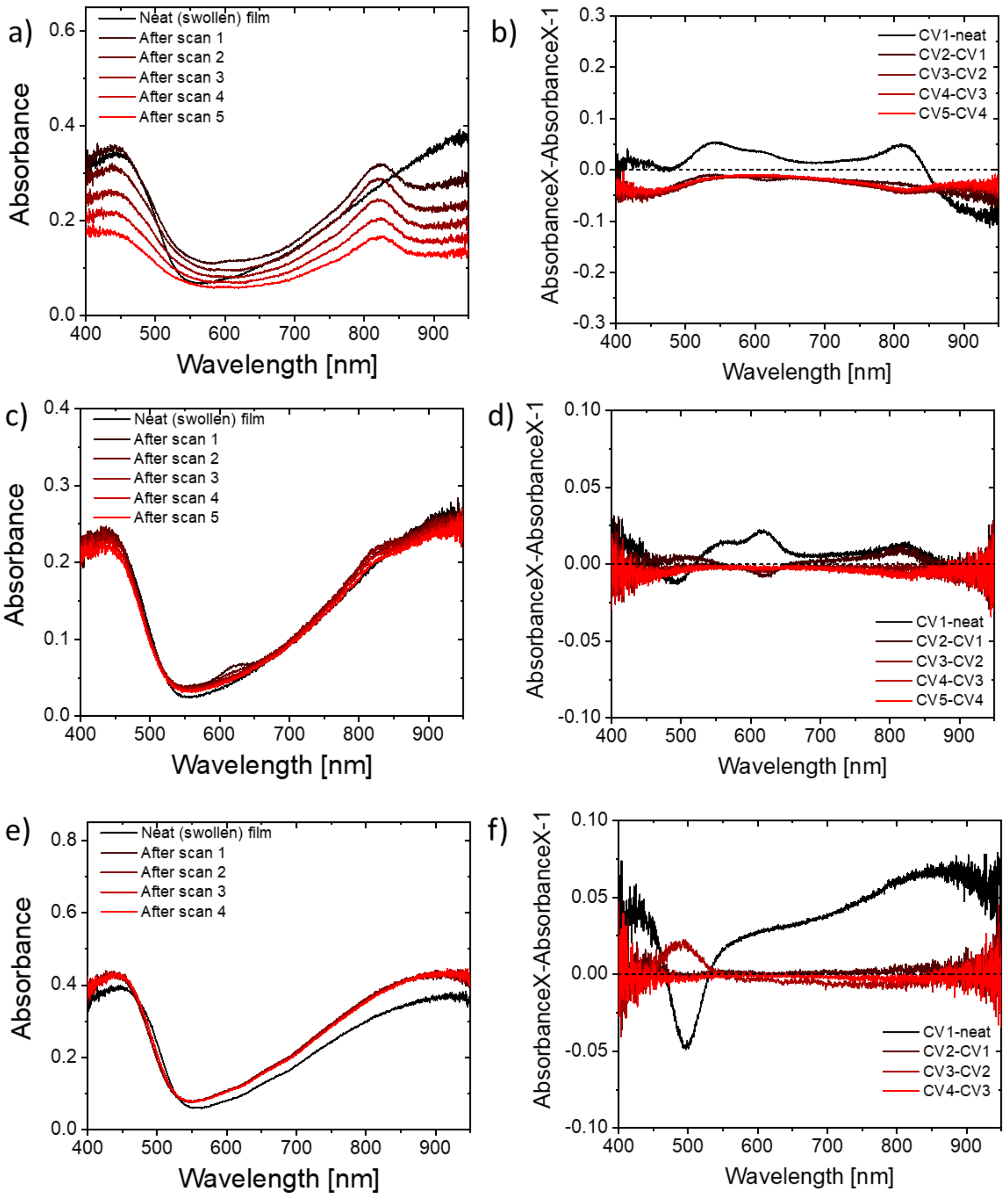

Figure S25: Changes of the absorption spectrum of a) p([100:0]NDI-g3T2), c) p([90:10]NDI-g3T2) and e) p([75:25]NDI-g3T2) and the corresponding changes of the absorption spectra during each charging cycle (when the polymer is in its discharged state at 0 V vs. Ag/AgCl) for f b) p([100:0]NDI-g3T2), d) p([90:10]NDI-g3T2) and f) p([75:25]NDI-g3T2) Stability of the polymer electrodes during continuous charging/discharging in aqueous electrolytes.

6.5.4 Difference of the absorption spectrum of p([100:0]NDI-T2) and p([90:10]NDI-T2) during continuous charging

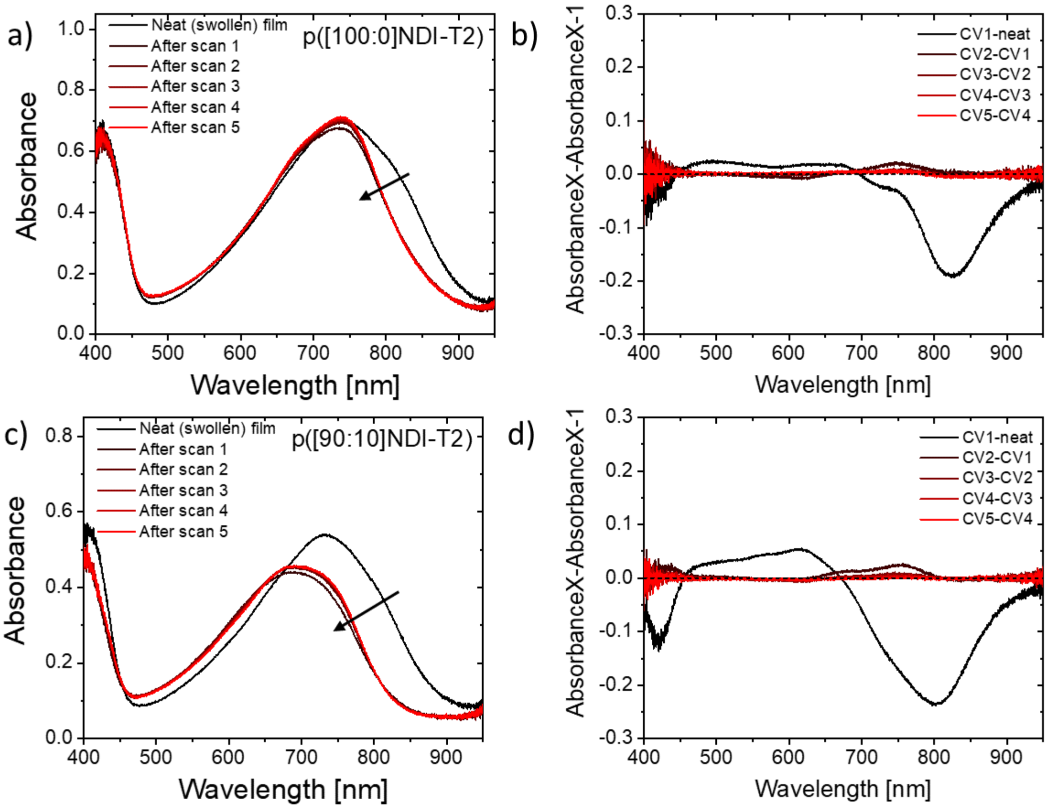

Figure S26: Changes of the absorption spectrum of a) p([100:0]NDI-T2) and c) p([90:10]NDI-T2) and the corresponding changes of the absorption spectra during each charging cycle (when the polymer is in its discharged state at 0 V vs. Ag/AgCl) for b) p([100:0]NDI-T2) and d) p([90:10]NDI-T2).

6.5.5 Comparison of charging of a neat and pre-cycled polymer thin film

To investigate the changes of the cyclic voltammogram and absorption spectrum during the first and second charging/discharging cycle, the electrochemical and spectroelectrochemical properties of a freshly prepared polymer thin film were compared to the same film after pre-cycling. The pre-cycled polymer film was washed with with DI water and dried in ambient conditions (no heating). A similar response between the first and second scan was observed for the pristine polymer and the pre-cycled polymer.

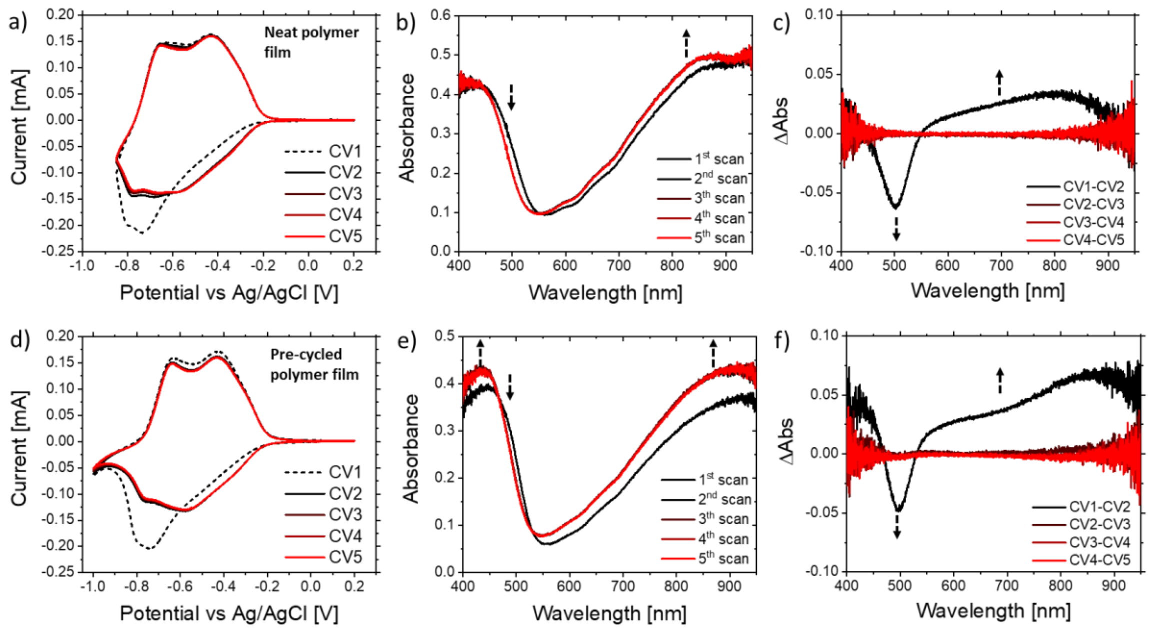

Figure S27. CV and spectroelectrochemical measurements of p([75:25]NDI-g3T2) showing a) the cyclic voltammogram of five scans with a scan rate of 50 mV/s, b) absorption spectrum of the discharged polymer (spectra reported at 0 V vs/ Ag/AgCl), c) changes of the absorption spectrum during each charging/discharging cycle, d) CV of the pre-cycled film, e) absorption spectrum of the discharged polymer (pre-cycled polymer film), and f) changes of the absorption spectrum during each charging/discharging cycle (discharged polymer film).

6.6 Retention experiments of the reduced polymer with low and high O_2 concentrations

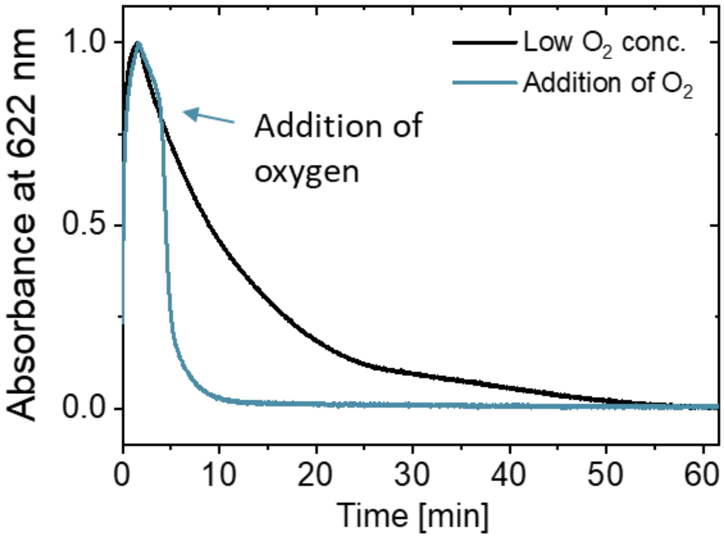

Figure S28.: Spectroelectrochemical measurements of p([75:25]NDI-g3T2) monitoring the changes of the bipolaronic absorbance peak (622 nm) in 0.1 M NaCl at low O_2 conc. and after the addition of O_2.

7 eQCM-D measurements

We attempted conducting measurements at low oxygen concentrations to limit faradaic side reactions between the charged polymer and molecular oxygen, however only with limited success, as only low coulombic efficiencies are observed during the cycling (Figure S24 and Figure S25).

7.1 eQCM-D measurements of the p([g7:a]NDI-g3T2) series charging to –0.4 V vs. Ag/AgCl

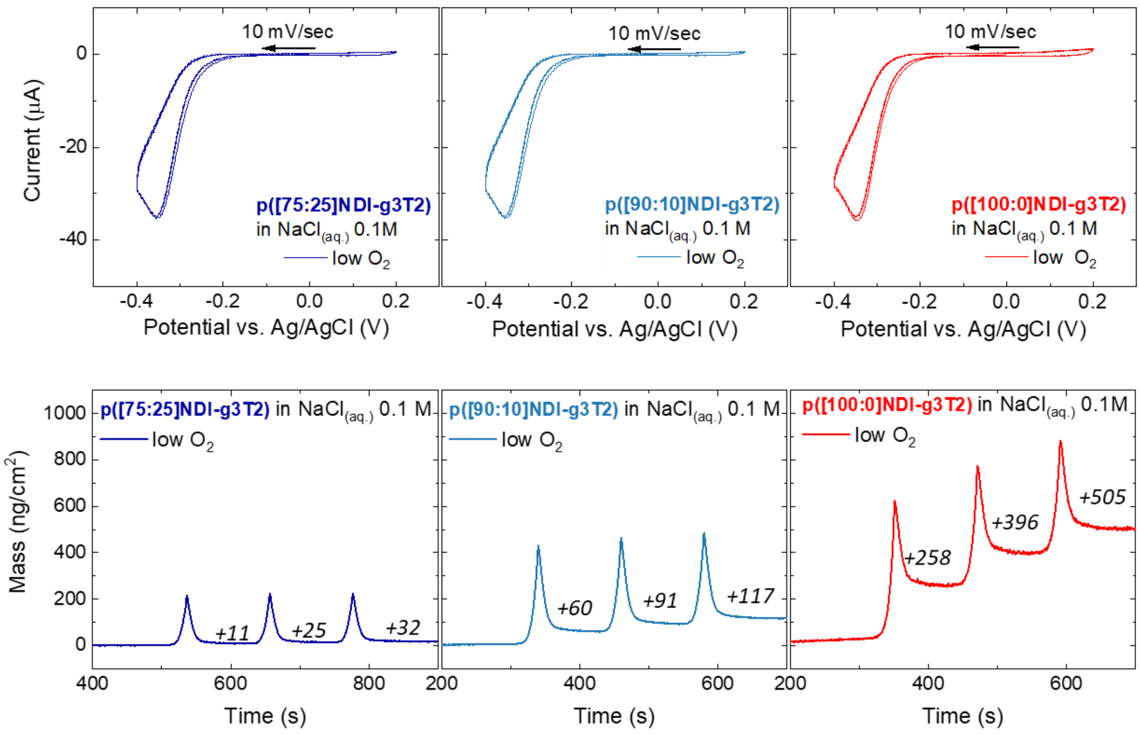

Figure S29. The top panel shows the current recorded during the cyclic voltammetry for p([75:25]NDI-g3T2), p([90:10]NDI-g3T2) and p([100:0]NDI-g3T2) in a 0.1 M NaCl aqueous solution with low O_2 conc. and a scan rate of 10 mV/s. The corresponding, in-situ, mass changes calculated from the eQCM-D data (frequency and dissipation of energy – not shown) using viscoelastic modelling are shown at the bottom.

7.2 eQCM-D measurements of the p([g7:a]NDI-T2) series charging to –0.4 V vs. Ag/AgCl

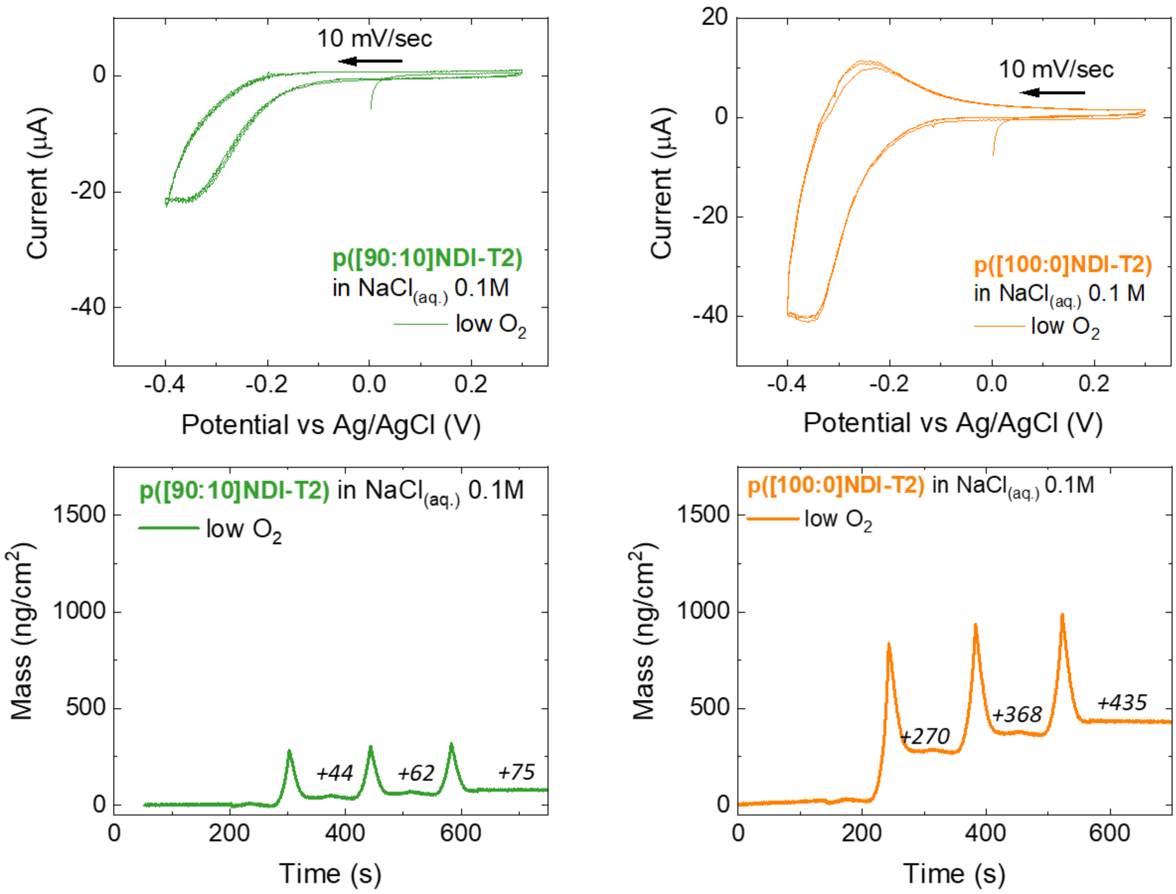

Figure S30. The top panel shows the current recorded during the cyclic voltammetry for p([90:10]NDI-T2) and p([100:0]NDI-T2) in a 0.1 M NaCl aqueous solution with low O_2 conc. and a scan rate of 10 mV/s. The corresponding, in-situ, mass changes calculated from the eQCM-D data (frequency and dissipation of energy – not shown) using viscoelastic modelling are shown at the bottom.

7.3 eQCM-D measurements of the p([g7:a]NDI-g3T2) series to <–0.4 V vs. Ag/AgCl

We also conducted measurements at potential < –0.4 V vs. Ag/AgCl and observed a large dissipation of energy values of more than 1500 $*10^{-6}$ J for the low glycol content polymer, indicative of the formation of a hydrogel-like state of the polymer under high degree of charging. We believe these changes are more drastic for the polymers containing more glycolated side chains and one reason why irreversible changes in the eQCM-D signal are observed at high degree of charging.

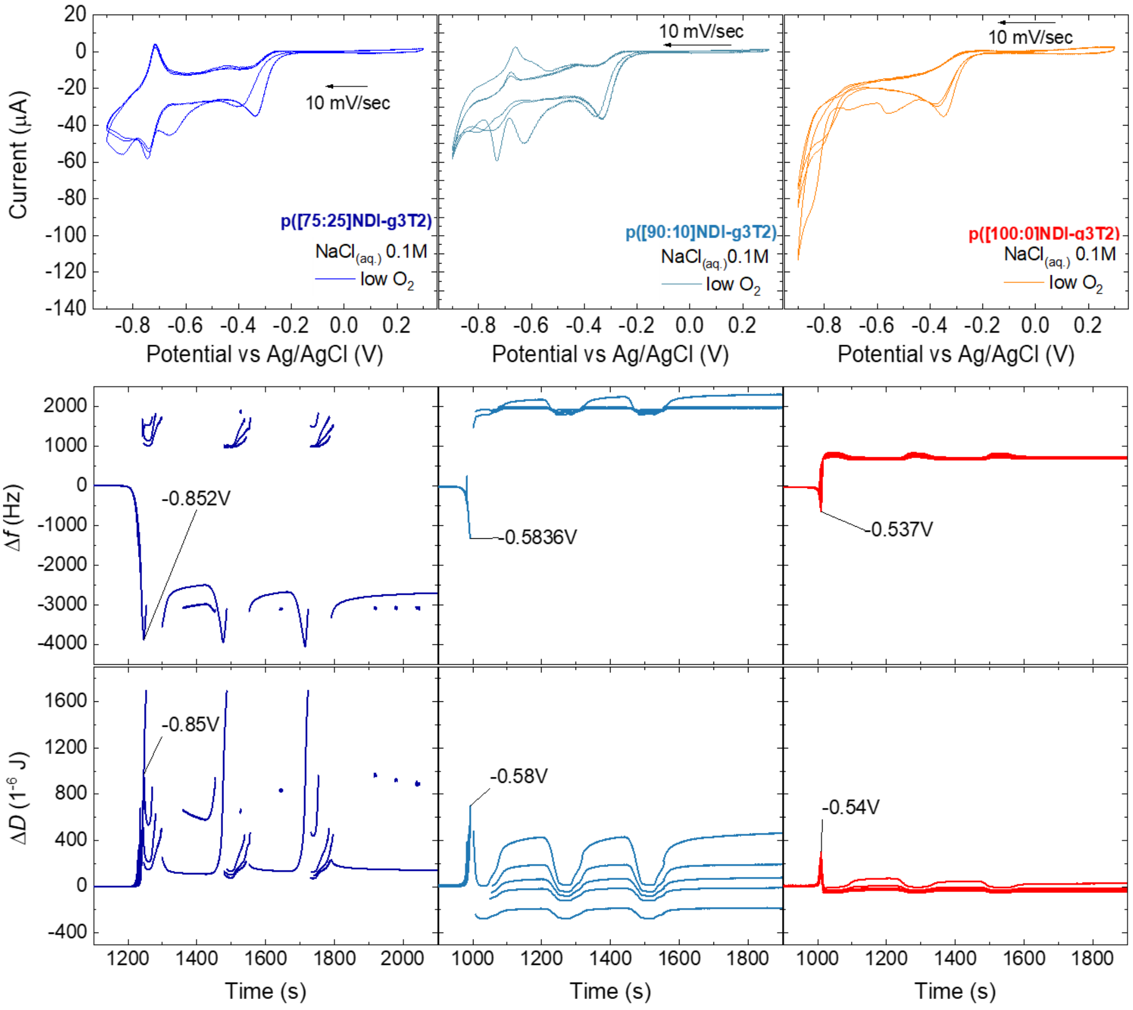

Figure S31. eQCM-D measurements of the p([g7:a]NDI-g3T2) series when applying potentials <–0.4 V vs. Ag/AgCl. The frequency (top row) and dissipation of energy (bottom row) are shown for three cycles of charging/discharging cycles, where potentials between 0.3 V and – 0.9 V are applied for a) p([75:25]NDI-g3T2), b) p([90:10]NDI-g3T2) and c) p([100:0]NDI-g3T2) with a scan rate of 10 mV/s in 0.1 M NaCl with low concentration.

8 Organic electrochemical transistor (OECT) measurements

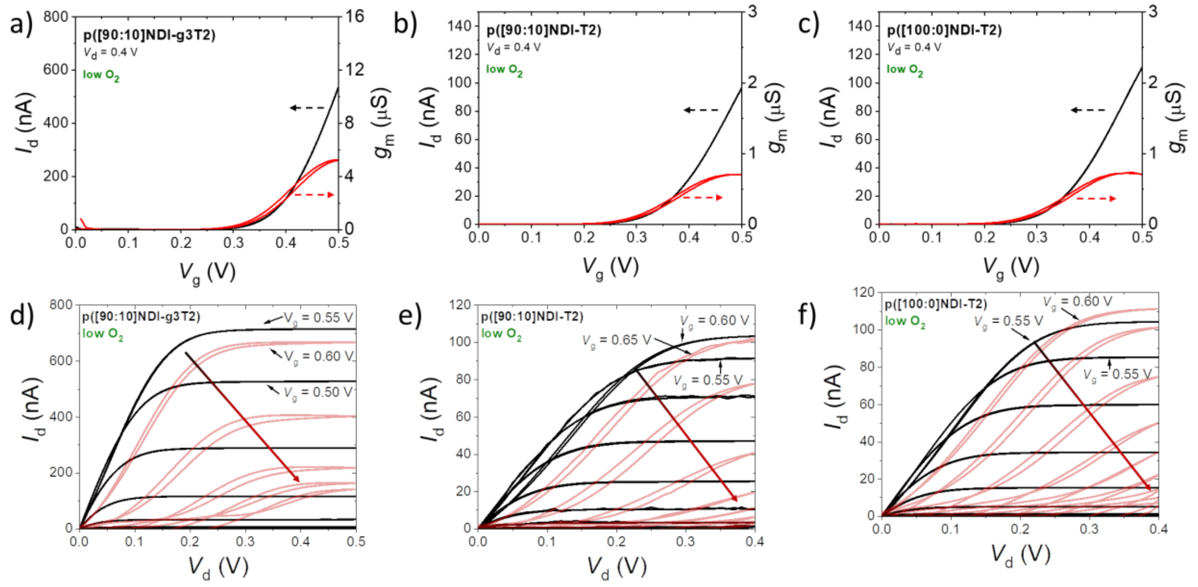

Figure S32. OECT operation in 0.1 M NaCl with a scan rate of 200 mV/s at low O_2 concentration showing the transfer curves for a) p([90:10]NDI-g3T2), b) p([90:10]NDI-T2) and c) p([100:0]NDI-T2). The output curves include data beyond potentials of the stable regime d) p([90:10]NDI-g3T2), e) p([90:10]NDI-T2) and f) p([100:0]NDI-T2).

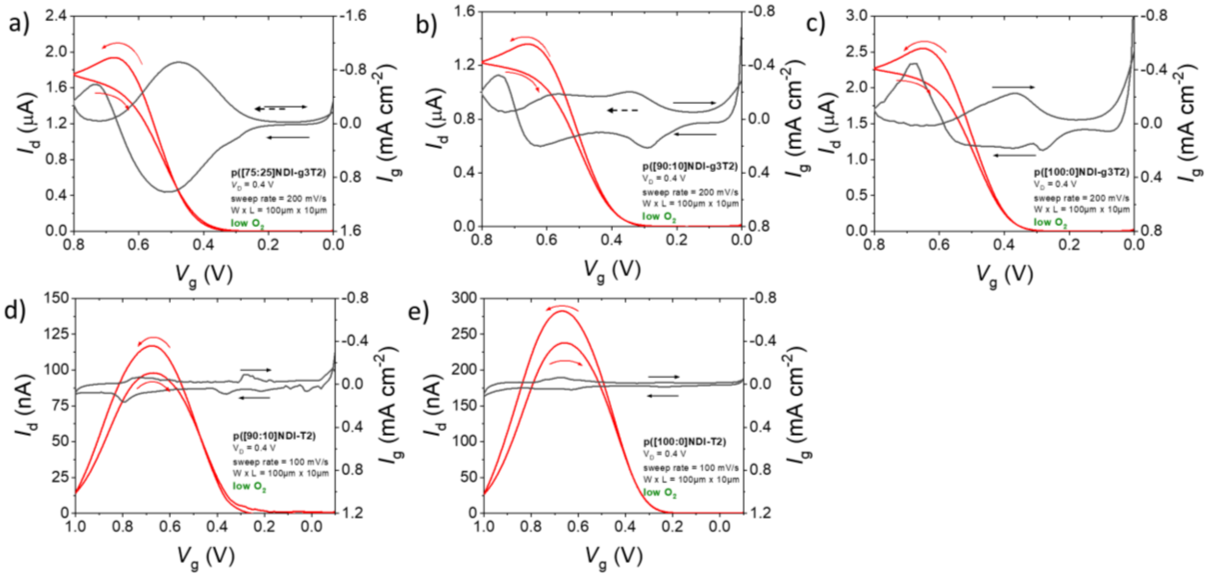

Figure S33. OECT operation in 0.1 M NaCl with a scan rate of 200 mV/s at low O_2 concentration showing the transfer curves for when charging the polymers to the bipolaronic state a) p([75:25]NDI-g3T2), b) p([90:10]NDI-g3T2), c) p([100:0]NDI-g3T2), d) p([90:10]NDI-T2) and e) p([100:0]NDI-T2).

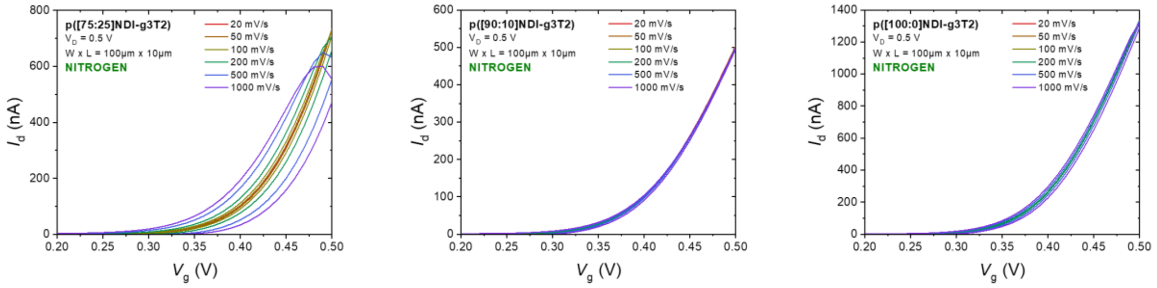

Figure. S34 OECT hysteresis of the p([g7:a]NDI-g3T2) series shown with sweep rate dependent transfer curves in the saturation regime in 0.1 M NaCl with low oxygen concentrations.

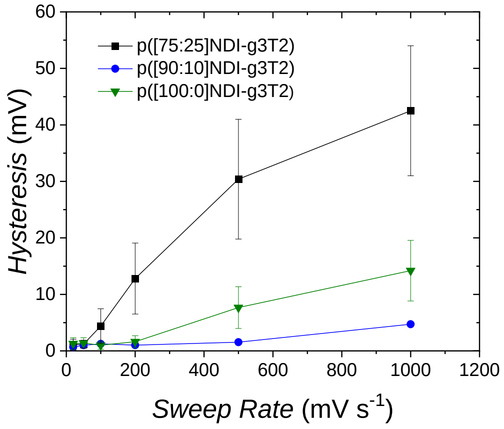

Figure S35. OECT hysteresis: average sweep rate dependent voltage hysteresis of the p([g7:a]NDI-g3T2) series in drop cast OECTs measured at 1/2 $I_{d,max}$. Error bars represent one standard deviation.

Table S2. OECT parameters

	Low O_2	
Polymer	V_t (mV)	μC^* (F cm^{-1} V^{-1} s^{-1})
p([75:25]NDI-g3T2)	357 ± 3	0.023 ± 0.005
p([90:10]NDI-g3T2)	321 ± 5	0.046 ± 0.023
p([100:0]NDI-g3T2)	327 ± 10	0.012 ± 0.009
p([90:10]NDI-T2)	~270	~0.04
p([100:0]NDI-T2)	~290	~0.04

9 DFT and TDDFT simulations

9.1 Method

Molecules in their neutral, singly and doubly reduced states were optimized at the DFT level (B3LYP/6-31g(p,d)). Excited stated TD-DFT calculations were performed on optimized geometries of neutral and charged (reduced) molecules. All calculations shown in this study were performed using Gaussian16. For the polymer series p([g7:a]NDI-g3T2) and p([g7:a]NDI-T2), we approximated the polymer chain to be dimers (spectroscopy calculations) and the calculated structures are shown in Figure S36 and S37.

9.2 Results

The calculated spectra of the charged species supported the interpretation of the changes of the absorption spectrum of the polymers during polaron and bipolaron formation. We also present calculations for the excited states of $(aNDI\text{-}gT2)_2$ and $(aNDI\text{-}T2)_2$ as a comparison to $(gNDI\text{-}gT2)_2$ and $(gNDI\text{-}T2)_2$ to demonstrate that replacing glycol side chain with alkyl side chains on the NDI unit has a little impact on the calculated absorption spectra.

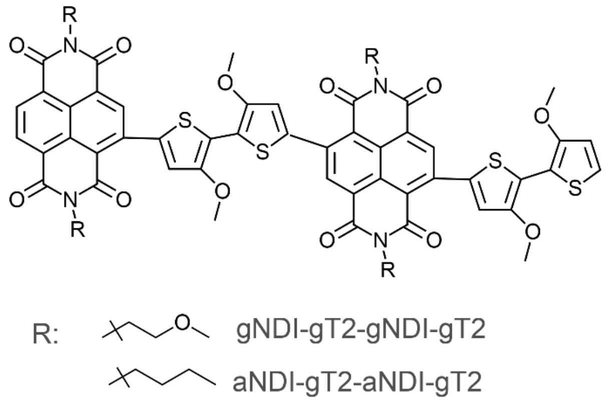

Figure S36. Chemical structure of the $(gNDI\text{-}gT2)_2$ and $(aNDI\text{-}gT2)_2$ dimers.

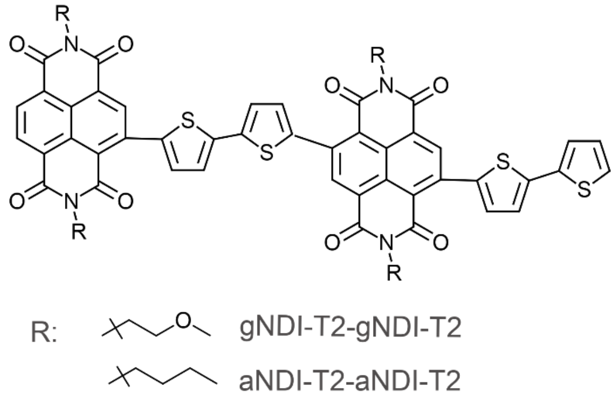

Figure S37. Chemical structure of the $(gNDI\text{-}T2)_2$ and $(aNDI\text{-}T2)_2$ dimers.

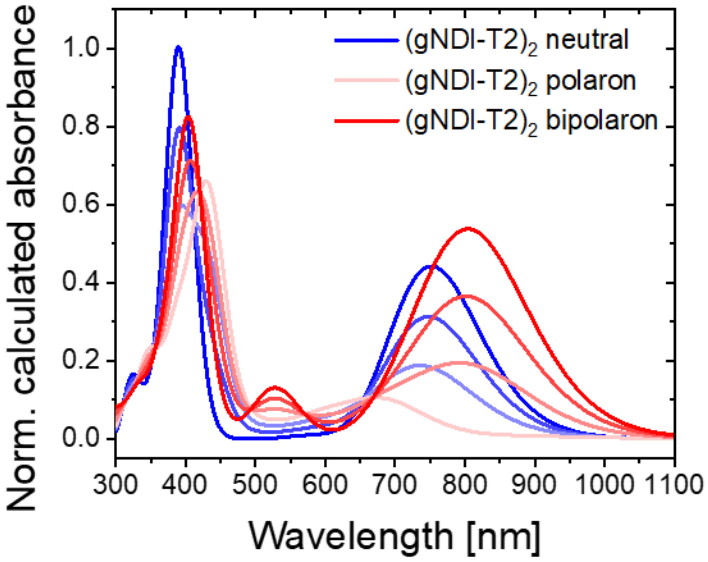

Figure S38. Calculated absorbance spectra (normalized) for a $(gNDI\text{-}T2)_2$ dimer in the neutral (blue line), electron polaron (pink line, one electron per NDI-T2 repeat unit) and electron bipolaron (red line, two electrons per NDI-T2 repeat unit). Linear combinations of the absorption spectra are shown to illustrate the color changes of the absorption spectra.

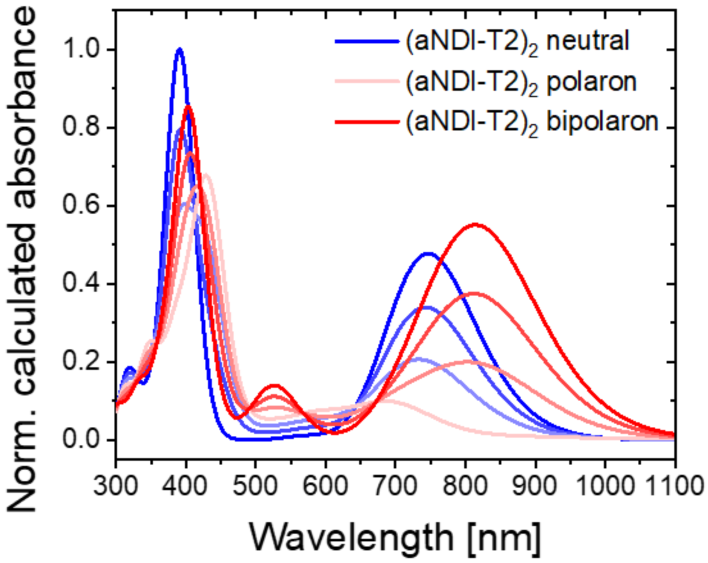

Figure S39. Calculated absorbance spectra (normalized) for a $(aNDI\text{-}T2)_2$ dimer in the neutral (blue line), electron polaron (pink line, one electron per NDI-T2 repeat unit) and electron bipolaron (red line, two electrons per NDI-T2 repeat unit). Linear combinations of the absorption spectra are shown to illustrate the color changes of the absorption spectra.

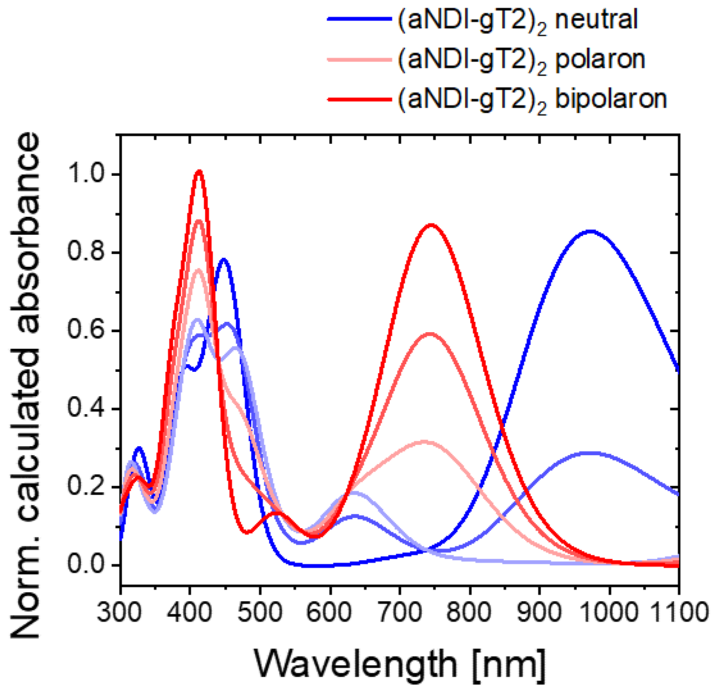

Figure S40. Calculated absorbance spectra (normalized) for a (aNDI-gT2)$_2$ dimer in the neutral (blue line), electron polaron (pink line, one electron per NDI-T2 repeat unit) and electron bipolaron (red line, two electrons per NDI-T2 repeat unit). Linear combinations of the absorption spectra are shown to illustrate the color changes of the absorption spectra.

9.3 Summary of the calculations

Table S3. Energies and oscillator strengths calculated for $(gNDI\text{-}gT2)_2$, $(gNDI\text{-}gT2)_2^{-2}$ and $(gNDI\text{-}gT2)_2^{-4}$ in water (PCM) up to 3eV (400nm). The collective entry in the columns indicates group of states in the given energy interval and sum of oscillator strengths of these states.

**Neutral $(gNDI\text{-}gT2)_2$**		**$(gNDI\text{-}gT2)_2^{-2}$**		**$(gNDI\text{-}gT2)_2^{-4}$**	
Energy [eV (nm)]	Oscillator strength	Energy [eV (nm)]	Oscillator strength	Energy [eV (nm)]	Oscillator strength
1.25 (991)	1.20	0.74 (1672)	1.4	1.68 (737)	1.11
1.46 (847)	0.004	1.34 (925)	0.006	1.79 (694)	0.003
1.53 (810)	0.007	1.56 (792)	0.01	1.89 (655)	0.19
1.69 (733)	0.04	1.71 (724)	0.006	1.96 (634)	0.06
2.65 (468) –	0.59	1.97 (630)	0.25	2.27 (546)	0.008
2.74 (453)		2.16 (573) –	0.14	2.34 (529)	0.14
2.79 (444)	0.32	2.46 (503)		2.42 (512) –	0.06
2.83 (439)	0.24	2.59 (478)	0.26	2.72 (457)	
2.90 (427) –	0.21	2.65 (467)	0.39	2.91 (426)	0.11
3.10 (400)		2.70 (459) –	0.65	2.95 (421)	0.28
		3.03 (409)		3.02 (410)	0.79
				3.04 (408)	0.07
				3.06 (405)	0.10

Table S4. Energies and oscillator strengths calculated for (aNDI-gT2), $(aNDI\text{-}gT2)^{-2}$ and $(aNDI\text{-}gT2)^{-4}$ in water (PCM) up to 3eV (400nm). The collective entry in the columns indicates group of states in the given energy interval and sum of oscillator strengths of these states.

**Neutral $(aNDI\text{-}gT2)_2$**		**$(aNDI\text{-}gT2)_2^{-2}$**		**$(aNDI\text{-}gT2)^{-4}$**	
Energy [eV (nm)]	Oscillator strength	Energy [eV (nm)]	Oscillator strength	Energy [eV (nm)]	Oscillator strength
1.27 (991)	1.18	0.74 (1676)	1.4	1.65 (752)	1.12
1.49 (834)	0.002	1.34 (920)	0.007	1.75 (709)	0.0006
1.55 (799)	0.005	1.57 (788)	0.01	1.86 (665)	0.20
1.72 (723)	0.03	1.73 (716)	0.008	1.92 (647)	0.05
2.68 (463) –	0.66	1.95 (636)	0.25	2.27 (545)	0.003
2.76 (449)		2.17 (571) –	0.14	2.35 (527)	0.15
2.82 (440)	0.33	2.46 (503)		2.43 (511) –	0.06
2.86 (434)	0.18	2.59 (477)	0.25	2.71 (458)	
2.93 (423) –	0.18	2.66 (467)	0.41	2.89 (429)	0.11
3.09 (402)		2.71 (457) –	0.67	2.93 (422)	0.32
		3.04 (408)		3.00 (413)	0.75
				3.02 (410)	0.04
				3.05 (406)	0.13

Table S5. Energies and oscillator strengths calculated for $(gNDI\text{-}T2\text{-}g)_2$, $(gNDI\text{-}T2\text{-}g)_2^{-2}$ and $(gNDI\text{-}T2\text{-}g)_2^{-4}$ in water (PCM) up to 3.1eV (400nm). The collective entry in the columns indicates group of states in the given energy interval and sum of oscillator strengths of these states.

**Neutral $(gNDI\text{-}T2\text{-}g)_2$**		**$(gNDI\text{-}T2\text{-}g)_2^{-2}$**		**$(gNDI\text{-}T2\text{-}g)_2^{-4}$**	
Energy [eV (nm)]	Oscillator strength	Energy [eV (nm)]	Oscillator strength	Energy [eV (nm)]	Oscillator strength
1.65 (752)	0.87	0.65 (1900)	1.11	1.52 (814)	0.96
1.81 (685)	0.001	1.40 (889)	0.01	1.61 (767)	0.001
1.92 (646)	0.001	1.67 (743)	0.0003	1.72 (720)	0.23
2.12 (584)	0.02	1.80 (688)	0.18	1.76 (705)	0.0004
2.98 (416)	0.001	2.02 (612) –	0.20	2.27 (546)	0.002
3.05 (407)	0.10	2.39 (518)		2.34 (531)	0.22
3.07 (404)	0.04	2.61 (475) –	0.07	2.40 (518)	0.03
3.12 (397)	0.83	2.70 (458)		2.52 (493)	0.01
		2.81 (441)	0.14	2.59 (479)	0.01
		2.84 (436)	0.44	2.64 (469)	0.006
		2.87 (431)	0.48	2.88 (430)	0.11
		2.99 (415)	0.22	2.97 (416)	0.07
		3.05 (407)	0.10	3.01 (412)	0.12
		3.07 (404)	0.01	3.02 (410)	0.42
				3.06 (405)	0.72

Table S6. Energies and oscillator strengths calculated for $(aNDI\text{-}T2)_2$, $(aNDI\text{-}T2)_2^{-2}$ and $(aNDI\text{-}T2)_2^{-4}$ in water (PCM) up to 3.1eV (400nm). The collective entry in the columns indicates group of states in the given energy interval and sum of oscillator strengths of these states.

**Neutral $(aNDI\text{-}T2)_2$**		**$(aNDI\text{-}T2)_2^{-2}$**		**$(aNDI\text{-}T2)_2^{-4}$**	
Energy [eV (nm)]	Oscillator strength	Energy [eV (nm)]	Oscillator strength	Energy [eV (nm)]	Oscillator strength
1.66 (747)	0.91	0.66 (1869)	1.12	1.50 (825)	0.94
1.83 (676)	0.002	1.39 (892)	0.02	1.59 (778)	0.002
1.93 (644)	0.007	1.69 (736)	0.001	1.69 (732)	0.23
2.15 (575)	0.02	1.78 (696)	0.17	1.74 (712)	0.01
2.98 (415)	0.006	2.05 (604) –	0.20	2.27 (545)	0.002
3.05 (406)	0.18	2.38 (519)		2.34 (529)	0.23
3.08 (403)	0.09	2.61 (475) –	0.06	2.40 516)	0.03
3.12 (398)	0.80	2.70 (459)		2.51 (494)	0.01
		2.81 (441)	0.11	2.58 (481)	0.01
		2.84 (436)	0.30	2.64 (469)	0.001
		2.87 (432)	0.63	2.87 (431)	0.10
		2.98 (414)	0.22	2.96 (419)	0.07
		3.05 (407)	0.06	2.99 (415)	0.03
		3.06 (405)	0.06	3.02 (410)	0.32
				3.05 (406)	0.90

9.4 Coordinates of optimized structures

9.4.1 (gNDI-gT2)$_2$ Neutral optimized structure in water (PCM)

C	-5.05513600	-3.19008000	-0.37862900
C	-5.37957500	-1.75748400	-0.19736300
C	-4.35378000	-0.78802600	-0.05174100
C	-2.98201000	-1.16345300	-0.07597300
C	-2.65778600	-2.61841000	-0.05258800
C	-6.70446600	-1.39355500	-0.13809200
C	-4.76016700	0.56946800	0.10354000
C	-6.13482400	0.94502800	0.12888000
C	-7.13740000	-0.05464300	0.06424400
C	-6.45473100	2.39845200	0.09981100
C	-4.05428100	2.97023100	0.41507800
C	-3.73318500	1.53684600	0.24254300
C	-2.40574800	1.17139900	0.18132800
C	-1.98050800	-0.16684100	-0.01130700
H	-1.66778200	1.96222100	0.24464700
H	-7.43953500	-2.18649600	-0.20785900
N	-5.40299800	3.30580700	0.34106500
N	-3.70512600	-3.52505100	-0.30637800
O	-5.91237500	-4.04872200	-0.56201400
O	-1.54316500	-3.06247300	0.18990000
O	-3.19460100	3.82856000	0.59218900
O	-7.56867600	2.84897600	-0.13811400
C	-5.75170000	4.73232600	0.43569500
H	-6.75270800	4.80452300	0.85817500
H	-5.03330600	5.20069700	1.10739400
C	-3.35396800	-4.95051600	-0.40952500
H	-4.06780800	-5.41477100	-1.08889300
H	-2.35062000	-5.01789100	-0.82734300
C	-8.57237300	0.13940600	0.25684700
C	-9.23758600	0.96246900	1.14325300
S	-9.71519300	-0.88386300	-0.60623300
C	-10.63727700	0.78277400	1.13327300
H	-8.72917900	1.66657800	1.78439000
C	-11.08889100	-0.18317200	0.22707400
O	-11.54266400	1.44066600	1.90365400
C	-12.44428600	-0.57169700	-0.04574400
C	-11.05430800	2.46288200	2.77653500
C	-12.88942500	-1.54407500	-0.94059600
S	-13.83419400	0.16521500	0.75339900
H	-11.93205500	2.88092700	3.26849300
H	-10.37686600	2.04677700	3.52997900
H	-10.53959600	3.24859300	2.21310600
C	-14.30676500	-1.69315800	-0.98282400
O	-11.98286600	-2.24014800	-1.67202700
C	-14.93862300	-0.83724500	-0.12346000
H	-14.82864500	-2.39802300	-1.61607200
C	-12.47349500	-3.23550600	-2.57563200
H	-16.00145700	-0.73941400	0.04856100
H	-13.11939200	-2.78906400	-3.33884800
H	-11.59317200	-3.66756800	-3.05042900
H	-13.02155400	-4.01723700	-2.03946800
C	-5.71327500	5.42421000	-0.92615900
H	-4.70752600	5.33853100	-1.36546400
H	-6.42688000	4.94523900	-1.61307200
C	-3.39865500	-5.65286500	0.94683200
H	-4.40679600	-5.57194800	1.38144300
H	-2.68935500	-5.17833300	1.64127300
O	-6.05407000	6.78126400	-0.70619100
O	-3.05477800	-7.00752400	0.71776600
C	-6.05753400	7.54196600	-1.90283700
H	-6.78933400	7.15454900	-2.62681100
H	-6.32955300	8.56563600	-1.63613800
H	-5.06726200	7.54835300	-2.38151200

C	-3.05650700	-7.77795500	1.90825100
H	-2.78166300	-8.79901100	1.63457300
H	-2.32901400	-7.39544700	2.63911600
H	-4.04926500	-7.78956700	2.38161000
C	-0.54138500	-0.37449600	-0.20032000
S	0.60230300	0.55439200	0.75241100
C	0.11088500	-1.14381700	-1.14002900
C	1.97445000	-0.11072400	-0.11828700
C	1.51575000	-0.99812400	-1.09877000
H	-0.40757800	-1.78467800	-1.83795900
C	3.33139000	0.23008700	0.18728300
O	2.41684200	-1.61263600	-1.90321400
S	4.70107600	-0.42363500	-0.69262300
C	3.78991800	1.11719600	1.16942100
C	1.92539700	-2.55664000	-2.86120000
C	5.84610700	0.51458000	0.25303400
C	5.19206000	1.27542400	1.19999300
O	2.88875600	1.72098200	1.98250300
H	1.26669100	-2.07007600	-3.58791200
H	2.80449900	-2.94887600	-3.37112100
H	1.39097100	-3.37410100	-2.36639800
C	7.28355800	0.32653800	0.05137300
H	5.70921800	1.91916700	1.89588800
C	3.37846000	2.67391500	2.93224200
C	7.71299000	-1.01063500	-0.19059200
C	8.27201300	1.33485800	0.14397200
H	2.49979900	3.05952000	3.44797400
H	4.04809600	2.19723100	3.65560100
H	3.90074700	3.49455500	2.42963200
C	9.03974200	-1.36103700	-0.27138900
H	6.97473100	-1.79928600	-0.27499900
C	9.65184700	0.97311900	0.10912400
C	7.94000600	2.78698000	0.15634400
C	9.40735200	-2.78157200	-0.50261100
C	10.04286200	-0.37938400	-0.09544800
C	10.67954700	1.93784900	0.27692800
N	8.98934300	3.69150600	0.41404300
O	6.82150700	3.23439800	-0.06380900
N	10.77523700	-3.07783400	-0.55776400
O	8.56881200	-3.66337100	-0.64474200
C	11.40948600	-0.73522100	-0.12861900
C	10.34326100	3.36299300	0.48002300
C	12.01708100	1.56692200	0.25106500
C	8.63378100	5.11404100	0.54164600
C	11.80626600	-2.14788200	-0.35432900
C	11.15993100	-4.47394300	-0.81904400
C	12.38866500	0.23091800	0.04813900
O	11.19445000	4.22521300	0.67578700
H	12.76885200	2.33510600	0.38933500
H	7.63330500	5.17175700	0.96758100
H	9.35110100	5.57079800	1.22232000
C	8.66619400	5.83631400	-0.80450900
O	12.97795300	-2.50486300	-0.37172600
H	10.39140500	-4.91648500	-1.45133800
H	12.11079600	-4.46182300	-1.35006700
C	11.29668400	-5.27796000	0.47321800
H	13.43209100	-0.05989500	0.02333300
H	9.67134600	5.76472200	-1.24767600
H	7.95321900	5.36975600	-1.50061600
O	8.32006600	7.18660000	-0.55371000
H	10.34215900	-5.27804200	1.02156500
H	12.06056100	-4.82319100	1.12233000
O	11.66674400	-6.59251900	0.10025200
C	8.31082600	7.97383400	-1.73307600
C	11.83286000	-7.44891100	1.21846900
H	7.57907700	7.59961400	-2.46400300
H	8.03508100	8.99010400	-1.44295300
H	9.30002600	7.99514000	-2.21352400
H	12.11582800	-8.43169600	0.83512500

H	12.62326900	-7.08631600	1.89182700
H	10.90216500	-7.54479600	1.79659100

9.4.2 (aNDI-gT2)$_2$ Neutral optimized structure in water (PCM)

C	-5.05038700	-3.19224400	-0.40270600
C	-5.37793900	-1.76108800	-0.20915100
C	-4.35387700	-0.79168400	-0.05564500
C	-2.98225100	-1.16671500	-0.08253700
C	-2.65758900	-2.62303300	-0.07143900
C	-6.70300400	-1.39794000	-0.14675900
C	-4.76078400	0.56420100	0.10974100
C	-6.13512400	0.93951700	0.13659700
C	-7.13612300	-0.06037900	0.06466900
C	-6.45478600	2.39478100	0.11719900
C	-4.05986400	2.96335100	0.44482700
C	-3.73552600	1.53153600	0.25853300
C	-2.40802700	1.16690200	0.19541600
C	-1.98243900	-0.16981200	-0.00906200
H	-1.66997800	1.95696700	0.26705600
H	-7.43805500	-2.19037900	-0.22247300
N	-5.40655900	3.30011900	0.36904900
N	-3.70217900	-3.52797800	-0.33182200
O	-5.90969500	-4.04796800	-0.59487400
O	-1.54036200	-3.06420300	0.16738800
O	-3.19851800	3.81842800	0.63411200
O	-7.57015000	2.84246100	-0.12212700
C	-8.57199300	0.13225200	0.25839200
C	-9.23660600	0.94454300	1.15440900
S	-9.71483800	-0.87920700	-0.61765600
C	-10.63697600	0.76619100	1.14143200
H	-8.72762000	1.64066600	1.80385700
C	-11.08886600	-0.18799000	0.22381100
O	-11.54200600	1.41599400	1.91966100
C	-12.44468100	-0.57330000	-0.05398700
C	-11.05249800	2.42631900	2.80540000
C	-12.89028200	-1.53426400	-0.96040300
S	-13.83424700	0.15383300	0.75462500
H	-11.92971300	2.83911900	3.30276900
H	-10.37544200	2.00005500	3.55352500
H	-10.53691200	3.21872500	2.25218900
C	-14.30780300	-1.68265300	-1.00403100
O	-11.98408500	-2.22183300	-1.70102700
C	-14.93949300	-0.83747400	-0.13409200
H	-14.82984500	-2.37965400	-1.64582400
C	-12.47581700	-3.20577000	-2.61617200
H	-16.00223600	-0.74155800	0.03949600
H	-13.12226800	-2.75001600	-3.37345100
H	-11.59612300	-3.63241500	-3.09706300
H	-13.02374700	-3.99394900	-2.08930600
C	-5.75357600	4.73387000	0.45305100
C	-5.69646200	5.44103700	-0.90484300
H	-5.04559400	5.18497100	1.14903200
H	-6.75698300	4.79381400	0.87439500
C	-6.05702900	6.92754700	-0.79469300
H	-4.68713500	5.33533200	-1.32057300
H	-6.38532400	4.94168400	-1.59608900
C	-6.00257500	7.65324100	-2.14234900
H	-7.06304800	7.02640800	-0.36559200
H	-5.37326800	7.41590800	-0.08757200
H	-6.26435900	8.71075800	-2.03522500
H	-4.99916000	7.60019500	-2.57951500
H	-6.70060900	7.20737200	-2.85977700
C	-3.35249100	-4.96068600	-0.42482900
C	-3.40945900	-5.67702400	0.92824400
H	-2.34858500	-5.01565900	-0.84578600
H	-4.05873100	-5.40833600	-1.12477900
C	-3.04629000	-7.16214700	0.80804000
H	-2.72190700	-5.18136000	1.62345700

H	-4.41923100	-5.57584900	1.34393700
C	-3.10040100	-7.89711400	2.15066900
H	-3.72880800	-7.64675200	0.09715900
H	-2.03983900	-7.25635100	0.37893600
H	-2.83679500	-8.95342300	2.03639900
H	-2.40353700	-7.45504700	2.87156800
H	-4.10417900	-7.84873800	2.58753100
C	-0.54276000	-0.37510300	-0.20013100
S	0.60104900	0.53489600	0.77011300
C	0.10921900	-1.12685400	-1.15369300
C	1.97349100	-0.11431400	-0.11264400
C	1.51451100	-0.98248200	-1.10946600
H	-0.40979100	-1.75463300	-1.86312600
C	3.33074800	0.22131200	0.19945200
O	2.41533600	-1.58218800	-1.92610400
S	4.70036300	-0.40993700	-0.69743200
C	3.78975500	1.08581100	1.20060900
C	1.92298000	-2.50839900	-2.90050300
C	5.84564200	0.50804300	0.26738300
C	5.19218200	1.24513800	1.23279400
O	2.88925200	1.67021200	2.02925700
H	1.26317100	-2.00901100	-3.61751000
H	2.80146200	-2.89109200	-3.41875200
H	1.38926200	-3.33508100	-2.42038100
C	7.28362100	0.32736500	0.05765700
H	5.70968500	1.87397000	1.94197100
C	3.37979500	2.60347200	2.99758900
C	7.71489000	-1.00416000	-0.21114900
C	8.26937400	1.33637000	0.16517200
H	2.50194000	2.97644400	3.52395100
H	4.05237900	2.11301600	3.70896300
H	3.89930400	3.43573400	2.51139400
C	9.04198700	-1.34992200	-0.30662300
H	6.97793400	-1.79282700	-0.30617900
C	9.64938100	0.97825700	0.11566600
C	7.93506000	2.78878400	0.20979700
C	9.41368100	-2.76511700	-0.56741000
C	10.04262000	-0.36865500	-0.11810800
C	10.67469000	1.94256400	0.29829500
N	8.98090600	3.68950000	0.48362600
O	6.81272900	3.23449100	0.00279300
N	10.77988500	-3.05755500	-0.63541300
O	8.57355700	-3.64455000	-0.72166000
C	11.40966300	-0.72077100	-0.16684600
C	10.33391000	3.36203400	0.53658900
C	12.01274200	1.57552500	0.25658900
C	8.62396800	5.11638400	0.62667800
C	11.80763500	-2.12895300	-0.42224400
C	11.16739700	-4.45793700	-0.90331600
C	12.38672200	0.24477600	0.02313300
O	11.18652800	4.21969300	0.75018700
H	12.76337500	2.34257900	0.40705200
C	8.65824600	5.87596400	-0.70337300
H	9.33698700	5.54622800	1.33078800
H	7.62551200	5.15204400	1.06234100
O	12.98197700	-2.47898000	-0.45382100
C	11.30535800	-5.28780800	0.37702000
H	12.11352800	-4.42507700	-1.44395200
H	10.39954300	-4.87854800	-1.55306200
H	13.43080300	-0.04249800	-0.01405900
C	8.28759800	7.35400700	-0.53037100
H	9.66287600	5.79473600	-1.13566100
H	7.96432200	5.39849000	-1.40497000
C	11.70601900	-6.73831200	0.08154900
H	12.05544600	-4.82097000	1.02672400
H	10.35240300	-5.26958000	0.91938700
C	8.31808800	8.13246200	-1.84902000
H	7.28671100	7.42752100	-0.08450000
H	8.97693400	7.82003500	0.18634600

C	11.84822200	-7.58472600	1.35002300
H	10.95819900	-7.19361700	-0.58125100
H	12.65342900	-6.74737900	-0.47357800
H	8.04938700	9.18269800	-1.69686000
H	9.31588800	8.10515700	-2.30115000
H	7.61396600	7.70924400	-2.57417300
H	12.13429000	-8.61389100	1.11074500
H	12.61334100	-7.17183700	2.01698600
H	10.90616200	-7.62111500	1.90851500

9.4.3 $(gNDI\text{-}T2)_2$ Neutral optimized structure in water (PCM)

C	5.42620300	-3.10386900	1.02008500
C	5.81248000	-1.75588500	0.54216300
C	4.83185300	-0.77215100	0.26143200
C	3.44533400	-1.05234100	0.42211800
C	3.04443700	-2.46108500	0.71485500
C	7.14917100	-1.48992000	0.34859700
C	5.29045400	0.50366800	-0.17594700
C	6.67762400	0.78192500	-0.34219800
C	7.07858000	2.19055300	-0.63258200
C	4.69556800	2.83846100	-0.92300800
C	4.30971200	1.48792400	-0.45283900
C	2.97172600	1.21953900	-0.26493700
C	2.49730100	-0.03339200	0.20039700
H	2.26607200	2.01975600	-0.45436800
H	7.85379500	-2.29082900	0.53865000
N	6.06249400	3.09524100	-0.99580200
N	4.05900300	-3.36140500	1.09084600
O	6.24971800	-3.96061600	1.32235600
O	1.89528500	-2.87041600	0.63010500
O	3.87156000	3.69766800	-1.21753700
O	8.22876700	2.59883500	-0.55493300
C	9.06187500	-0.15277200	-0.41640600
S	10.23104400	-0.86745700	0.68605500
C	9.70053500	0.31531200	-1.54364000
C	11.56278500	-0.52597300	-0.39945200
C	11.09822400	0.10445000	-1.53929500
H	9.17193500	0.78201900	-2.36482600
C	12.92024900	-0.87870700	-0.04164400
H	11.74619000	0.38908800	-2.36041600
C	13.36247200	-1.75040300	0.93275000
S	14.29040900	-0.16561000	-0.88262500
C	14.78014200	-1.85289800	1.00273700
H	12.68932300	-2.31435200	1.56875300
C	15.41735200	-1.06375300	0.08381400
H	15.30317900	-2.49373600	1.70272100
H	16.47861300	-0.95200200	-0.08915500
C	7.62528800	-0.24007500	-0.12735100
C	6.47711800	4.44923100	-1.39777800
C	6.56747100	5.40021700	-0.20498900
H	5.73979300	4.81898400	-2.10926400
H	7.44894300	4.37022700	-1.88267300
H	5.59220200	5.46558800	0.30140500
H	7.30364000	5.02621900	0.52216200
C	7.07520400	7.64601400	0.29812000
H	7.37485000	8.57777600	-0.18662500
H	6.12079100	7.80553000	0.82096300
H	7.83593000	7.36937900	1.04272200
C	3.64385900	-4.71309600	1.50007800
C	3.55842600	-5.67222500	0.31344200
H	2.67024700	-4.63146500	1.98103400
H	4.37880900	-5.07748900	2.21676000
H	2.82425200	-5.30393000	-0.41858100
H	4.53544100	-5.74001900	-0.18924400
C	3.05415300	-7.92189800	-0.17576300
H	2.75399800	-8.85057500	0.31454700
H	2.29530300	-7.65114300	-0.92442300

H	4.01022200	-8.08407800	-0.69474000
O	3.16946700	-6.93116000	0.83237600
O	6.95604600	6.66234500	-0.71645700
C	1.05550400	-0.13261800	0.46768500
S	-0.09874900	0.52199900	-0.68273900
C	0.40257200	-0.58194700	1.59413600
C	-1.44990200	0.17435500	0.37778400
C	-0.99923300	-0.40922200	1.54842200
H	0.92265400	-1.01293200	2.43998700
C	-2.80435900	0.47464000	-0.02723900
H	-1.66022300	-0.68886700	2.36090300
S	-4.17322900	-0.20032400	0.83429500
C	-3.24276000	1.27435700	-1.06764000
C	-5.31749900	0.63081000	-0.20505300
C	-4.65012000	1.36149700	-1.16273900
H	-2.56750000	1.79324300	-1.73845600
C	-6.76125100	0.38246600	-0.06840500
H	-5.16251800	1.94311900	-1.91856400
C	-7.15964100	-0.97949500	0.02805100
C	-7.76190000	1.37358200	-0.09802800
C	-8.48231900	-1.36071600	0.03882400
H	-6.40249400	-1.75417900	0.05870400
C	-9.13373300	0.98784200	-0.12866600
C	-7.44850000	2.82986800	0.00764000
C	-8.82753400	-2.80377800	0.11870100
C	-9.49928400	-0.38494700	-0.06301000
C	-10.17351400	1.94825700	-0.22963900
N	-8.50782200	3.73564000	-0.18903000
O	-6.33762400	3.26758700	0.27237800
N	-10.19003000	-3.12840300	0.10867600
O	-7.97441900	-3.67938800	0.19229800
C	-10.86078600	-0.76560600	-0.09692100
C	-9.85638000	3.39238600	-0.29712600
C	-11.50250900	1.55436900	-0.26892300
C	-8.17360900	5.16937200	-0.19384800
C	-11.23491700	-2.20107900	-0.02256800
C	-10.55179300	-4.55132600	0.21078300
C	-11.85178600	0.19657500	-0.20232400
O	-10.71931000	4.25440600	-0.42325600
H	-12.26720600	2.31767500	-0.35220900
C	-8.22487800	5.77501300	1.20810500
H	-8.89386900	5.67099800	-0.83884400
H	-7.17187900	5.27716500	-0.60720700
O	-12.39939300	-2.57742600	-0.06807400
C	-10.63878700	-5.21467900	-1.16317900
H	-11.51589800	-4.61300100	0.71362700
H	-9.79054400	-5.04459400	0.81362700
H	-12.89007400	-0.11206600	-0.23071000
H	-9.23024900	5.64848500	1.63839100
H	-7.50637200	5.26464800	1.86682700
O	-7.90283000	7.14731500	1.07269900
H	-11.39241300	-4.70407600	-1.78209800
H	-9.66980900	-5.14204300	-1.68042700
O	-10.99562800	-6.56666500	-0.94295400
C	-7.91580300	7.83377000	2.31353200
C	-11.11728300	-7.30121900	-2.15007800
H	-7.66019800	8.87609700	2.11062000
H	-8.90764700	7.79431800	2.78730200
H	-7.17990300	7.41500000	3.01550800
H	-11.39208800	-8.32350800	-1.88122700
H	-11.89655800	-6.88106000	-2.80261800
H	-10.17051300	-7.32074500	-2.70937200

9.4.4 (aNDI-T2)$_2$ Neutral optimized structure in water (PCM)

C	5.44868600	-3.32347600	-0.56974700
C	5.81849600	-1.91093400	-0.31595200
C	4.82456700	-0.91387200	-0.15922200
C	3.44158000	-1.24163900	-0.23941300
C	3.06878700	-2.62183500	-0.67549400
C	7.15431500	-1.58315600	-0.26372100
C	5.26560800	0.41586300	0.09816600
C	6.64962400	0.74359100	0.17897200
C	7.02014400	2.12380600	0.61573500
C	4.63909200	2.82397200	0.51102000
C	4.27084100	1.41164400	0.25594900
C	2.93416300	1.08305300	0.20548000
C	2.47537500	-0.24274300	-0.00427200
H	2.21476800	1.87614900	0.37164700
H	7.87302600	-2.37718900	-0.42794900
N	5.99024800	3.07653900	0.72227100
N	4.09679700	-3.57582300	-0.77968900
O	6.28143900	-4.22311500	-0.62091100
O	1.92579600	-2.94959000	-0.96407900
O	3.80532900	3.72294300	0.56223300
O	8.16220600	2.45548200	0.90379000
C	9.06594100	-0.04697100	-0.16651900
S	10.18249700	-1.19690700	0.55809600
C	9.76207100	0.88754100	-0.90085400
C	11.57083600	-0.39614300	-0.14894000
C	11.16224700	0.69280800	-0.89670500
H	9.27377000	1.68517600	-1.44558000
C	12.91261200	-0.87842600	0.10104400
H	11.85331400	1.32342800	-1.44434400
C	13.31852200	-2.10855400	0.57697200
S	14.31024900	0.14747800	-0.19421700
C	14.73111600	-2.23335200	0.69775800
H	12.62407400	-2.90609800	0.81627600
C	15.40066700	-1.10311400	0.31387600
H	15.22814000	-3.13032000	1.04798200
H	16.46658300	-0.92365100	0.29573400
C	7.61624800	-0.25672800	-0.05483100
C	1.02545700	-0.45929500	0.11221000
S	-0.09647900	0.65930800	-0.64583200
C	0.33869300	-1.37940400	0.87319200
C	-1.48132100	-0.14599200	0.06597100
C	-1.06308300	-1.20563200	0.85165600
H	0.83445600	-2.15710400	1.43969800
C	-2.82555300	0.30689500	-0.20639800
H	-1.74903700	-1.84114900	1.40025900
S	-4.20748200	-0.47010300	0.54145700
C	-3.24548400	1.34779800	-1.01599200
C	-5.33061000	0.63370800	-0.23649000
C	-4.64629300	1.52936200	-1.02817100
H	-2.56166900	1.96218900	-1.59063900
C	-6.78098100	0.43205700	-0.10065600
H	-5.14343700	2.29343300	-1.61182800
C	-7.24829600	-0.90136500	-0.27055800
C	-7.72961900	1.45205500	0.11787700
C	-8.58767300	-1.21673900	-0.29207500
H	-6.53242900	-1.70011400	-0.42490500
C	-9.11954500	1.14193200	0.05497000
C	-7.33975400	2.83737300	0.52037700
C	-9.00660300	-2.62942700	-0.49153200
C	-9.55440600	-0.19544700	-0.15569200
C	-10.11048700	2.14840000	0.19053800
N	-8.36166100	3.79897200	0.61721000
O	-6.19156100	3.16295500	0.79130500
N	-10.38113200	-2.87641500	-0.56998500
O	-8.19552100	-3.54318600	-0.58767900
C	-10.93353400	-0.49980800	-0.21896600

C	-9.71967300	3.55821900	0.41687400
C	-11.45851300	1.83223400	0.11241200
C	-11.37821100	-1.90249900	-0.42497900
C	-11.87585500	0.50716600	-0.08723100
O	-10.53914000	4.47085300	0.45575800
H	-12.18427400	2.63076700	0.21264700
O	-12.56361800	-2.21019700	-0.46974300
H	-12.92903800	0.25789400	-0.13999200
C	-7.95901000	5.18078800	0.95472600
C	-7.59027900	6.00496400	-0.28295500
H	-7.11130900	5.10854200	1.63572800
H	-8.80098800	5.63322300	1.47846100
C	-7.17946400	7.43688300	0.08124500
H	-6.76915600	5.50818500	-0.81385500
H	-8.44812300	6.02769300	-0.96585800
C	-6.80623200	8.27675500	-1.14406000
H	-8.00117800	7.92361800	0.62318200
H	-6.33035500	7.40497300	0.77674600
H	-6.51804900	9.29292400	-0.85660300
H	-5.96466800	7.83148200	-1.68642800
H	-7.64761700	8.35281900	-1.84190800
C	-10.81540800	-4.27378600	-0.77627000
C	-11.02081600	-5.02789400	0.54128200
H	-10.04702900	-4.75663900	-1.38035100
H	-11.74438400	-4.23419700	-1.34552700
C	-11.47430600	-6.47464700	0.31091400
H	-10.08221700	-5.01903800	1.10859700
H	-11.76728700	-4.49708000	1.14424400
C	-11.69070500	-7.24379200	1.61747300
H	-12.40462100	-6.47491600	-0.27259000
H	-10.72760300	-6.99602900	-0.30256400
H	-12.01368200	-8.27167900	1.42410400
H	-10.76838600	-7.28894600	2.20743300
H	-12.45704100	-6.76368400	2.23625900
C	6.37810700	4.45603600	1.08531500
C	6.75076800	5.30236100	-0.13623400
H	7.22029700	4.37993200	1.77265800
H	5.52755000	4.89285800	1.60845600
C	7.14744900	6.73140200	0.25391000
H	7.58022400	4.82024100	-0.66760600
H	5.89863200	5.32915700	-0.82613200
C	7.52676200	7.59255500	-0.95469600
H	6.31693800	7.20394100	0.79494300
H	7.98988100	6.69479000	0.95724800
H	7.80510400	8.60607800	-0.64879600
H	8.37664100	7.16138500	-1.49547800
H	6.69176000	7.67377800	-1.65958200
C	3.70755900	-4.95526700	-1.14158500
C	3.33386300	-5.79993400	0.08075800
H	4.55763200	-5.39343900	-1.66436800
H	2.86550200	-4.87879300	-1.82909600
C	2.93549200	-7.22883900	-0.30814400
H	4.18593800	-5.82708300	0.77071300
H	2.50496300	-5.31633400	0.61166800
C	2.55513600	-8.08841200	0.90124500
H	2.09311500	-7.19183000	-1.01152200
H	3.76545800	-7.70282400	-0.84874200
H	2.27559400	-9.10187300	0.59625300
H	3.39002800	-8.16997400	1.60621800
H	1.70575700	-7.65572500	1.44159600

References

(1) Giovannitti, A.; Maria, I. P.; Hanifi, D.; Donahue, M. J.; Bryant, D.; Barth, K. J.; Makdah, B. E.; Savva, A.; Moia, D.; Zetek, M.; Barnes, P. R. F.; Reid, O. G.; Inal, S.; Rumbles, G.; Malliaras, G. G.; Nelson, J.; Rivnay, J.; McCulloch, I. The Role of the Side Chain on the Performance of N-Type Conjugated Polymers in Aqueous Electrolytes. *Chem. Mater.* **2018**, *30* (9), 2945–2953. https://doi.org/10.1021/acs.chemmater.8b00321.

(2) Moia, D.; Giovannitti, A.; Szumska, A. A.; Maria, I. P.; Rezasoltani, E.; Sachs, M.; Schnurr, M.; Barnes, P. R. F.; McCulloch, I.; Nelson, J. Design and Evaluation of Conjugated Polymers with Polar Side Chains as Electrode Materials for Electrochemical Energy Storage in Aqueous Electrolytes. *Energy Environ. Sci.* **2019**, *12* (4), 1349–1357. https://doi.org/10.1039/c8ee03518k.

(3) Savva, A.; Ohayon, D.; Surgailis, J.; Paterson, A. F.; Hidalgo, T. C.; Chen, X.; Maria, I. P.; Paulsen, B. D.; Petty, A. J.; Rivnay, J.; McCulloch, I.; Inal, S. Solvent Engineering for High-Performance N-Type Organic Electrochemical Transistors. *Adv. Electron. Mater.* **2019**, *5*, 1900249. https://doi.org/10.1002/aelm.201900249.

(4) Giovannitti, A.; Sbircea, D. T.; Inal, S.; Nielsen, C. B.; Bandiello, E.; Hanifi, D. A.; Sessolo, M.; Malliaras, G. G.; McCulloch, I.; Rivnay, J. Controlling the Mode of Operation of Organic Transistors through Side-Chain Engineering. *Proc. Natl. Acad. Sci. U. S. A.* **2016**, *113* (43), 12017–12022. https://doi.org/10.1073/pnas.1608780113.

(5) Khodagholy, D.; Rivnay, J.; Sessolo, M.; Gurfinkel, M.; Leleux, P.; Jimison, L. H.; Stavrinidou, E.; Herve, T.; Sanaur, S.; Owens, R. M.; Malliaras, G. G. High Transconductance Organic Electrochemical Transistors. *Nat. Commun.* **2013**, *4*, 1–6. https://doi.org/10.1038/ncomms3133.

(6) Frisch, G. W. Trucks, H. B. Schlegel, G. E. Scuseria, M. A. Robb, J. R. Cheeseman, G. Scalmani, V. Barone, G. A. Petersson, H. Nakatsuji, X. Li, M. Caricato, A. V. Marenich, J. Bloino, B. G. Janesko, R. Gomperts, B. Mennucci, H. P. Hratchian, J. V. O. Gaussian 16, Revision B.01. 2016. https://doi.org/111.

(7) Guiglion, P.; Butchosa, C.; Zwijnenburg, M. A. Polymeric Watersplitting Photocatalysts; A Computational Perspective on the Water Oxidation Conundrum. *Journal of Materials Chemistry A*. Royal Society of Chemistry August 2014, pp 11996–12004. https://doi.org/10.1039/c4ta02044h.

(8) Guiglion, P.; Monti, A.; Zwijnenburg, M. A. Validating a Density Functional Theory Approach for Predicting the Redox Potentials Associated with Charge Carriers and Excitons in Polymeric Photocatalysts. *J. Phys. Chem. C* **2017**, *121* (3), 1498–1506. https://doi.org/10.1021/acs.jpcc.6b11133.

(9) Chen, Z.; Zheng, Y.; Yan, H.; Facchetti, A. Naphthalenedicarboximide- vs Perylenedicarboximide-Based Copolymers. Synthesis and Semiconducting Properties in Bottom-Gate N-Channel Organic Transistors. *J. Am. Chem. Soc.* **2009**, *131* (1), 8–9. https://doi.org/10.1021/ja805407g.

(10) Chen, X.; Marks, A.; Paulsen, B. D.; Wu, R.; Rashid, R. B.; Chen, H.; Alsufyani, M.; Rivnay, J.; McCulloch, I. N -Type Rigid Semiconducting Polymers Bearing Oligo(Ethylene Glycol) Side Chains for High-Performance Organic Electrochemical

Transistors. *Angew. Chemie Int. Ed.* **2021**, *60* (17), 9368–9373. https://doi.org/10.1002/anie.202013998.
